# Total syntheses of naturally occurring antiviral indolosesquiterpene alkaloids, xiamycins C–F *via* Csp^3^–H functionalization†

**DOI:** 10.1039/d2sc03479d

**Published:** 2022-09-21

**Authors:** Mintu Munda, Rhituparna Nandi, Vipin R. Gavit, Sourav Kundu, Sovan Niyogi, Alakesh Bisai

**Affiliations:** Department of Chemistry, Indian Institute of Science Education and Research Bhopal Bhopal Bypass Road Bhopal 462 066 Madhya Pradesh India alakeshb@gmail.com; Department of Chemistry, Indian Institute of Science Education and Research Kolkata Mohanpur Campus, Nadia Kalyani 741 246 West Bengal India alakesh@iiserkol.ac.in

## Abstract

Concise total syntheses of naturally occurring antiviral indolosesquiterpene alkaloids, xiamycin C (2a), D (2b), E (2c) and F (2d), have been achieved *via* a late-stage oxidative δ-Csp^3^–H functionalization of an advanced pentacyclic enone intermediate 8. This strategy takes advantage of *ipso*-nitration of naturally occurring abietane diterpenoids to synthesize *o*-bromo nitroarene derivative 11. A Suzuki–Miyaura coupling of 11 with phenylboronic acid followed by Cadogan's ring closure provided a modular approach to a carbazole ring required for a functionalized pentacyclic core of indolosesquiterpene alkaloids.

## Abstract 2

Enantioenriched enone 8 was synthesized *via* three key transformations: *ipso*-nitration of abietane diterpenoids to furnish *o*-bromo nitroarene 11, Suzuki coupling with phenylboronic acid, and Cadogan's reductive ring closure to craft a carbazole ring.

## Graphical abstract

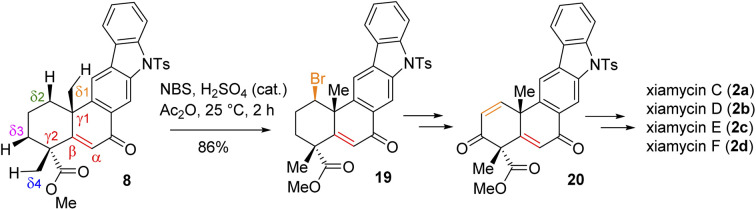

## Introduction

**Fig. 1:**
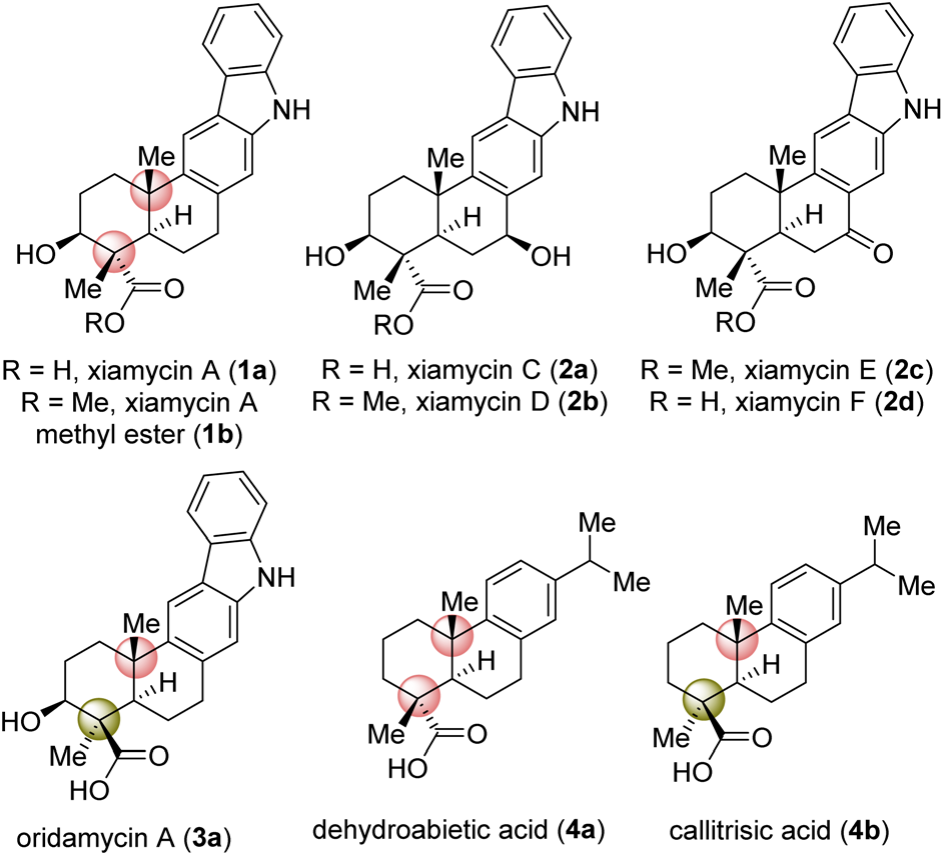
Naturally occurring indolosesquiterpene alkaloids 1–3.

Indolosesquiterpene alkaloids (1a, 2a–d, and 3a; Fig. 1) are a growing class of architecturally complex secondary metabolites that were first isolated from a range of *Streptomyces* species in 2010.^1*a*,*b*^ A number of important biological activities such as antimicrobial, antiviral, antitumor, immunomodulatory, and enzyme inhibitory activities are displayed by the xiamycin family of alkaloids.^1*c*^ In 2010, xiamycin A (1a) and its methyl ester (1b), displaying anti-HIV and antibiotic activities,^2*a*^ were isolated by Hertweck *et al.* from *Streptomyces* sp. GT2002/15032a and HKI0595,^2*b*^ endophytes from the mangrove plant *Bruguiera gymnorrhiza*^2*a*^ and *Kandelia candel*,^2*b*^ respectively. Later, 1a was isolated by Zhang *et al.*^3^ In 2016, Kim *et al.*^4*b*^ reported the isolation of structurally related xiamycins C (2a), D (2b), E (2c) and F (2d) from a *Streptomyces* sp. (#HK18) culture inhabiting the topsoil in a Korean solar saltern. Xiamycin D (2b) was found to show a potent inhibitory effect on porcine epidemic diarrhea virus (PEDV) replication with an EC_50_ = 0.93 μM and low cytotoxicity (CC_50_ = 56.03 μM), indicating high potential as an antiviral agent specifically against PEDV-related viruses.^4*a*,*b*^ Structurally, these alkaloids are composed of an architecturally intriguing pentacyclic framework with four contiguous stereogenic centers at the periphery of a *trans*-decalin scaffold embedded with carbazole units. Importantly, two out of four stereogenic centers feature challenging all-carbon quaternary centers. Biogenetically, xiamycin A (1a) could be derived from another secondary metabolite, indosespene *via* a C–C bond formation, followed by oxidation to form the carbazole scaffold.^5^ The emerging biological activity of these indolosesquiterpenoids drew attention from the synthetic community for their efficient total syntheses. The total synthesis of dixiamycin B achieved by carrying out electrochemical oxidation of xiamycin A (1a) has been demonstrated by Baran and co-workers.^6^ Their approach mainly relies on the construction of the *trans*-decalin system, followed by coupling of this system with the carbazole ring. Similarly, an elegant synthetic strategy has been developed by Li *et al.* for the total syntheses of xiamycin A and oridamycins.^7^

The strategy developed by these investigators for the construction of the carbazole ring involves 6π-electrocyclization/aromatization and indole C2–H bond activation/Heck annulation, whereas the construction of the *trans*-decalin system was achieved by carrying out two diastereochemically complementary radical cyclizations, mediated by Ti(iii) and Mn(iii), respectively.^7^ In 2019, the Sarpong group reported the total syntheses of xiamycins A, C, F, and H from (*R*)-carvone, using a photoinduced benzannulation sequence to forge the carbazole core.^8^ Very recently, Dethe and co-workers have reported the first total synthesis of xiamycins D (2b) and E (2c) *via* a key Michael addition of an indole onto a diterpene moiety, oxidative Heck/aromatization, and highly fascinating regioselective sp^3^ C–H activation.^9^ In spite of these existing elegant strategies, a concise asymmetric approach to most of the congeners of the xiamycin family would be very interesting. Herein, we report a collective asymmetric total synthesis of xiamycins C (2a), D (2b), E (2c), and F (2d) *via* a key regioselective sp^3^ C–H activation. Our synthesis is complementary to previous approaches to xiamycin-type indolosesquiterpenoids.

## Results and discussion

**Scheme 1:**
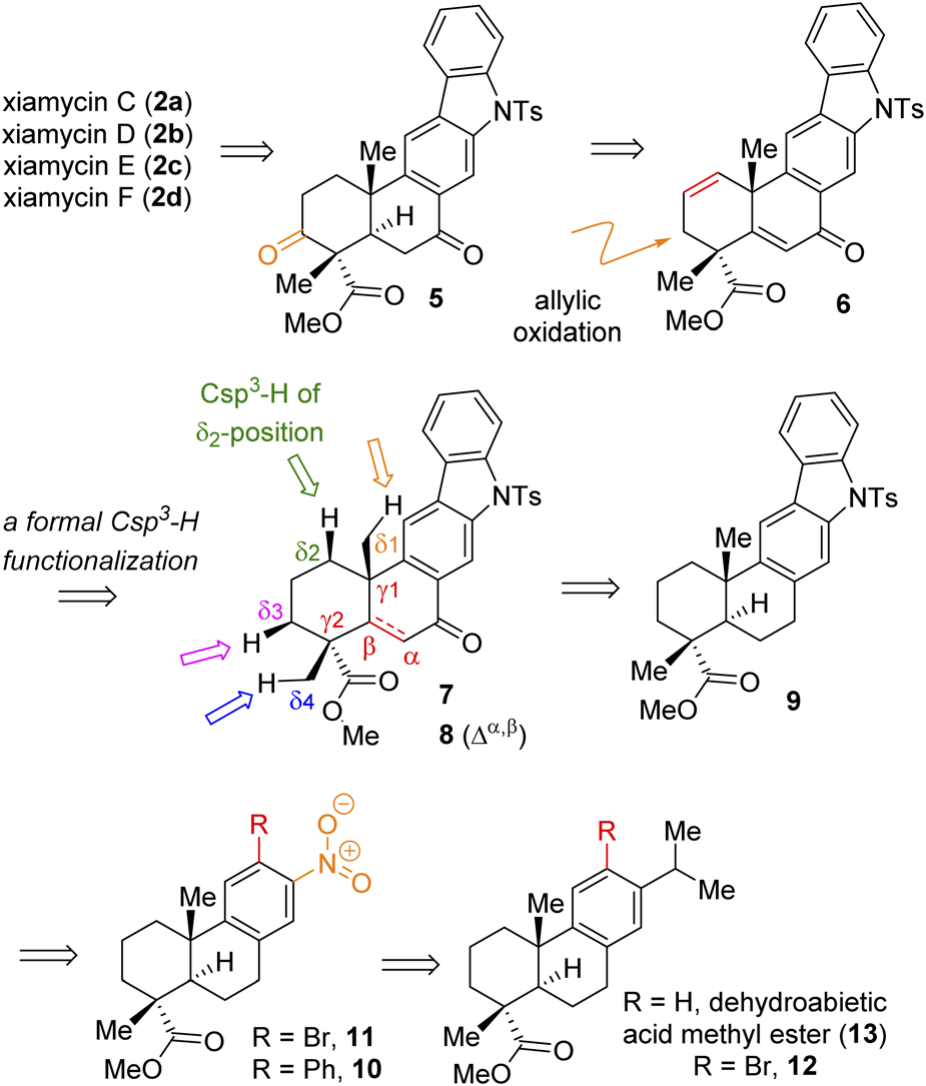
Retrosynthetic analysis of indolosesquiterpene alkaloid xiamycins C (2a), D (2b), E (2c), and F (2d).

Based on their stereochemical resemblances with naturally occurring diterpenoid, dehydroabietic acid (4a) (Fig. 1), and indolosesquiterpene alkaloids 2a–d, we envisioned a unified approach to these targets (Scheme 1). Retrosynthetically, we envisioned to access the highly functionalized pentacyclic core of xiamycins from enone-olefin 6 (Scheme 1) as an advanced intermediate that could be elaborated to diketone 5*via* allylic oxidation and hydrogenation. We thought of exploring regioselective sp^3^ C–H activation^10^ at the δ-position of ketone 7 or α,β-unsaturated carbonyl 8. As there are four different δ-positions of these carbonyl compounds, the exploration of such reactivity would be challenging but worth pursuing. Intermediate 6 could be accessed from a key site-selective formal Csp^3^–H functionalization of 8*via* a secondary bromide. Enone 8 could be synthesized from 9*via* oxidation, which in turn could be accessed from 2-phenylnitrobenzene 10*via* Cadogan's ring closure^11^ (Scheme 1). Furthermore, *o*-bromo nitroarene 11 could be achieved from *o*-bromo isopropylarene 12*via ipso*-nitration,^12^ which in turn could be synthesized from naturally occurring diterpenoid, dehydroabietic acid methyl ester 13 (Scheme 1).

**Scheme 2:**
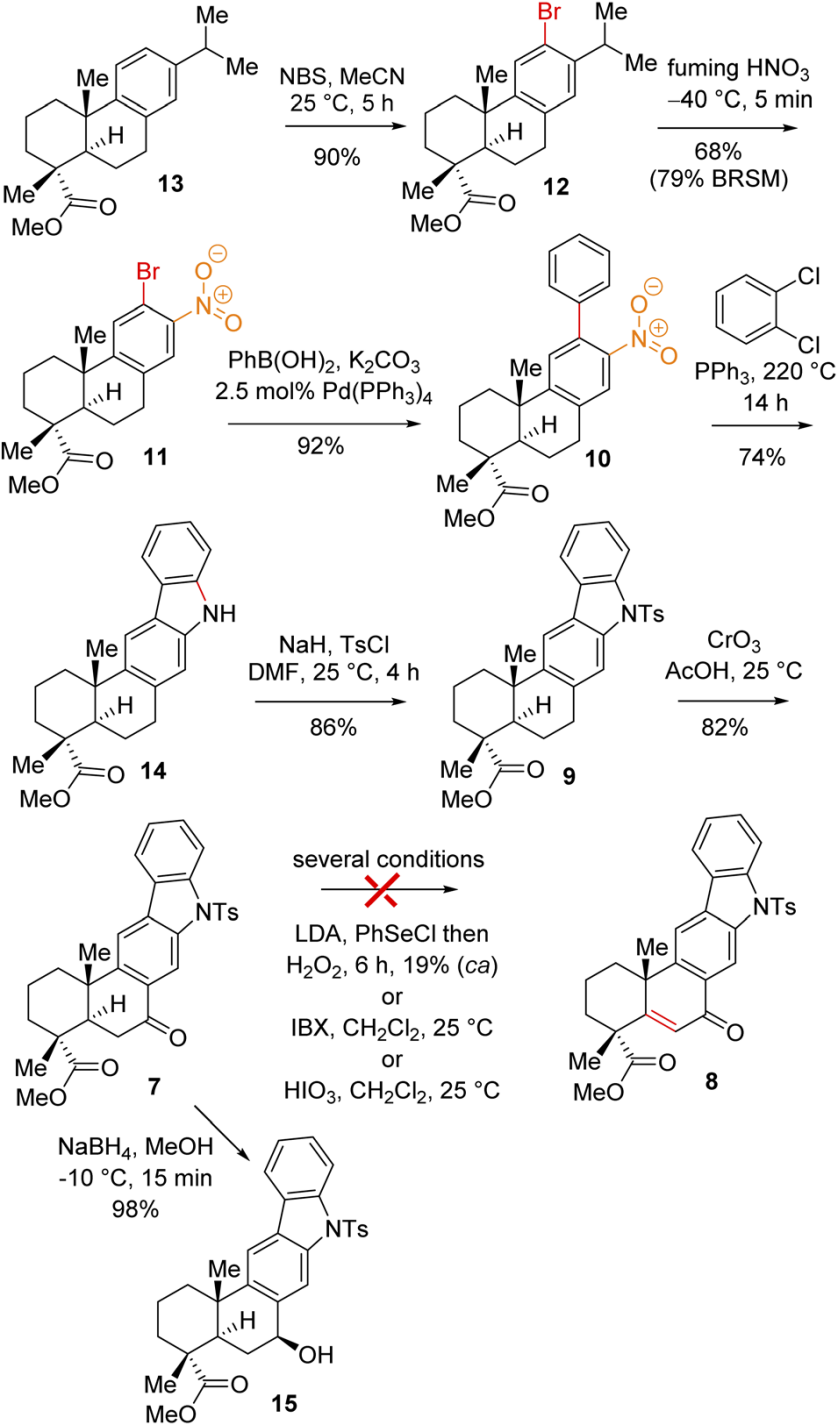
Synthesis of the indolosesquiterpene scaffold (9).

**Scheme 3:**
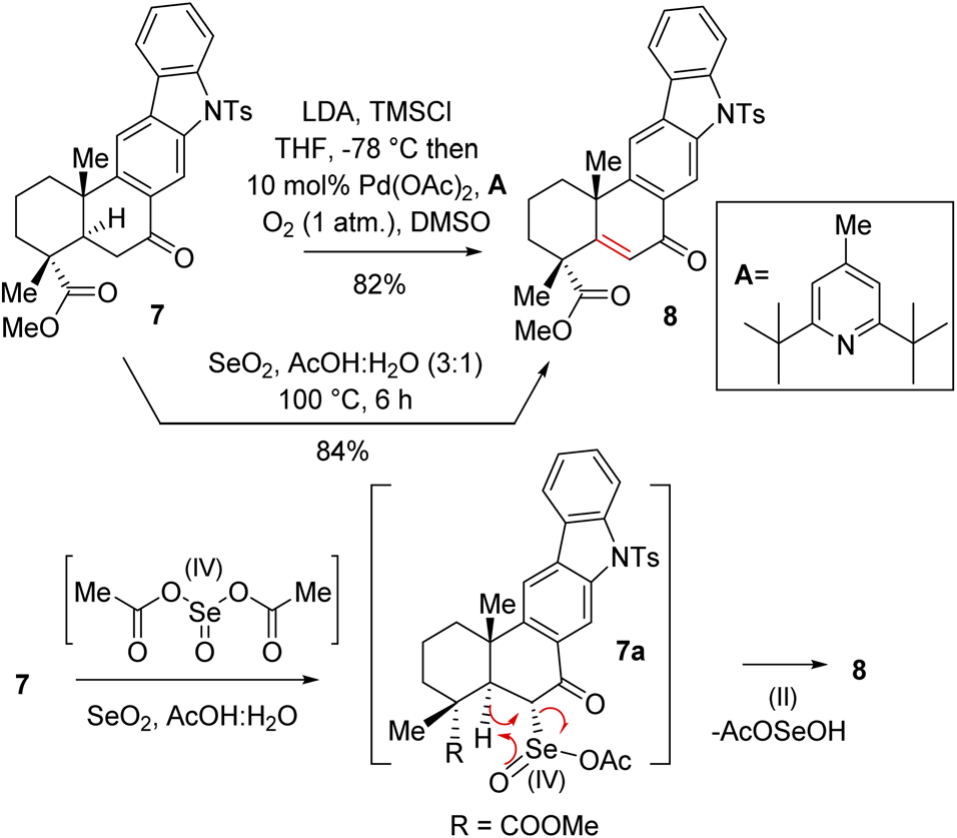
Synthesis of enone 8.

Thus, A-ring functionalization could be a key strategy for a collective total synthesis of naturally occurring indolosesquiterpene alkaloids. Commercially available abietic acid was converted to dehydroabietic acid at 240 °C for 4 h, followed by methylation by using dimethyl sulphate [(MeO)_2_SO_2_], which furnished 13. The bromination of this compound with NBS afforded bromoarene 12 in 90% yield (Scheme 2). Aromatic electrophilic *ipso*-nitration of 12 (see the ESI† for detailed optimization) could furnish the required *o*-bromo nitroarene 11 in 68% yield (79% BRSM).^12*e*^ Next, Suzuki–Miyaura coupling of 11 with phenylboronic acid afforded 2-phenylnitrobenzene 10 in 92% yield. At this stage, Cadogan's ring closure^11^ was carried out to get deoxyxiamycin A methyl ester 14 in 74% yield. Furthermore, *N*-tosylation of 14 (see, compound 9) followed by benzylic oxidation using CrO_3_ in acetic acid at room temperature furnished ketone 7 in 70% yield over 2 steps (Scheme 2). At this stage, several oxidative conditions such as selenylation followed by H_2_O_2_ treatment (19% yield) and oxidation using IBX,^13^ HIO_3_*etc.* proved to be unsuccessful and 42–49% starting materials were isolated along with the decomposition of the rest of the mass balance. To our delight, Saegusa–Ito oxidation^14^ of ketone 7 (*via* silyl enol ether) provided the corresponding enone 8 in 82% yield in the presence of catalytic Pd(OAc)_2_ under 1 atm of oxygen and 2,6-di-*tert*-butyl 4-methyl pyridine as the base (Scheme 3). A two-step protocol following α-bromination with PTAB (phenyl trimethyl ammonium tribromide) (see the ESI†) followed by β-elimination afforded 8 in 79% yield. Gratifyingly, a one-step procedure using SeO_2_ under refluxing AcOH and water^15^ at 100 °C for 6 h afforded 8 in 84% yield that avoids expensive palladium catalysts. A tentative mechanism involving a two-electron oxidation using selenium dioxide is shown through intermediate 7a (Scheme 3). Later, an intramolecular elimination through a five-membered cyclic transition state would result in enone compound 8.

**Scheme 4:**
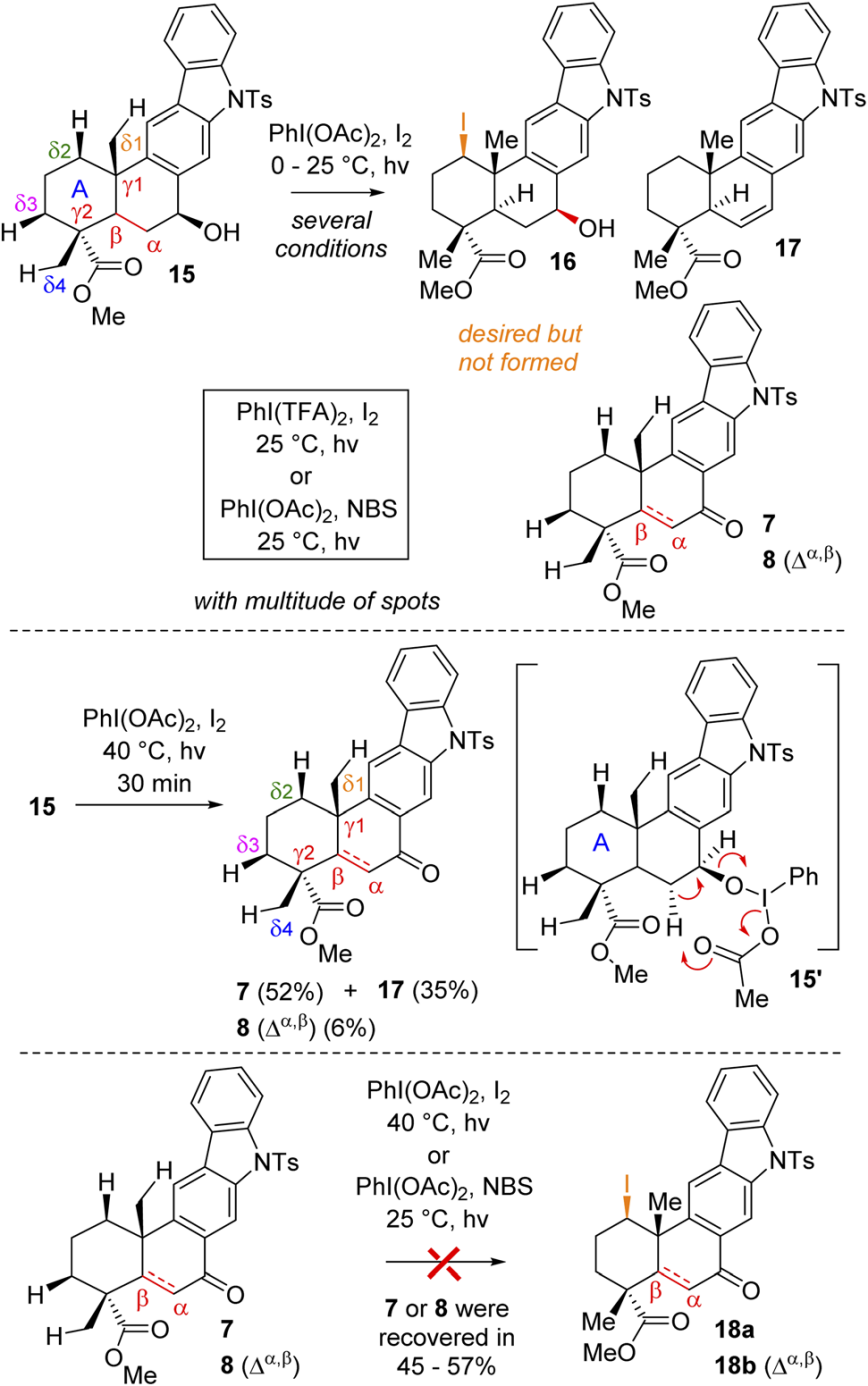
Attempted δ-Csp^3^–H functionalization of (+)-15, (+)-7, and (+)-8.

At this stage, we were all set for the site-selective formal Csp^3^–H functionalization of compounds 7, 8, and 15 to a functionalize A-ring. In this regard, Baran's synthesis of methyl atisenoate^16^ and isoatisine *via* a late-stage Suárez^17^ modified Hofmann–Löffler–Freytag (HLF) reaction^18^ attracted our attention. Initially, we envisioned that the secondary alcohol of compound 15 might be able to direct and help in the Csp^3^–H functionalization. Thus, we tried to oxidize the A-ring of compounds 15*via* Csp^3^–H functionalization using a stoichiometric amount of iodine with PhI(OAc)_2_ in the presence of light (Scheme 4) (also see the ESI† for detailed optimization). However, under several conditions, it turns out to be rather difficult to functionalize because of the multitude of spots on the TLC probably due to competitive reactions. It was observed that at 40 °C in the presence of light this reaction led to the formation of benzylic ketone 7 (52% yield) along with enone 8 (6% yield) and olefin 17 (35%) (Scheme 4).

Since compound 15 is not suitable for Csp^3^–H functionalization of the A-ring and we could only isolate products with oxidation at the B-ring, we turned our attention towards using carbonyls such as compounds 7 and 8 for further studies. These compounds are challenging substrates in a sense that they have a number of sites capable of Csp^3^–H functionalization (see Csp^3^–H at the *δ*_1_*vs. δ*_2_*vs. δ*_3_*vs. δ*_4_ positions of 7 and 8, respectively). However, we didn't have much success in using hypervalent iodine reagents to affect the Csp^3^–H functionalization (Scheme 4). In the case of compound 7, we could isolate the formation of enone 8 in 38% yield along with 35% recovery of ketone and decomposition of the rest of the mass balance. Thus, we turned our attention for a formal Csp^3^–H functionalization of 8, followed by trapping with a ‘Br^+^’ species as reported for an abietane skeleton by Tahara *et al.*^19^ For our studies, we have utilized a number of brominating sources such as Br_2_, NBS, and DBDMH for the stereoselective bromination to afford compound 19*via* a formal Csp^3^–H bromination (Table 1).

**Table 1:** Optimization of Csp^3^–H bromination of (+)-8

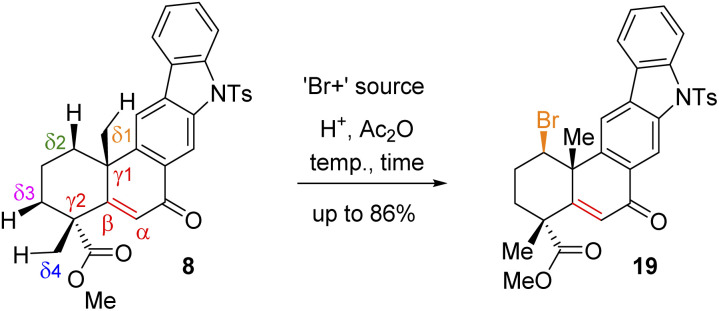					
S. no.	Halogen source	Acid	Solvent	Temp.	Yield^a^
1	Br_2_ (1.2 eq.)	H_2_SO_4_ (cat.)	CH_2_Cl_2_	25 °C, 6 h	Complex
2	NBS (1.2 eq.)	H_2_SO_4_ (cat.)	CH_2_Cl_2_	25 °C, 5 h	18%^b^
3	NBS (1.2 eq.)	H_2_SO_4_ (cat.)	(CH_2_Cl)_2_	25 °C, 6 h	29%^b^
4	DBDMH (1.2 eq.)	H_2_SO_4_ (cat.)	(CH_2_Cl)_2_	25 °C, 9 h	21%^b^
5	NBS (1.2 eq.)	AcOH (cat.)	CH_2_Cl_2_	35 °C, 6 h	ND^b^
6	NBS (1.2 eq.)	H_2_SO_4_ (cat.)	(CH_2_Cl)_2_	60 °C, 7 h	34%^b^
7	NBS (1.2 eq.)	TfOH (cat.)	(CH_2_Cl)_2_	40 °C, 6 h	ND^b^
8	NBS (1.2 eq.)	H_2_SO_4_ (cat.)	CHCl_3_	25 °C, 2 h	34%
9	NBS (1.2 eq.)	H_2_SO_4_ (cat.)	CHCl_3_	40 °C, 1 h	39%
**10**	**NBS (1.2 eq.)**	**H**_**2**_ **SO**_**4**_ **(cat.)**	**Ac**_**2**_ **O**	**25 °C, 2 h**	**86%**
11	NBS (1.2 eq.)	H_2_SO_4_ (cat.)	Ac_2_O	45 °C, 1 h	73%

a All reactions were done using 0.05 mmol of 8 and yields are reported after column purification.

b Starting materials were recovered in 35–52%.

**Scheme 5:**
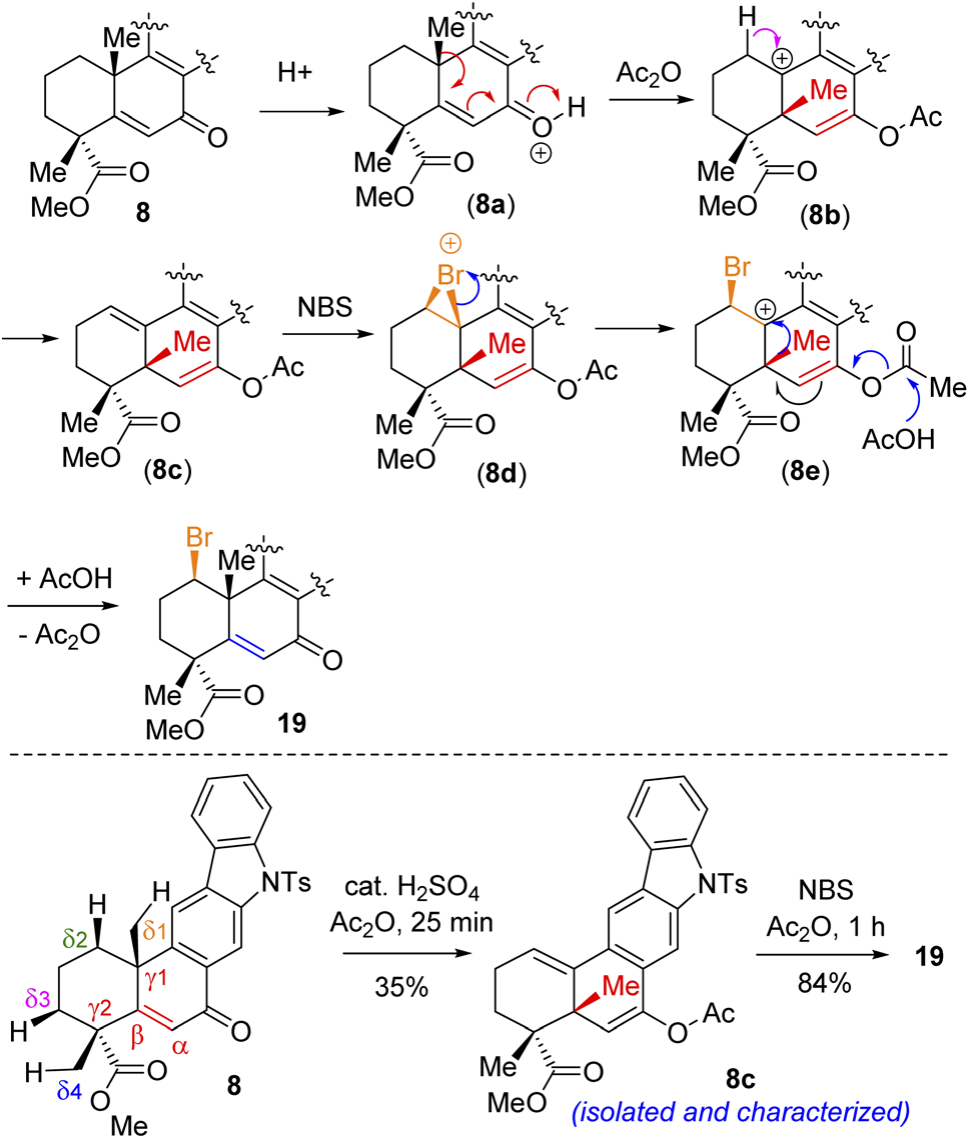
Tentative mechanism of Csp^3^–H bromination of (+)-8.

Following exhaustive optimization, it was found that Csp^3^–H functionalization of 8 could be promoted with NBS in the presence of catalytic sulfuric acid in acetic anhydride to afford product 19 in 86% isolated yield (Table 1). A tentative mechanism of Csp^3^–H functionalization of 8 with NBS in the presence of catalytic sulfuric acid is shown in Scheme 5. It is proposed that upon the activation of enone with acetic anhydride, a *syn*-selective 1,2-migration of the angular methyl group would result in olefin intermediate 8c*via* the formation of 3° carbocation intermediate 8b (Scheme 5). Next, bromonium ion formation from the convex face (see 8d) followed by another *syn*-selective 1,2-migration of the methyl group *via* another 3° carbocation intermediate 8e could form compound 19 as a single diastereomer (Scheme 5). To validate this mechanism, an attempt was made to isolate enol acetate intermediate 8c from a reaction of enone 8 in the presence of catalytic sulfuric acid in acetic anhydride. To our pleasure, we were able to isolate enol acetate 8c in 35% yield, when the reaction was conducted in the absence of NBS.^20^ Furthermore, the enol acetate 8c was converted to the secondary bromide 19 when reacted with 1.2 equivalents of NBS (Scheme 5). Thus, the proposed mechanism with the migration of the methyl group from one angular position of the decaline system back and forth to the other explains the outcome of the Csp^3^–H functionalization process.^21^

**Scheme 6:**
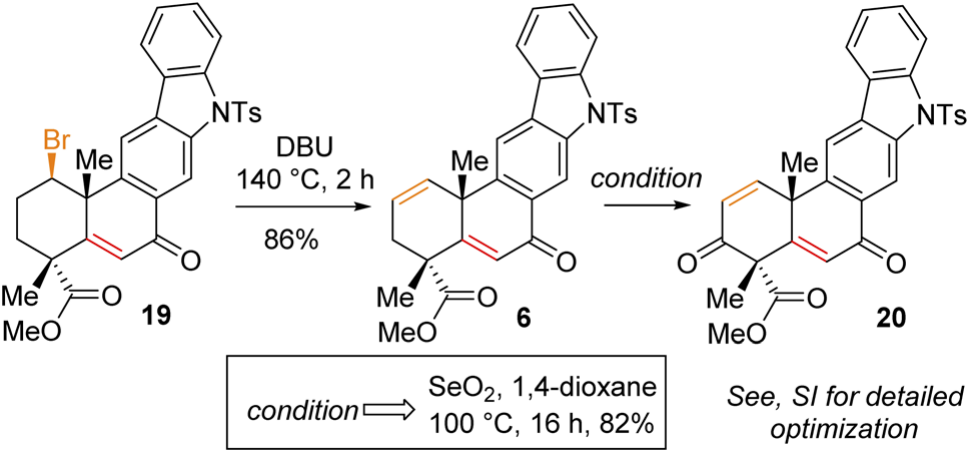
Allylic oxidation of 6.

**Scheme 7:**
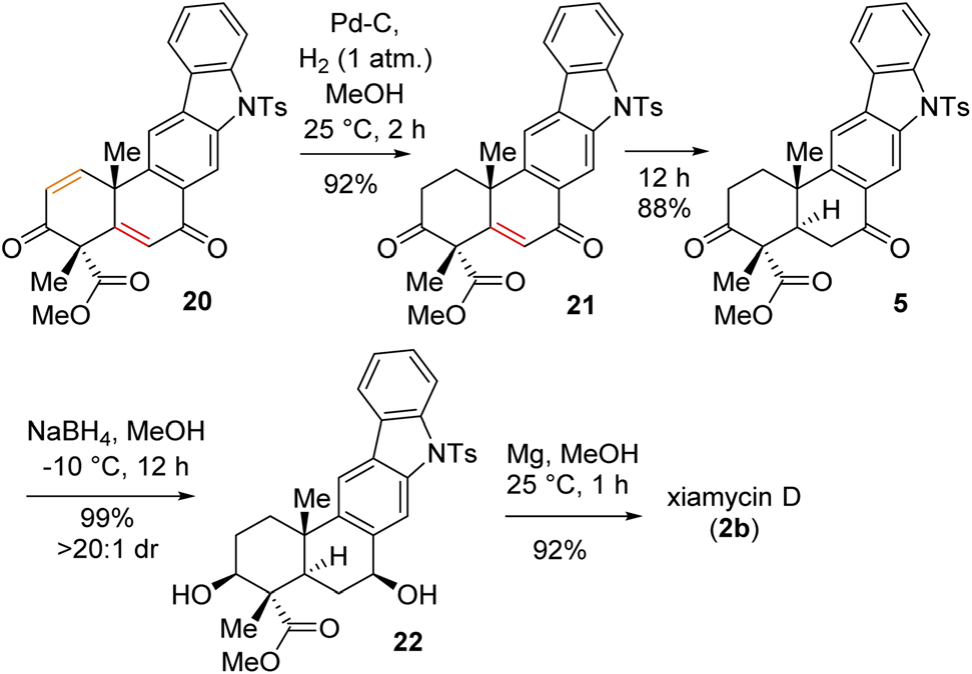
Total synthesis of xiamycin D (2b).

Having secured compound 19 in hand, our effort was thereafter to elaborate this to bis-enone derivative 20 (Scheme 6). In this regard, an E2-elimination of secondary bromide 19 leads to the formation of olefin 6 in 86% yield. Furthermore, allylic oxidation of olefin 6 with SeO_2_ (see the ESI† for detailed optimization) afforded bis-enone derivative 20 in 82% yield (Scheme 6). With bis-enone derivative 20 in hand, it was hydrogenated to access diketone 5 in 88% yield over 12 h (Scheme 7). It is worth mentioning that a chemoselective hydrogenation furnished 21 in 92% yield as a sole product under hydrogenation conditions for 2 h. Next, highly diastereoselective reduction of diketone 5 by NaBH_4_ at −10 °C for 12 h furnished diol 22 in an almost quantitative yield with >20 : 1 dr (Scheme 7). This reaction represents a simultaneous double stereoselective reduction of ketone 5 to form a sole diastereomer (as determined by ^1^H-NMR studies of the crude product) in favour of the stereoisomer required for the synthesis of the natural product, xiamycin D (2b). The subsequent detosylation of 22 with Mg powder in methanol completed the total synthesis of xiamycin D (2b) in 92% yield (Scheme 7).

**Scheme 8:**
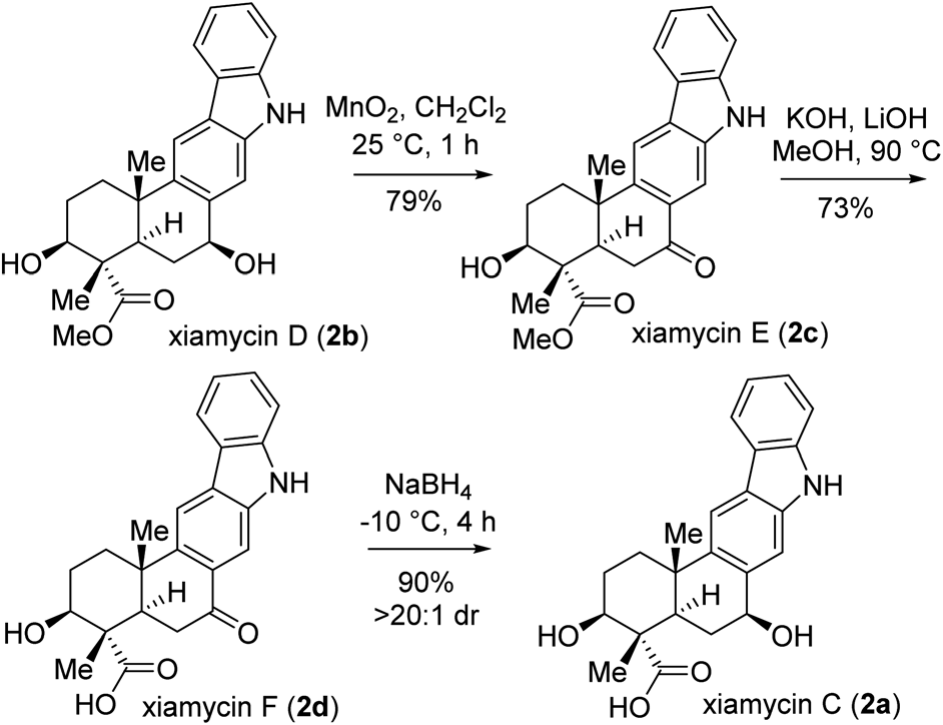
Total syntheses of xiamycins C (2a), E (2c), and F (2d).

Next, the total synthesis of xiamycin E (2c) and xiamycin F (2d), having benzylic ketone, was undertaken. In this regard, a highly chemoselective oxidation with MnO_2_ provided the benzylic ketone, thereby completing the total synthesis of xiamycin E (2c) in 79% yield (Scheme 8). Furthermore, a saponification of xiamycin E (2c) with KOH and LiOH in MeOH/H_2_O under refluxing conditions completed the total synthesis of xiamycin F (2d) in 73% yield. Finally, highly diastereoselective reduction of the ketone functionality by NaBH_4_ at −10 °C for 4 h of xiamycin F (2d) completed the total synthesis of xiamycin C (2a) in 90% yield with >20 : 1 dr (Scheme 8).^20^

## Conclusions

In conclusion, we have accomplished the total syntheses of naturally occurring antiviral indolosesquiterpene alkaloids, xiamycins C (2a), D (2b), E (2c), and F (2d) *via* a late-stage oxidative δ-Csp^3^–H functionalization of pentacyclic enone 8. The synthesis of the pentacyclic functionalized core of indolosesquiterpene alkaloids takes advantage of *ipso*-nitration of naturally occurring abietane diterpenoids followed by a Suzuki–Miyaura reaction and Cadogan's ring closure. Further utilization of our approach to other congeners of naturally occurring indolosesquiterpene alkaloids is currently under active investigation.

## Data availability

Experimental details and spectral analysis are available free of charge from the ESI† available with this article.

## Author contributions

Bisai, A. designed the project and written the manuscript. Munda, M.; Nandi, R.; Gavit, V. R.; Kundu, S.; and Niyogi, S. have carried out all experiments. Munda, M. and Nandi, R. have revised the manuscript.

## Conflicts of interest

There are no conflicts to declare.

## Supplementary Material

SC-013-D2SC03479D-s001
