# Supplementary material for: Total syntheses of naturally occurring antiviral indolosesquiterpene alkaloids, xiamycins C–F via Csp3–H functionalization
Source: Chem Sci. 2022 Sep 21;13(39):11666–71. doi: 10.1039/d2sc03479d (PMC9555729; doi:10.1039/d2sc03479d)
Supplement: SC-013-D2SC03479D-s001 [file SC-013-D2SC03479D-s001.pdf]

Electronic Supplementary Material (ESI) for Chemical Science.
This journal is © The Royal Society of Chemistry 2022

Supplemental material for:

Total Syntheses of Naturally Occurring Antiviral Indolosesquiterpene Alkaloids, Xiamycins C-F via Csp^3-H Functionalization

Mintu Munda,[a£] Rhituparna Nandi,[a£] Vipin R. Gavit,[a] Sourav Kundu,[a] Sovan Niyogi,[b] and Alakesh Bisai[*a,b]

[a]Department of Chemistry, Indian Institute of Science Education and Research Bhopal, Bhopal Bypass Road, Bhopal, MP - 462 066, India.

[b]Department of Chemical Sciences, Indian Institute of Science Education and Research Kolkata, Mohanpur, Nadia, WB - 741 246, India.

e-Mail: *alakesh@iiserkol.ac.in, alakeshb@gmail.com*

Table of Contents

Materials and Methods	S2
Synthesis of Methyl dehydroabietate from Abietic acid	S3-S4
Synthesis of (+)-**12** and (+)-**11**	S4-S7
Synthesis of (+)-**10** and (+)-**14**	S7-S10
Synthesis of (+)-**9**, (+)-**7**, (+)-**15** and (+)-**8**	S10-S16
General procedure for δ-Csp^3-H Activation and optimization	S16-S19
Synthesis of (–)-**19**, (+)-**6** and (+)-**20**	S19-S26
Synthesis of (+)-**21**, (+)-**5** and (+)-**22**	S26-S30
Total Synthesis of (+)-Xiamycin D [(+)-**2b**]	S30-S34
Total Synthesis of (+)-Xiamycin E [(+)-**2c**]	S35-S39
Total Synthesis of (+)-Xiamycin F [(+)-**2d**]	S39-S43
Total Synthesis of (+)-Xiamycin C [(+)-**2a**]	S43-S47
Spectral Data	S48-S95
References	S96

Materials and Methods

Unless otherwise stated, reactions were carried out using oven dried glass ware with Teflon coated magnetic stirring bars were used to stir the reactions. The Syringe was used to transfer the solvents and liquid reagents. Tetrahydrofuran (THF) Diethyl ether (Et_2O) were distilled over sodium/benzophenone ketyl. Dichloromethane CH_2Cl_2) was distilled over calcium hydride. All other solvents like MeOH, EtOAc, DMF, Dichloroethane (DCE) and reagents were used as received. Reaction temperatures above 25 °C were maintained by using oil bath on a magnetic stirrer. Thin layer chromatography (TLC) analysis was performed by using silica gel precoated plates (0.25 mm) 60 (F-254), Visualized by UV irradiation, yellow dip stain and other stains. Silica gel of particle size 230-400 and 100-200 mesh were used to perform flash chromatography. Digital melting point apparatus is used to record the melting points. ^{1}H-NMR spectra was recorded by using 400, 500 MHz spectrometers, ^{13}C-NMR operating frequencies are 100, 125 MHz respectively. Chemical shifts (δ) are reported in ppm relative to the residual solvents $CDCl_3$ signal (δ = 7.28 for ^{1}H NMR and δ = 77.0 for ^{13}C NMR) and CD_3OD signal (δ = 3.33 for ^{1}H NMR and δ = 49.0 for ^{13}C NMR). Data for ^{1}H NMR spectra are reported as follows: chemical shift (multiplicity, coupling constants, and number of hydrogen). Abbreviations are as follows: s (singlet), d (doublet), t (triplet), q (quartet), m (multiplet), br (broad). IR spectra were recorded on a FT-IR system (Spectrum BX) and are reported in frequency of absorption (cm^{-1}). Only selected IR absorbencies are reported. High Resolution Mass Spectrometry (HRMS) data was recorded on MicrOTOF-Q-II mass spectrometer using methanol as solvent. Optical rotations were measured on an automatic polarimeter.

Synthesis of Methyl dehydroabietate (+)-**13 from Abietic acid:**

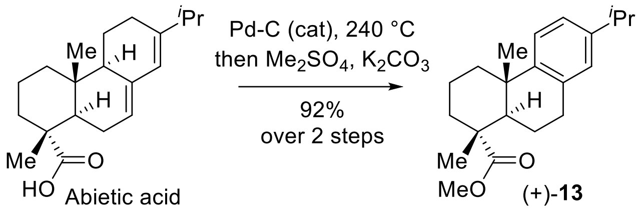

Abietic acid (9.0 g, 29.7 mmol, 1.0 equiv.) was taken in an oven-dried 100 mL round-bottom (RB) flask and Pd/C (90 mg, 0.01 % w/w) was added at room temperature. The RB flask was equipped with a condenser and it was allowed to stir at 240 ºC for 4 h on a pre-heated oil-bath. After complete conversion of the starting material (judged by running TLC), the reaction mixture was allowed to cool to 25 ºC and the crude was charged for the next step without any purification.

To the above reaction mixture was taken in acetone (50 mL) and K_2CO_3 (4.5 g, 32.7 mmol, 1.1 equiv.) was added portion wise. To this solution was added Me_2SO_4 (3.1 mL, 32.7 mmol, 1.1 equiv.) and it was allowed to reflux at 60 ºC for 2 h. After complete consumption of the starting material (monitored by TLC), the reaction mixture was filtered and the solid was washed with EtOAc (50 mL X 2). The combined organic layers were concentrated in a rotary evaporator under reduced pressure and crude product was purified through column chromatography with 10% EtOAc in *n*-hexane to afford the methyl dehydroabietate (+)-**13** (8.8 g, 92% yield over 2 steps).

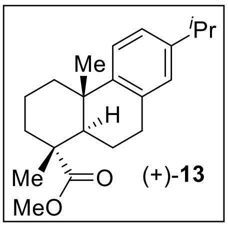

Methyl (1*R*,4a*S*,10a*R*)-**7-isopropyl-1,4a-dimethyl-1,2,3,4,4a,9,10,10a-octahydrophenanthrene-1-carboxylate** [(+)-**13**]: (+)-**13** was obtained as colorless oil (29.7 mmol scale of reaction; 8.8 g; 92% yield over 2 steps). R_f = 0.3 (10% EtOAc in *n*-hexane).

^{1}**H NMR** (500 MHz, $CDCl_3$) δ 7.20 (d, *J* = 8.2 Hz, 1H), 7.04 (dd, *J* = 8.2, 2.1 Hz, 1H), 6.92 (d, *J* = 2.0 Hz, 1H), 3.70 (s, 3H), 2.95 – 2.89 (m, 2H), 2.88 – 2.83 (m, 1H), 2.36 – 2.31 (m, 1H),

2.28 (dd, *J* = 12.5, 2.2 Hz, 1H), 1.90 – 1.85 (m, 1H), 1.82 (td, *J* = 5.0, 1.8 Hz, 2H), 1.78 – 1.73 (m, 2H), 1.69 – 1.67 (m, 1H), 1.55 (dd, J = 12.6, 4.4 Hz, 1H), 1.47 – 1.43 (m, 1H), 1.32 (s, 3H), 1.27 (s, 3H), 1.25 (d, *J* = 2.9 Hz, 6H).

^{13}C NMR (125 MHz, $CDCl_3$) δ 179.1, 146.9, 145.7, 134.7, 126.9, 124.1, 123.9, 51.9, 47.7, 44.9, 38.0, 37.0, 36.7, 33.5, 30.0, 25.1, 24.0, 21.7, 18.6, 16.5.

IR (neat) υ_{max} 2958, 2957, 2866, 2369, 1726, 1498, 1243, 1121, 915, 768 cm^{-1}.
HRMS (ESI) *m/z*: $[M + H]^+$ calcd. for $[C_{21}H_{30}O_2 + H]^+$ 315.2319, found 315.2330.

$[\alpha]^{25}_{589}$ = +32.2 (*c* = 0.75, $CHCl_3$).

Aromatic Electrophilic Bromination of (+)-**13**:

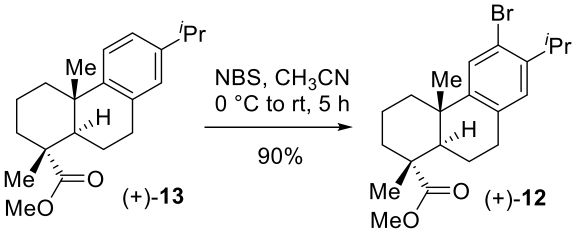

In an oven dried round-bottom flask (+)-**13** (6.0 g, 19.1 mmol, 1.0 equiv.) was taken in 65 mL of CH_3CN. To the reaction mixture NBS (4.1 g, 22.9 mmol, 1.2 equiv.) was added at 25 °C and stirred at the same temperature for 5 h. After completion of the reaction (monitored by TLC), saturated aqueous $Na_2S_2O_3$ solution was added to the reaction mixture. The reaction mixture was then partitioned and extracted with EtOAc (50 mL X 2). The combined organic layers were concentrated in a rotary evaporator under reduced pressure and crude product was purified through column chromatography with 10% EtOAc in *n*-hexane to afford (+)-**12** as white solid (6.8 g, 90% yield).

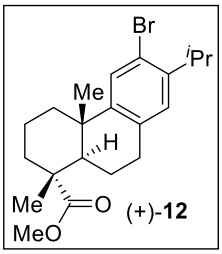

(1*R*,4a*S*,10a*R*)-**Methyl 6-bromo-7-isopropyl-1,4a-dimethyl-1,2,3,4,4a,9,10,10a-octahydrophenanthrene-1-carboxylate** [(+)-**12**]: (+)-**12** was obtained as white solid (19.1 mmol scale of reaction; 6.8 g; 90%). R_f = 0.6 (5% EtOAc in *n*-hexane).

^{1}H NMR (500 MHz, $CDCl_3$) δ 7.39 (s, 1H), 6.94 (s, 1H), 3.69 (s, 3H), 3.29 (s, 1H), 3.01 – 2.72 (m, 2H), 2.27 (dd, *J* = 12.4, 3.4 Hz, 1H), 2.20 (dd, *J* = 12.5, 2.3 Hz, 1H), 1.94 – 1.64 (m, 6H), 1.56 – 1.41 (m, 1H), 1.29 (s, 3H), 1.27 – 1.17 (m, 9H).

^{13}C NMR (125 MHz, $CDCl_3$) δ 178.9, 148.9, 144.0, 134.5, 128.5, 127.1, 121.5, 51.9, 47.6, 44.6, 37.9, 37.0, 36.6, 32.3, 29.5, 25.0, 23.0, 22.8, 21.5, 18.5,16.5.

IR (neat) υ_{max} 3015, 1842, 1798, 1641, 1495, 1381, 1332, 1201, 1105, 978, 703 cm^{-1}.
HRMS (ESI) *m/z*: $[M + H]^+$ calcd. for $[C_{21}H_{29}BrO_2 + H]^+$ 393.1398, found 393.1424.

$[\alpha]^{25}_{589}$ = +28.9 (*c* = 0.85, $CHCl_3$).

Preparation of Fuming Nitric Acid:
In an oven-dried round-bottom flask 40 g of powder potassium nitrate was charged with 60 mL of conc. sulfuric acid. Then the solution was set with a distillation condenser and distillation was performed at 130 ˚C to collect around 30 mL of fuming nitric acid.

***Ipso*-nitration of** (+)-**12**:

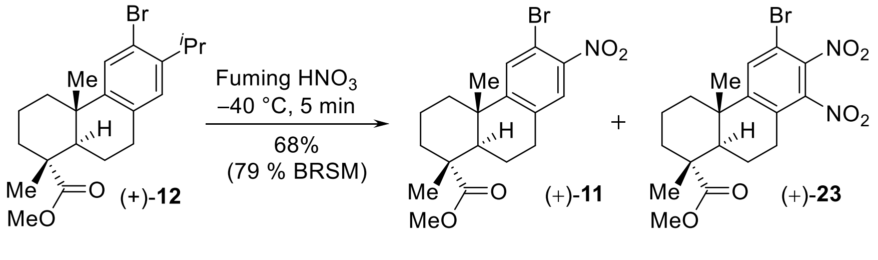

In an oven-dried round-bottom flask 4 mL of fuming nitric acid was taken and set at – 40 °C. Then solid compound (+)-**12** (800 mg, 2.03 mmol, 1.0 equiv.) was directly charged into the previously cooled fuming nitric acid system and the whole solution was scratched well with a spatula maintaining the –40 °C temperature. After scratching the solution for 5 minutes, the reaction was quenched with excess of water. The reaction mixture was then partitioned

between water and dichloromethane. The organic layer was then washed with saturated bicarbonate solution. The organic layer was dried over Na_2SO_4 and concentrated under reduced pressure. The crude product was then purified by column chromatography with 5% EtOAc in *n*-hexane to afford (+)-**11** as yellow foam [635 mg, 79% yield (brsm)].

**Sl. No**	**Condition**	**Solvent**	**Result**
1	KNO_3/Conc. H_2SO_4, 0 °C - 25°C, 5 h	CH_2Cl_2	30% (**11**) + 20% (**23**) + 20% SM
2	KNO_3/Conc. H_2SO_4, 0 °C - 25°C, 8 h	CH_2Cl_2	24% (**11**) + 30% (**23**)
3	KNO_3/Conc. H_2SO_4, –40°C, 10 min	-	46% (**11**) + 15% (**23**) + 39% SM
4	KNO_3/Conc. H_2SO_4, –40°C, 5 min	-	68% (**11**) + 18% SM

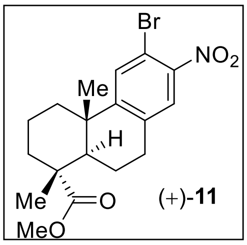

(1*R*,4a*S*,10a*R*)-**Methyl 6-bromo-1,4a-dimethyl-7-nitro-1,2,3,4,4a,9,10,10a octahydrophenanthrene-1-carboxylate** [(+)-**11**]: (+)-**11** was obtained as yellow foam [2.03 mmol scale of reaction; 635 g; 79% (BRSM)]. R_f = 0.35 (10% EtOAc in *n*-hexane).

^{1}H NMR (500 MHz, $CDCl_3$) δ 7.60 (s, 1H), 7.58 (s, 1H), 3.71 (s, 3H), 2.94 – 2.90 (m, 2H), 2.29 (d, *J* = 13.5 Hz, 1H), 2.19 (d, *J* = 12.5 Hz, 1H), 1.85 (s, 1H), 1.79 (s, 2H), 1.72 (s, 2H), 1.52 (t, *J* = 9.0 Hz, 2H).

^{13}C NMR (125 MHz, $CDCl_3$) δ 178.5, 155.9, 136.3, 131.3, 126.4, 111.2, 52.2, 47.4, 44.0, 37.8, 37.6, 36.4, 29.2, 24.8, 21.0, 18.3, 16.5.

IR (film) υ_{max} 2932, 1721, 1527, 1450, 1365, 1248, 1112, 981, 883, 734 cm^{-1}.
HRMS (ESI) *m/z*: $[M + Na]^+$ calcd. for $[C_{18}H_{22}BrNO_4 + Na]^+$ 418.0624, found 418.0575.

$[\alpha]^{25}_{589}$ = +89.75 (*c* = 0.62, $CHCl_3$).

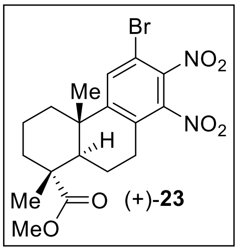

(1*R*,4a*S*,10a*R*)-**Methyl 6-bromo-1,4a-dimethyl-7,8-dinitro-1,2,3,4,4a,9,10,10a-octahydrophenanthrene-1-carboxylate** [(+)-**23**]: (+)-**23** was obtained as yellow foam (2.03 mmol scale of reaction; 161 mg; 18%). R_f = 0.30 (10% EtOAc in hexane).

^{1}H NMR (500 MHz, $CDCl_3$) δ 7.74 (s, 1H), 3.72 (s, 3H), 2.95 – 2.81 (m, 2H), 2.34 – 2.29 (m, 1H), 2.21 (dd, *J* = 12.6, 2.3 Hz, 1H), 1.85 – 1.79 (m, 4H), 1.75 (dd, *J* = 8.5, 5.9 Hz, 2H), 1.52 (d, *J* = 4.5 Hz, 1H), 1.31 (s, 3H), 1.27 (s, 3H).

^{13}C NMR (125 MHz, $CDCl_3$) δ 178.0, 156.3, 133.0, 132.4, 129.2, 127.0, 112.0, 52.3, 47.2, 43.2, 38.4, 37.8, 36.2, 25.1, 24.9, 20.0, 18.2, 16.5.

IR (neat) υ_{max} 2945, 1720, 1536, 1485, 1320, 1265, 1156, 986, 912, 750 cm^{-1}.
HRMS (ESI) *m/z*: $[M + H]^+$ calcd. for $[C_{18}H_{21}BrN_2O_6 + H]^+$ 441.0674; found 441.0656.

$[\alpha]^{25}_{589}$ = +78.35 (*c* = 0.72, $CHCl_3$).

Suzuki-Miyaura Coupling of (+)-**11 with Phenylboronic acid**:

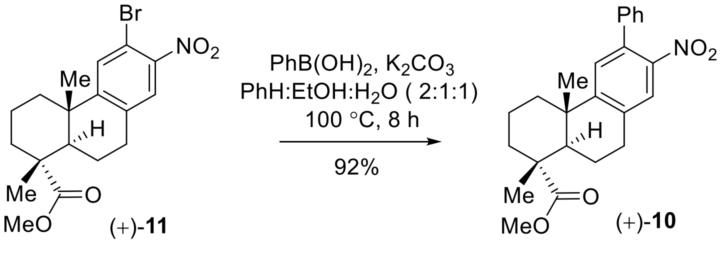

In an oven-dried round-bottom flask, compound (+)-**11** (2.1 g, 5.3 mmol, 1 equiv.) was taken in 20 mL mixed solvent system of benzene: ethanol: water (2:1:1) equipped with a magnetic

stir-bar. Then benzene boronic acid (775 mg, 6.36 mmol, 1.2 equiv.) and potassium carbonate (1.5 g, 10.6 mmol, 2 equiv.) were directly added to the reaction mixture. After the complete dissolution of the solid materials the reaction mixture was degassed for 10 mins using N_2 gas balloon. Then tetrakis(triphenylphosphine)palladium(0) (123 mg, 0.11 mmol, 0.02 equiv.) was rapidly added and the reaction mixture was allowed to reflux at 100 °C on a preheated oil-bath for 8 h maintaining N_2 inertness until the full consumption of starting material (monitored by TLC). The mixture was cooled and was poured into an aqueous ammonium chloride solution. The mixture was extracted with 20% EtOAc in *n*-hexane (25 mL X 2). The combined organic layers were washed with brine (20 mL X 1), dried over Na_2SO_4 and concentrated in a rotary evaporator under reduced pressure. Now the crude product was purified by flash chromatography with 5% EtOAc in *n*-hexane to afford (+)-**10** as yellow foam (1.92 g, 92% yield).

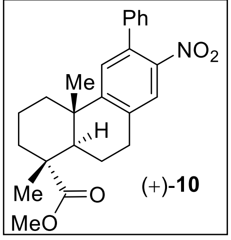

(+)-**10**

(1*R*,4a*S*,10a*R*)-**Methyl 1,4a-dimethyl-7-nitro-6-phenyl-1,2,3,4,4a,9,10,10a-octahydrophenanthrene-1-carboxylate** [(+)-**10**]**:** (+)-**10** was obtained as yellow foam (5.3 mmol scale of reaction; 1.9 g; 92%). R_f = 0.35 (10% EtOAc in *n*-hexane).

^{1}H NMR (500 MHz, $CDCl_3$) 7.56 (s, 1H), 7.37 (p, *J* = 7.7, 7.2 Hz, 4H), 7.28 – 7.25 (m, 2H), 3.68 (s, 3H), 2.98 (dd, *J* = 8.2, 3.9 Hz, 2H), 2.29 (d, *J* = 11.1 Hz, 1H), 2.23 (dd, *J* = 12.3, 2.3 Hz, 1H), 1.87 (td, *J* = 8.8, 4.4 Hz, 1H), 1.77 – 1.73 (m, 3H), 1.69 – 1.67 (m, 1H), 1.54 – 1.49 (m, 2H), 1.28 (s, 3H), 1.23 (s, 3H).

^{13}C NMR (125 MHz, $CDCl_3$) δ 178.7, 154.4, 146.6, 138.2, 135.9, 133.9, 128.5, 128.2, 128.0, 127.8, 124.7, 52.1, 47.5, 44.3, 37.8, 36.5, 31.6, 29.4, 24.9, 21.2, 18.3, 16.6.

IR (film) υ_{max} 3015, 1842,1798, 1641, 1495, 1381, 1332, 1201, 1105, 978, 703 cm^{-1}.
HRMS (ESI) *m/z*: $[M + H]^+$ calcd. for $[C_{24}H_{27}NO_4 + H]^+$ 394.2013, found 394.2016.

$[\alpha]^{25}_{589}$ = +62.50 (*c* = 0.79, $CHCl_3$).

Cadogan Reaction of (+)-**10**:

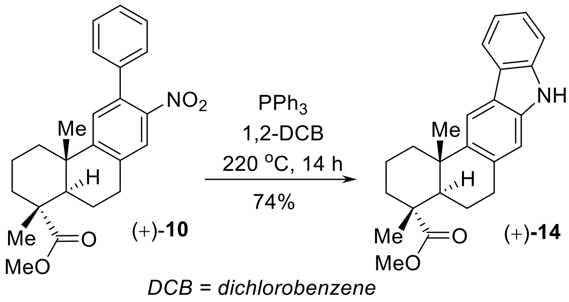

In an oven-dried round-bottom flask compound (+)-**10** (1.72 g, 4.4 mmol, 1.0 equiv.) was taken in 10 mL of 1, 2-dichlorobenzene maintaining N_2 inertness. Then to the reaction mixture solid triphenyl phosphine (3.43 g, 13.1 mmol, 3.0 equiv.) was added and refluxed at 220 °C on a preheated oil-bath for 14 h until the full consumption of starting material (monitored by TLC). Now the crude product was purified by flash chromatography with 10% EtOAc in *n*-hexane to afford (+)-**14** as brown foam (1.2 g, 74% yield).

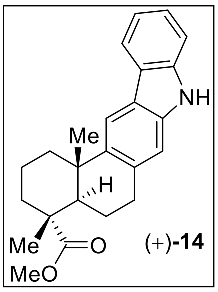

(4*R*,4a*R*,13b*S*)-**Methyl 4,13b-dimethyl-2,3,4,4a,5,6,8,13b-octahydro-1H-naphtho[2,1-*b*]carbazole-4-carboxylate** [(+)-**14**]: (+)-**14** was obtained as brown foam (4.4 mmol scale of reaction; 1.2 g; 74%). R_f = 0.35 (20% EtOAc in *n*-hexane).

^{1}**H NMR** (500 MHz, $CDCl_3$) δ 8.05 (d, *J* = 7.8 Hz, 1H), 7.98 (s, 1H), 7.85 (s, 1H), 7.41 – 7.35 (m, 2H), 7.22 (ddd, *J* = 7.7, 5.8, 2.0 Hz, 1H), 7.07 (d, *J* = 2.9 Hz, 1H), 3.72 (d, *J* = 1.3 Hz, 3H), 3.15 – 3.07 (m, 2H), 2.58 (dd, *J* = 12.0, 3.2 Hz, 1H), 2.40 – 2.35 (m, 1H), 2.02 – 1.94 (m, 1H), 1.88 – 1.82 (m, 2H), 1.75 – 1.68 (m, 2H), 1.65 – 1.61 (m, 1H), 1.52 (ddd, *J* = 13.4, 6.2, 3.2 Hz, 1H), 1.37 (d, *J* = 1.3 Hz, 3H), 1.34 (s, 3H).

^{13}**C NMR** (125 MHz, $CDCl_3$) δ 179.3, 141.9, 140.0, 138.1, 133.7, 125.3, 123.7, 121.9, 119.9, 119.1, 115.4, 110.4, 109.9, 52.0, 47.7, 45.2, 38.8, 37.5, 36.8, 30.7, 25.7, 21.9, 18.7, 16.6.

IR (neat) ν_{max}3402, 2926, 1720, 1465, 1243, 1023, 823, 750, 582 cm^{-1}.

HRMS (ESI) *m/z*: [M+ H]$^+$ calcd. for $[C_{24}H_{27}NO_2 + H]^+$ 362.2115, found 362.2113.

$[\alpha]^{25}_{589}$ = +122.0 (*c* = 0.93, $CHCl_3$).

Tosylation of Carbazole derivative (+)-**14**:

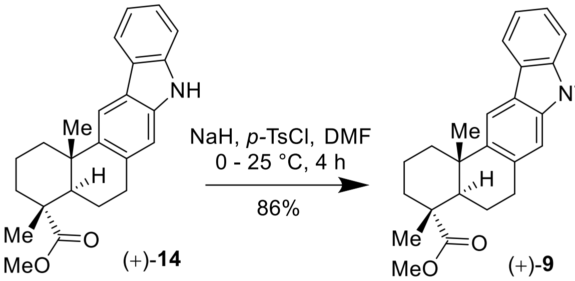

Carbazole (+)-**14** (1.1 g, 3.04 mmol, 1.0 equiv.) was taken in an oven dried round bottom flask dissolved in 12 mL of DMF maintaining N_2 inertness and set on an ice bath. Sodium hydride (182 mg, 4.56 mmol, 1.5 equiv.) was added in portion-wise manner to the reaction vessel and stirred for 15 min at 0 °C. Then solid *p*-toluene sulphonyl chloride (695 mg, 3.65 mmol, 1.2 equiv.) was directly added to the solution and the reaction mixture was allowed to stir at 25 ^{o}C for 4 h until the full consumption of starting material (monitored by TLC). The reaction was quenched with excess of saturated aqueous NH_4Cl solution. Then the solution was extracted with EtOAc and water. The aqueous phase was extracted with EtOAc (20 mL X 3). The combined organic layers were dried over anhydrous Na_2SO_4 and concentrated in a rotary evaporator under vacuum. The crude product was purified by flash chromatography with 10% EtOAc in *n*-hexane to afford (+)-**9** as yellow foam (1.35 g, 86% yield).

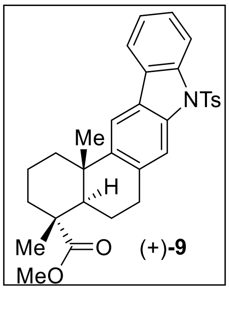

(4*R*,4a*R*,13b*S*)-**Methyl 4,13b-dimethyl-8-tosyl-2,3,4,4a,5,6,8,13b-octahydro-1H-naphtho[2,1-*b*]carbazole-4-carboxylate** [(+)-**9**]: (+)-**9** was obtained as yellow foam (3.04 mmol scale of reaction; 1.35 g; 86%). R_f = 0.35 (20% EtOAc in *n*-hexane).

^{1}H NMR (500 MHz, $CDCl_3$) δ 8.28 (d, *J* = 8.4 Hz, 1H), 7.98 (s, 1H), 7.86 (d, *J* = 7.7 Hz, 1H), 7.78 (s, 1H), 7.72 (d, *J* = 8.1 Hz, 2H), 7.44 (s, 1H), 7.34 (d, *J* = 7.6 Hz, 1H), 7.14 (d, *J* = 8.1 Hz, 2H), 3.73 (s, 3H), 3.16 (dd, *J* = 7.4, 3.0 Hz, 2H), 2.47 (d, *J* = 12.5 Hz, 1H), 2.30 (s, 3H), 1.98 – 1.93 (m, 1H), 1.85 – 1.76 (m, 4H), 1.71 (d, *J* = 9.0 Hz, 1H), 1.62 (d, *J* = 8.4 Hz, 1H), 1.56 – 1.52 (m, 1H), 1.34 (s, 3H), 1.29 (s, 3H).

^{13}C NMR (125 MHz, $CDCl_3$) δ 179.1, 146.0, 144.7, 138.5, 136.6, 135.4, 135.2, 129.7, 129.6, 126.8, 126.7, 126.5, 126.5, 124.4, 123.7, 119.5, 115.2, 115.0, 114.7, 52.0, 47.6, 44.8, 38.4, 37.5, 36.7, 30.8, 25.5, 21.7, 21.5, 18.6, 16.6.

IR (neat) ν_{max} 2921, 1721, 1598, 1368, 1171, 995, 810,747, 668, 581 cm^{-1}.
HRMS (ESI) *m/z*: $[M+ Na]^+$ calcd. for $[C_{31}H_{33}NO_4S + Na]^+$ 538.2023, found 538.2041.

$[\alpha]^{25}_{589}$ = +139.23 (*c* = 0.82, $CHCl_3$).

Benzylic Oxidation of Compound (+)-**9:**

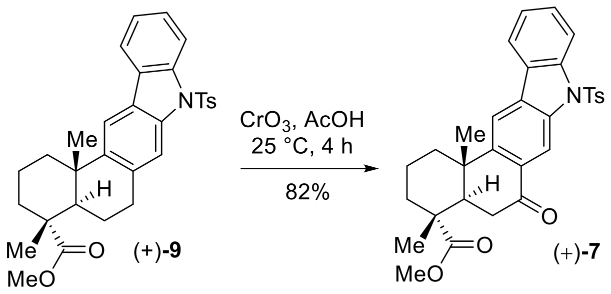

In an oven-dried round-bottom flask (+)-**9** (960 mg, 1.86 mmol, 1.0 equiv.) was dissolved in 10 mL of acetic acid. To the reaction mixture solid CrO_3 (372 mg, 3.72 mmol, 2.0 equiv.) was added at 25 ºC and stirred for 4 h. After completion of the reaction (monitored by TLC) the reaction mixture was diluted with ethyl acetate and quenched with saturated aqueous sodium bicarbonate solution. The mixture was extracted with EtOAc (15 mL X 3). The combined organic layers were washed with brine and dried over Na_2SO_4 and concentrated under reduced pressure. Then the crude product was purified by flash chromatography with 20% EtOAc in *n*-hexane to afford (+)-**7** as white foam (808 mg, 82% yield).

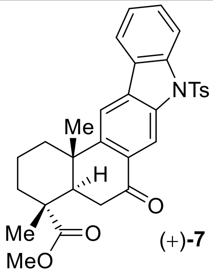

(+)-**7**

Methyl (4*R*,4a*R*,13b*S*)-**4,13b-dimethyl-6-oxo-8-tosyl-2,3,4,4a,5,6,8,13b-octahydro-1H-naphtho[2,1-*b*]carbazole-4-carboxylate** [(+)-**7**]: (+)-**7** was obtained as white foam (1.86 mmol scale of reaction; 808 mg; 82%). R_f = 0.4 (30% EtOAc in *n*-hexane).

^{1}H NMR (500 MHz, $CDCl_3$) δ 8.93 (s, 1H), 8.38 (d, *J* = 8.4 Hz, 1H), 7.98 (d, *J* = 7.7 Hz, 1H), 7.90 (s, 1H), 7.78 (d, *J* = 8.0 Hz, 2H), 7.61 – 7.56 (m, 1H), 7.41 (t, *J* = 7.5 Hz, 1H), 7.16 (d, *J* = 8.1 Hz, 2H), 3.71 (s, 3H), 2.88 – 2.76 (m, 2H), 2.53 (d, *J* = 12.1 Hz, 1H), 2.48 (d, *J* = 16.0 Hz, 1H), 2.30 (s, 3H), 1.90 – 1.82 (m, 3H), 1.81 – 1.75 (m, 2H), 1.40 (s, 3H), 1.33 (s, 3H).

^{13}C NMR (125 MHz, $CDCl_3$) δ 197.5, 177.9, 151.1, 145.0, 140.0, 136.5, 135.0, 130.8, 130.3, 129.8, 128.9, 126.7, 125.4, 124.0, 120.8, 115.2, 114.6, 114.1, 52.3, 46.7, 43.7, 37.9, 37.7, 37.6, 36.6, 24.3, 21.5, 18.2, 16.5.

IR (film) υ_{max} 2942, 1746, 1705, 1576, 1428, 1374, 1157, 654, 573 cm^{-1}.
HRMS (ESI) *m/z*: $[M+ H]^+$ calcd. for $[C_{31}H_{31}NO_5S + H]^+$ 530.1996, found 530.1999.

$[\alpha]^{25}_{589}$ = +64.0 (*c* = 0.92, $CHCl_3$).

Stereoselective reduction of (+)-**7:**

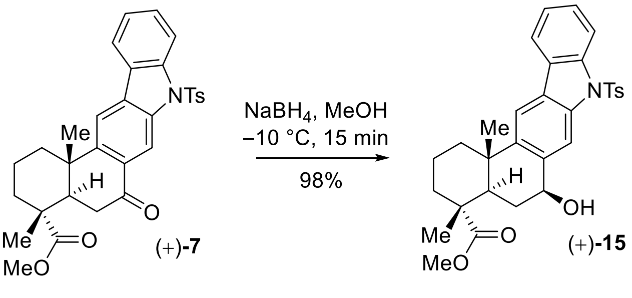

In an oven-dried round-bottom flask (+)-**7** (100 mg, 0.189 mmol, 1.0 equiv.) in MeOH (4 mL) was taken at −10 °C, $NaBH_4$ (9 mg, 0.226 mmol, 1.2 equiv.) was added portion wise and the

reaction mixture was stirred at same temperature for 15 min. After complete consumption of starting material (monitored by TLC analysis), it was quenched with saturated NH_4Cl (5 mL) and extracted with EtOAc (5 mL X 2). The organic layers were dried over Na_2SO_4 and concentrated on a rotary evaporator under reduced pressure. The crude products were purified by flash chromatography with 40% EtOAc in *n*-hexane to afford (+)-**15** as yellow foam (98 mg, 98% yield).

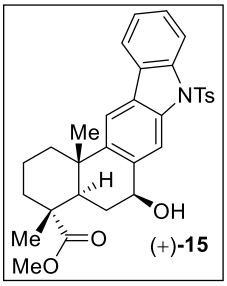

Methyl (4*R*,4a*R*,6*S*,13b*S*)-**6-hydroxy-4,13b-dimethyl-8-tosyl-2,3,4,4a,5,6,8,13b-octahydro-1H-naphtho[2,1-*b*]carbazole-4-carboxylate** [(+)-**15**]: (+)-**15** was obtained as yellow foam (0.189 mmol scale of reaction; 98 mg; 98%). R_f = 0.32 (30% EtOAc in *n*-hexane).

^{1}**H NMR** (500 MHz, $CDCl_3$) δ 8.45 (s, 1H), 8.31 (d, *J* = 8.4 Hz, 1H), 7.89 (d, *J* = 7.6 Hz, 1H), 7.77 (s, 1H), 7.75 (s, 1H), 7.74 (s, 1H), 7.48 (ddd, *J* = 8.5, 7.2, 1.3 Hz, 1H), 7.36 (t, *J* = 7.5 Hz, 1H), 7.13 (d, *J* = 8.1 Hz, 2H), 5.08 (t, *J* = 8.7 Hz, 1H), 3.74 (s, 3H), 2.48 – 2.44 (m, 1H), 2.36 (dd, *J* = 9.0, 5.5 Hz, 1H), 2.29 (s, 3H), 1.96 – 1.91 (m, 2H), 1.89 – 1.84 (m, 1H), 1.78 (dd, *J* = 13.7, 3.2 Hz, 2H), 1.75 – 1.71 (m, 1H), 1.56 (td, *J* = 12.8, 3.4 Hz, 1H), 1.37 (s, 3H), 1.36 (s, 3H).

^{13}**C NMR** (125 MHz, $CDCl_3$) δ 178.8, 145.7, 144.8, 138.9, 138.1, 136.9, 135.1, 129.7, 127.3, 126.59, 126.3, 125.8, 123.7, 119.8, 115.3, 115.1, 113.8, 70.9, 52.2, 47.3, 43.5, 38.4, 38.1, 36.5, 32.7, 26.0, 21.5, 18.5, 16.5.

IR (film) υ_{max} 3645, 3100, 2885, 1740, 1695, 1535, 1430, 1365, 1169 cm^{-1}.

HRMS (ESI) *m/z*: $[M + Na]^+$ calcd. for $[C_{31}H_{33}NO_5S + Na]^+$ 554.1972; found 554.1950.

$[\alpha]^{25}_{589}$ = +56.5 (*c* = 0.72, $CHCl_3$).

Synthesis of Enone (+)-**8 via Saegusa-Ito Oxidation**:

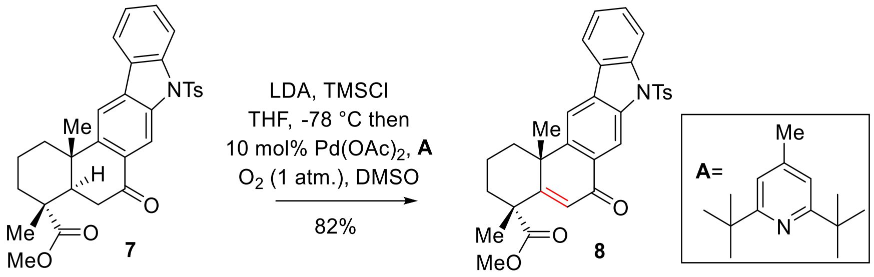

To a stirred solution of **7** (770 mg, 1.45 mmol, 1.0 equiv.) in THF (12 mL) at –78 ºC, a freshly prepared LDA (2.175 mmol, 1.5 equiv.) was added. To this solution was added TMSCl (927 μL, 7.25 mmol, 5 equiv.) dropwise over 5 min. After stirring at –78 ºC for 30 mins, the reaction mixture was quenched with triethylamine (3 mL) at -78 ºC followed by saturated aq. $NaHCO_3$ (10 mL). Then, it was allowed to warm to 25 ºC, diluted with water (6 mL) and extracted with Et_2O (15 mL X 3). The combined organic extracts were dried over K_2CO_3 and concentrated under vacuum. The crude product was used without further purification.

The crude silyl enol ether was dissolved in CH_3CN (12 mL), treated with 2,6-di-*tert*-butyl-4-methylpyridine (447 mg, 2.175 mmol, 1.5 equiv.) and $Pd(OAc)_2$ (65.3 mg, 0.29 mmol, 0.2 equiv.) and placed under an atmosphere of oxygen (1 atm. balloon). The dark suspension was stirred at 25 ºC for 16 h (until TLC indicated complete consumption of starting material) and diluted with Et_2O (10 mL) and H_2O (10 mL). The aqueous layer was extracted with Et_2O (15 mL X 2), and the combined organic layers were washed with saturated NaCl (5 mL), dried over $MgSO_4$, and concentrated under vacuum. The crude product was purified by flash chromatography to provide (+)-**8** in 627 mg (82% yield) as yellow semi-solid.

Synthesis of Enone (+)-**8 by reaction with SeO_2 in AcOH and water**:

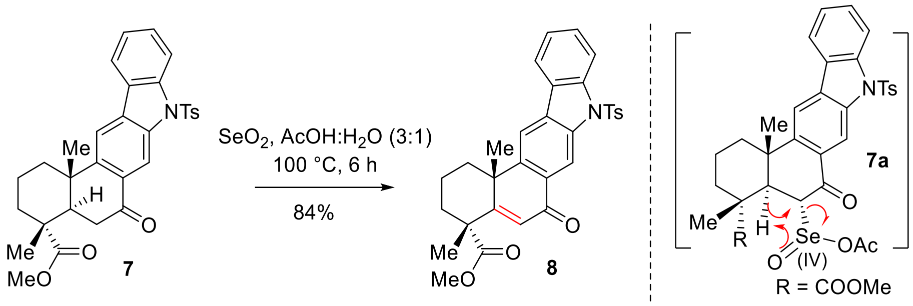

A mixture of (+)-**7** (770 mg, 1.45 mmol, 1.0 equiv.) and selenium dioxide (804 mg, 7.27 mmol, 5.0 equiv.) were dissolved in 12 mL solvent mixture of acetic acid and water (3:1). The reaction mixture was refluxed for 6 h on a preheated oil-bath at 100 ºC. After complete consumption of starting material (monitored by TLC) the reaction mixture was cooled to room temperature and diluted with ethyl acetate and quenched with saturated aqueous sodium bicarbonate solution. The reaction mixture was extracted with EtOAc (10 mL X 3). The combined organic layers were dried over anhydrous Na_2SO_4 and concentrated in a rotary evaporator under reduced pressure. The residue was purified by flash chromatography with 20% EtOAc in *n*-hexane to afford (+)-**8** as yellow gel (643 mg, 84% yield).

Synthesis of Enone (+)-**8 by reaction with PTAB followed by DBU**:

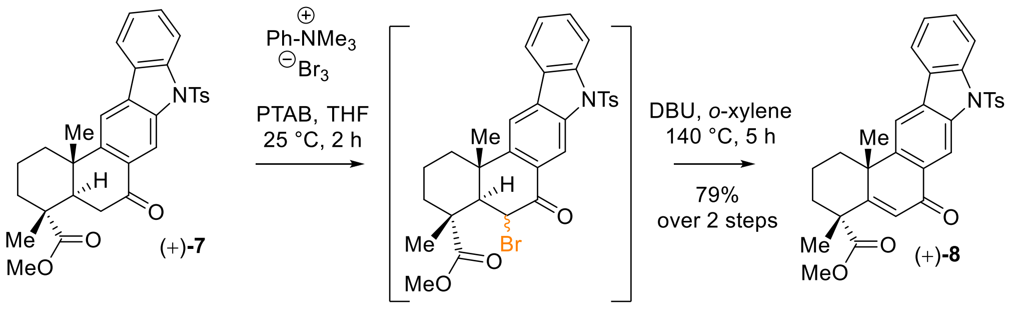

To a solution of the ketone (−)-**7** (636 mg, 1.2 mmol, 1.0 equiv.) in 12 mL of THF was added trimethyl(phenyl)ammonium perbromide (PTAB, 542 mg, 1.44 mmol, 1.2 equiv.) at 0 °C. After stirred for 1.5 h, the reaction mixture was quenched with saturated $Na_2S_2O_3$ solution and extracted with ethyl acetate (15 mL). The combined organic layers were washed with brine, dried over Na_2SO_4, concentrated in vacuo. Then the residue was dissolved *o*-xylene (12 mL) was added DBU (366 µL, 2.4 mmol, 2.0 equiv.) at room temperature. The resulting reaction mixture was stirred at 140 °C for 5 h, diluted with water (12 mL), and extracted with ethyl acetate (15 mL X 2). The combined organic extracts were washed with 4 (*N*) HC1 aqueous solution and brine, dried over anhydrous Na_2SO_4, and concentrated in vacuum. The crude products were purified by flash chromatography to afford (+)-**8** as yellow gel (501 mg, 79% yield).

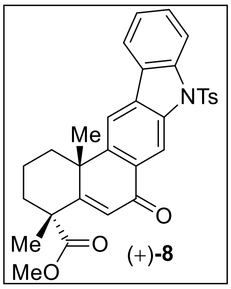
(+)-**8**

Methyl (4*R*,13b*R*)-4,13b-dimethyl-6-oxo-8-tosyl-2,3,4,6,8,13b-hexahydro-1H-naphtho[2,1-*b*]carbazole-4-carboxylate [(+)-**8**]: (+)-**8** was obtained as yellow gel (1.45 mmol scale of reaction; 643 mg; 84%). R_f = 0.35 (30% EtOAc in *n*-hexane);
^{1}H NMR (400 MHz, $CDCl_3$) δ 8.99 (s, 1H), 8.36 (d, *J* = 8.5 Hz, 1H), 8.01 (s, 1H), 7.95 (d, *J* = 7.7 Hz, 1H), 7.77 (d, *J* = 8.1 Hz, 2H), 7.58 – 7.53 (m, 1H), 7.38 (t, *J* = 7.5 Hz, 1H), 7.11 (d, *J* = 8.0 Hz, 2H), 6.23 (s, 1H), 3.73 (s, 3H), 2.64 (d, *J* = 12.7 Hz, 1H), 2.26 (s, 3H), 2.21 (d, *J* = 5.2 Hz, 1H), 2.08 – 2.01 (m, 1H), 2.00 – 1.95 (m, 1H), 1.90 (dd, *J* = 14.6, 2.5 Hz, 1H), 1.85 (d, *J* = 4.4 Hz, 1H), 1.66 (s, 3H), 1.57 (s, 3H).

^{13}C NMR (125 MHz, $CDCl_3$) δ 184.5, 176.2, 167.2, 148.5, 145.0, 140.0, 136.8, 135.0, 130.0, 129.8, 129.8, 128.9, 126.7, 125.3, 124.0, 120.7, 115.6, 115.2, 112.8, 52.6, 50.8, 41.1, 35.7, 35.0, 34.5, 25.3, 21.5, 17.7.

IR (neat) υ_{max} 3050, 2958, 1763, 1656, 1554, 1162, 1395, 678 cm^{-1}.

HRMS (ESI) *m/z*: $[M+H]^+$ calcd. for $[C_{31}H_{29}NO_5S + H]^+$ 528.1839, found 528.1863.
$[\alpha]^{25}_{589}$ = +60.48 (*c* = 0.75, $CHCl_3$).

General procedure for δ-Csp^3-H Activation: The substrate was taken in 2 mL solvent (cyclohexane or DCE) (1.0 equiv.) and to it PIDA was added, followed by addition of oxidant (I_2 or NBS) was done. The reaction was irradiated with a 200-W tungsten lamp for 30-50 min. at 40 °C. After completion of starting material (monitored by TLC) the reaction mixture was cooled to room temperature and diluted with ethyl acetate and quenched with saturated aqueous $Na_2S_2O_3$ solution. The reaction mixture was extracted with EtOAc The combined organic layers were dried over anhydrous Na_2SO_4 and concentrated in a rotary evaporator under reduced pressure. The residue was purified by flash chromatography with 20-40% EtOAc in *n*-Hexane to afford the products (see the optimization table).

Optimization of oxidation using hypervalent iodine reagent:

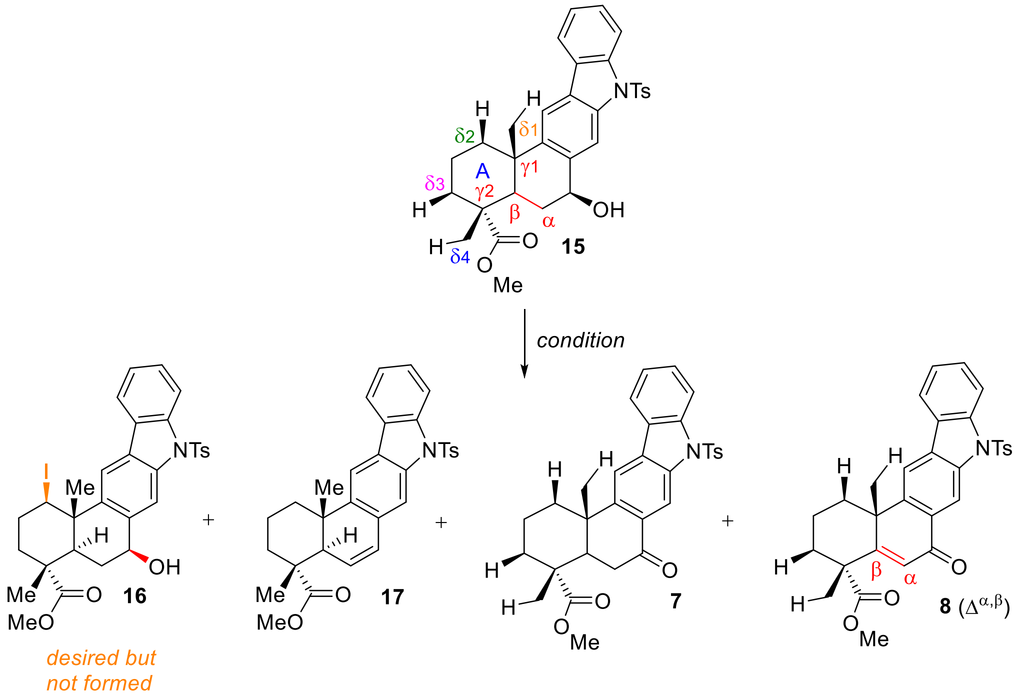

Entry	Conditions[a]	temp.	**17**[b]	**7**[b]	**8**[b]	**16**[b]
1	PIDA (1.1 equiv.) I_2 (1.0 equiv.), cyclohexane, 40 min	25 °C	27%	58%	-	*ND*
2	PIDA (2.0 equiv.) I_2 (2.0 equiv.) DCE, 40 min	25 °C	21%	45%	-	*ND* + 21% SM recovered
3	PIDA (4.0 equiv.) I_2 (5.0 equiv.), DCE, 40 min	25 °C	19%	37%	3%	*ND* + multiple spots+ 15% SM recovered
4	PIDA (4.0 equiv.) I_2 (5.0 equiv.), DCE, 40 min	40 °C	35%	52%	6%	*ND*
5	PIDA (1.1 equiv.) NBS (1.0 equiv.), cyclohexane, 40 min	25 °C	12%	23%	-	*ND* + 47% SM recovered
6	PIDA (2.0 equiv.) NBS (2.0 equiv.) DCE, 40 min	25 °C	18%	38%	-	*ND* + 31% SM recovered
7	PIDA (4.0 equiv.) NBS (5.0 equiv.) DCE, 40 min	40 °C	22%	43%	13%	*ND* + multiple spots +10% SM recovered
8	PIFA (2.0 equiv.) I_2 (2.0 equiv.) DCE, 40 min	25 °C	11%	26%	-	*ND* + multiple spots+ 41% SM recovered

[a]Reactions were carried out on 0.037mmol of substrate. [b]yields are isolated after column chromatography.

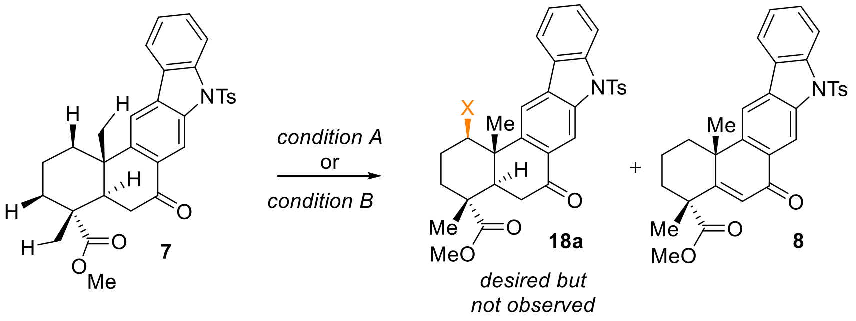

		8	18a
Condition A	PIDA (4.0 equiv.) I_2 (5.0 equiv.), hv 40 °C, DCE, 1 h	38%	ND + multiple spots + 35% SM **7** recovered
Condition B	PIDA (4.0 equiv.) NBS (5.0 equiv.), hv 25 °C, DCE, 1 h	17%	ND + 45% SM **7** recovered

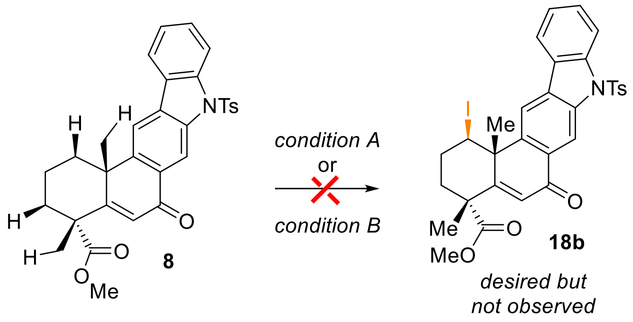

		18b
Condition A	PIDA (4.0 equiv.) I_2 (5.0 equiv.), hv 40 °C, DCE, 1 h	*ND* + multiple spots + 57% SM **8** recovered
Condition B	PIDA (4.0 equiv.) NBS (5.0 equiv.), hv 25 °C, DCE, 1 h	*ND* + 49% SM **8** recovered

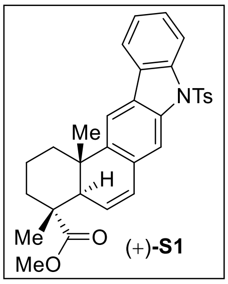

Methyl (4*R*,4a*R*,13b*S*)-**4,13b-dimethyl-8-tosyl-2,3,4,4a,8,13b-hexahydro-1H-naphtho[2,1-b]carbazole-4-carboxylate** [(+)-**S1**]: (+)-**S1** was obtained as colorless oil (0.037 mmol scale of reaction; 5 mg; 27%). R_f = 0.65 (30% EtOAc in *n*-hexane);

^{1}H NMR (500 MHz, $CDCl_3$) δ 8.30 (d, *J* = 8.5 Hz, 1H), 8.02 (s, 1H), 7.89 (d, *J* = 7.7 Hz, 1H), 7.74 (s, 1H), 7.72 (s, 1H), 7.69 (s, 1H), 7.48 – 7.44 (m, 1H), 7.35 (t, *J* = 7.7 Hz, 1H), 7.14 (d, *J* = 8.2 Hz, 2H), 6.76 (dd, *J* = 9.6, 3.1 Hz, 1H), 5.87 (dd, *J* = 9.6, 2.7 Hz, 1H), 3.71 (s, 3H), 2.99 (t, *J* = 3.0 Hz, 1H), 2.30 (s, 3H), 1.87 (s, 3H), 1.86 – 1.81 (m, 2H), 1.79 – 1.75 (m, 1H), 1.47 (s, 3H), 1.16 (s, 3H).

^{13}C NMR (125 MHz, $CDCl_3$) δ 178.5, 144.8, 144.3, 138.6, 136.8, 135.1, 132.8, 130.7, 129.7, 128.8, 126.9, 126.7, 126.5, 125.4, 123.8, 119.6, 115.0, 113.2, 113.0, 52.2, 46.6, 46.3, 37.8, 35.7, 35.6, 21.5, 21.3, 18.4, 18.0.

IR (neat) υ_{max} 3070, 2900, 1725, 1680, 1510, 1475, 1120 cm^{-1}.

HRMS (ESI) *m*/*z*: $[M + H]^+$ calcd. for $[C_{31}H_{31}NO_4S + H]^+$ 514.2047; found 514.2019.

$[\alpha]^{25}_{589}$ = +78.64 (*c* = 0.38, $CHCl_3$).

Procedure for the Synthesis of Compound (–)-19:

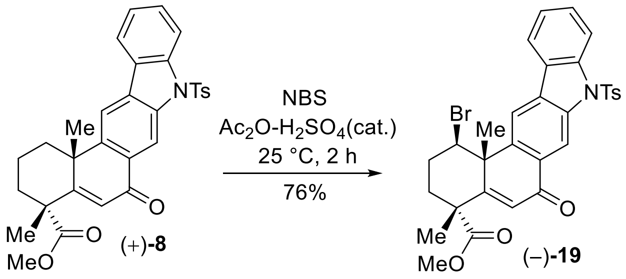

(+)-**8** (–)-**19**

In an oven-dried round-bottom flask (+)-**8** (610 mg, 1.16 mmol, 1.0 equiv.) was taken in 8 mL of Ac_2O and catalytic H_2SO_4 was added at 25 ºC. After 5 minutes solid NBS (227 mg, 1.3 mmol, 1.1 equiv.) was added to reaction mixture and stirred for 2 h at 25 ºC. After completion of the reaction (monitored by TLC), the reaction mixture was quenched with saturated aqueous $Na_2S_2O_3$ solution. The reaction mixture was then partitioned between water and EtOAc (10 mL X 2). The organic layer was washed with saturated $NaHCO_3$ solution (10 mL X 1) and dried over Na_2SO_4 and concentrated under reduced pressure. The crude product was purified by flash column chromatography with 16% EtOAc in *n*-hexane to afford (–)-**19** as yellow gel (535 mg, 76% yield).

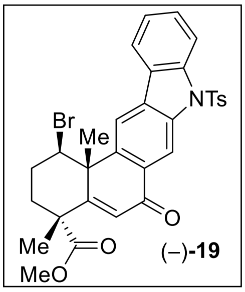

Methyl (1*R*,4*R*,13b*R*)**-1-bromo-4,13b-dimethyl-6-oxo-8-tosyl-2,3,4,6,8,13b-hexahydro-1H-naphtho[2,1-*b*]carbazole-4-carboxylate** [(–)-**19**]: (–)-**19** was obtained as yellow gel (1.16 mmol scale of reaction; 535 mg; 76%). R_f = 0.37 (30 % EtOAc in *n*-hexane).

^{1}H NMR (500 MHz, $CDCl_3$) δ 9.01 (s, 1H), 8.82 (s, 1H), 8.39 (d, *J* = 8.4 Hz, 1H), 8.05 (d, *J* = 7.7 Hz, 1H), 7.80 (d, *J* = 8.2 Hz, 2H), 7.64 – 7.57 (m, 1H), 7.44 (t, *J* = 7.5 Hz, 1H), 7.15 (d, *J* = 8.1 Hz, 2H), 6.41 (s, 1H), 4.98 (t, *J* = 4.7 Hz, 1H), 3.78 (s, 3H), 2.82 – 2.71 (m, 1H), 2.45 (ddt, *J* = 15.7, 6.7, 3.4 Hz, 1H), 2.30 (s, 3H), 2.18 – 2.07 (m, 2H), 1.91 (s, 3H), 1.73 (s, 3H).

^{13}C NMR (125 MHz, $CDCl_3$) δ 183.4, 175.5, 164.3, 147.5, 145.1, 140.0, 137.1, 134.9, 130.4, 129.8, 129.1, 129.1, 128.5, 126.7, 125.3, 124.1, 121.0, 117.2, 115.2, 112.5, 62.2, 52.8, 50.6, 44.9, 37.0, 34.6, 30.4, 27.2, 21.5.

IR (neat) υ_{max} 3041, 2967, 1758, 1674, 1571, 1431, 1168, 629 cm^{-1}.
HRMS (ESI) *m/z*: $[M+ Na]^+$ calcd. for $[C_{31}H_{28}NBrO_5S + Na]^+$: 628.0764, found: 628.0740.

$[\alpha]^{25}_{589}$ = –6.19 (*c* = 0.73, $CHCl_3$).

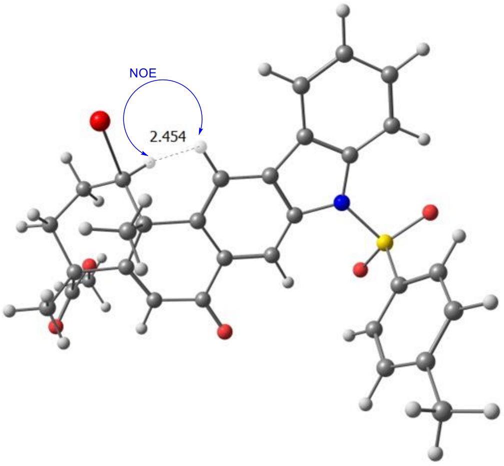

Energy minimization was done with DFT method using the B3LYP functional using LANL2DZ basis set.

Overview Tab Data Section:

AB-RN

C:/Users/BISHNU/Desktop/AB-RN.log

File Type = .log

Calculation Type = FOPT

Calculation Method = RB3LYP

Basis Set = LANL2DZ

Charge = 0

Spin = Singlet

Solvation = None

E(RB3LYP) = -1651.8781 Hartree

RMS Gradient Norm = 2.123e-06 Hartree/Bohr

Dipole Moment = 6.1157955 Debye

Point Group = C1

Job cpu time: 1 days 7 hours 49 minutes 20.3 seconds.

Opt Tab Data Section:

Step number = 59

Maximum force = 9e-06 Converged

RMS force = 1e-06 Converged

Maximum displacement = 0.001581 Converged

RMS displacement = 0.000208 Converged

Predicted energy change = -2.149296e-09 Hartree

Synthesis of Enol-acetate (–)-8c:

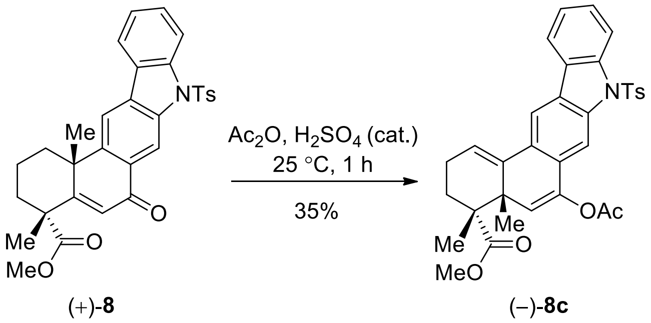

In an oven-dried round-bottom flask compound (+)-**8** (100 mg, 0.19 mmol, 1.0 equiv.) was

taken in 2 mL of Ac_2O and catalytic H_2SO_4 was added at 25 ºC. After that reaction mixture was run for 1 h at 25 ºC. After completion of the reaction (monitored by TLC), the reaction mixture was diluted with EtOAc (5 mL). The organic layer was quenched with saturated $NaHCO_3$ solution (5 mL X 1). Then reaction mixture was partitioned between water and EtOAc (5 mL X 2) and organic layer was dried over Na_2SO_4 and concentrated under reduced pressure. The crude product was purified by flash column chromatography with 20% EtOAc in *n*-hexane to afford (–)-**8c** as yellow foam (38 mg, 35% yield).

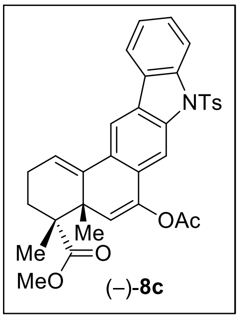

(4*R*,4a*S*)**-Methyl 6-acetoxy-4,4a-dimethyl-8-tosyl-3,4,4a,8-tetrahydro-2H-naphtho[2,1-*b*]carbazole-4-carboxylate (–)-8c**: (–)-**8c** was obtained as yellow foam (0.19 mmol scale of reaction; 38 mg, 35%). R_f = 0.35 (20% EtOAc in *n*-hexane).

^{1}H NMR (500 MHz, $CDCl_3$) δ 8.26 (d, *J* = 8.3 Hz, 1H), 8.03 (s, 1H), 7.96 (s, 1H), 7.86 – 7.83 (m, 1H), 7.62 (d, *J* = 8.4 Hz, 2H), 7.47 – 7.43 (m, 1H), 7.33 (t, *J* = 7.5 Hz, 1H), 7.10 (d, *J* = 8.3 Hz, 2H), 6.18 (t, *J* = 4.1 Hz, 1H), 5.94 (s, 1H), 3.64 (s, 3H), 2.44 (s, 3H), 2.28 (d, *J* = 3.4 Hz, 1H), 2.27 (s, 3H), 2.21 (s, 1H), 2.05 (dd, *J* = 13.7, 6.8 Hz, 2H), 1.35 (s, 3H), 1.25 (s, 3H).

^{13}C NMR (125 MHz, $CDCl_3$) δ 176.2, 169.4, 144.9, 143.3, 142.1, 139.0, 138.0, 137.9, 134.7, 130.7, 129.7, 129.7, 127.7, 127.3, 126.5, 126.5, 126.2, 124.0, 123.8, 123.7, 119.8, 116.0, 115.2, 107.7, 51.8, 47.5, 41.6, 29.5, 24.2, 23.7, 21.5, 21.0, 20.8.

IR (film) υ_{max} 3020, 1850, 1735, 1670, 1500, 1165, 900, 680 cm^{-1}.
HRMS (ESI) *m*/*z*: $[M + Na]^+$ Calcd for $[C_{33}H_{31}NO_6S + Na]^+$ 592.1764; found 592.1764.

$[\alpha]_{589}^{25.0}$ = –1.67 (*c* = 0.1, $CHCl_3$).

Synthesis of compound (–)-19 from (–)-8c:

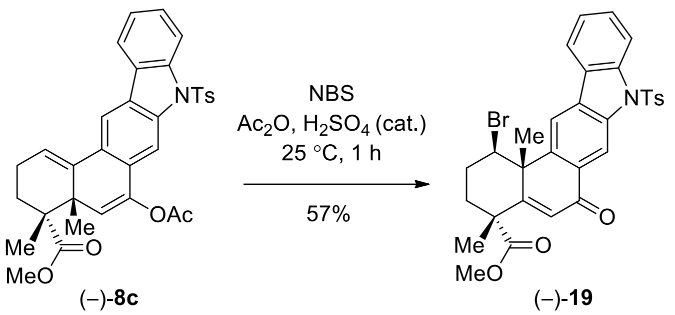

In an oven-dried round-bottom flask compound (–)-**8c** (32 mg, 0.06 mmol, 1.0 equiv.) was taken in 1 mL of Ac_2O and catalytic H_2SO_4 was added at 25 ºC. After 5 minutes solid NBS (11 mg, 0.061 mmol, 1.1 equiv.) was added to reaction mixture and run for 1 h at 25 ºC. After completion of the reaction (monitored by TLC), the reaction mixture was quenched with saturated aqueous $Na_2S_2O_3$ solution. The reaction mixture was then partitioned between water and EtOAc (3 mL X 2). The organic layer was washed with saturated $NaHCO_3$ solution (2 mL X 1) and dried over Na_2SO_4 and concentrated under reduced pressure. The crude product was purified by flash column chromatography with 16% EtOAc in *n*-hexane to afford (–)-**19** as yellow gel (21 mg, 57% yield).

Synthesis of (+)-**6**:

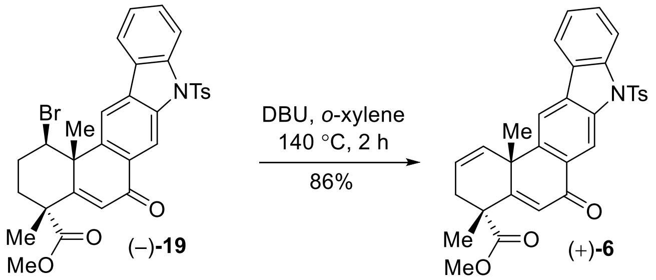

In an oven-dried round-bottom flask (–)-**19** (520 mg, 0.86 mmol, 1.0 equiv.) was taken in 8 mL of *o*-xylene and DBU (154 µL, 1.03 mmol, 1.2 equiv.) was added at 25 ºC. The reaction mixture was refluxed at 140 °C for 2 h. After completion of the reaction (monitored by TLC), water was added and extracted with ethyl acetate (10 mL X 3). The combined organic layer was washed with 4(*N*) HCl solution. Organic layer was dried over Na_2SO_4 and concentrated under reduced pressure. The crude product was purified on a silica gel column 16% EtOAc in *n*-hexane to get (+)-**6** as yellow foam (389 mg, 86% yield).

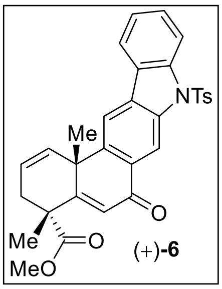

Methyl (4*R*,13b*R*)-4,13b-dimethyl-6-oxo-8-tosyl-4,6,8,13b-tetrahydro-3H-naphtho[2,1-*b*]carbazole-4-carboxylate [(+)-**6**]: (+)-**6** was obtained as yellow foam (0.86 mmol scale of reaction; 389 mg; 86%). R_f = 0.35 (30 % EtOAc in *n*-hexane).

^{1}H NMR (500 MHz, $CDCl_3$) δ 9.05 (s, 1H), 8.41 (d, *J* = 8.4 Hz, 1H), 8.14 (s, 1H), 8.02 (d, *J* = 7.7 Hz, 1H), 7.79 (d, *J* = 8.4 Hz, 2H), 7.60 (ddd, *J* = 8.5, 7.2, 1.3 Hz, 1H), 7.43 (t, *J* = 7.5 Hz, 1H), 7.14 (d, *J* = 8.2 Hz, 2H), 6.54 – 6.50 (m, 1H), 6.41 (s, 1H), 6.15 (dt, *J* = 9.5, 4.6 Hz, 1H), 3.75 (s, 3H), 2.84 (dd, *J* = 17.1, 5.1 Hz, 1H), 2.41 (ddd, *J* = 17.1, 4.2, 2.3 Hz, 1H), 2.29 (s, 3H), 1.73 (s, 3H), 1.64 (s, 3H).

^{13}C NMR (125 MHz, $CDCl_3$) δ 184.2, 175.1, 166.6, 146.1, 145.0, 140.0, 136.8, 134.8, 131.6, 130.1, 129.7, 129.6, 128.9, 127.2, 126.7, 125.2, 124.9, 124.0, 120.7, 116.5, 115.2, 113.1, 51.3, 49.6, 43.1, 34.7, 34.0, 26.5, 21.5.

IR (neat) υ_{max} 3056, 2918, 2877, 1762, 1678, 1569, 1423, 1225, 1074, 656 cm^{-1}.

HRMS (ESI) *m/z*: $[M+H]^+$ calcd. for $[C_{31}H_{27}NO_5S + H]^+$ 526.1683, found 526.1693.

$[\alpha]^{25}_{589}$ = +105.83 (*c* = 0.84, $CHCl_3$).

Allylic Oxidation of Olefin (+)-**6**:

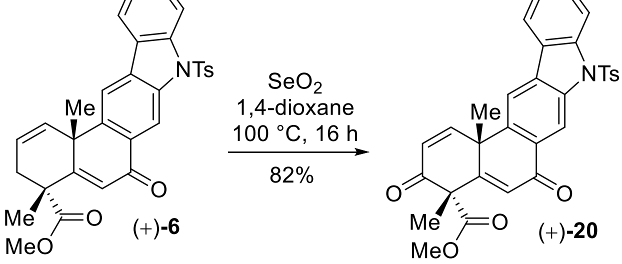

In an oven-dried round-bottom flask (+)-**6** (350 mg, 0.67 mmol; 1.0 equiv) was taken in 6 mL 1,4-dioxane. To this solution selenium dioxide (296 mg, 2.68 mmol; 4 equiv) was added at 25 °C. Then, the reaction mixture was heated to reflux at 100 °C and stirring was continued for 16 h until the full consumption of starting material (evaluated by TLC). The reaction mixture was diluted with water and extracted with ethyl acetate (10 mL X 3). The combined organic layer was washed with 4(N) HCl solution. Organic layer was dried over Na_2SO_4 and concentrated under reduced pressure. The crude product was purified by column chromatography using 30% EtOAc in *n*-Hexane to get (+)-**20** as colorless foam (296 mg, 82% yield).

Optimisation Table for Allylic Oxidation

Entry	Oxidant	Solvent	Temp.	Time	Yield
1.	CrO_3 (2.0 eq.)	AcOH	25 °C	6 h	*NR*
2.	CrO_3, 3,5-Dimethylpyrazole (5.0 eq., 5.0 eq.)	CH_2Cl_2	−15 °C	2 h	45%
3.	SeO_2 (0.5 eq.)/ aq. *t*-BuOOH (3.0 eq.)	CH_2Cl_2	25 °C	12 h	32%
4.	SeO_2 (4.0 eq.)	1,4-Dioxane	110 °C	16 h	82%
5.	$Pd(OH)_2$/C (5 mol %), K_2CO_3, *t*-BuOOH (in decane)	CH_2Cl_2	25 °C	24 h	52%

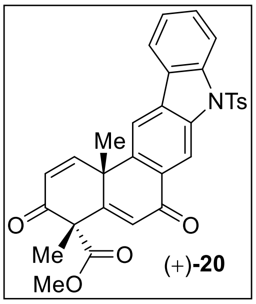

(+)-**20**

Methyl (4*S*,13b*R*)-4,13b-dimethyl-3,6-dioxo-8-tosyl-4,6,8,13b-tetrahydro-3H-naphtho[2,1-*b*]carbazole-4-carboxylate [(+)-**20**]: (+)-**20** was obtained as colourless foam (2.68 mmol scale of reaction; 296 mg; 82%). R_f = 0.3 (40 % EtOAc in *n*-hexane).

^{1}**H NMR** (500 MHz, $CDCl_3$) δ 9.12 (s, 1H), 8.43 (d, *J* = 8.4 Hz, 1H), 8.23 (s, 1H), 8.06 (d, *J* = 7.8 Hz, 1H), 7.82 (d, *J* = 8.3 Hz, 2H), 7.76 (d, *J* = 10.3 Hz, 1H), 7.67 – 7.62 (m, 1H), 7.47 (t, *J* = 7.6 Hz, 1H), 7.17 (d, *J* = 8.1 Hz, 2H), 6.50 (s, 1H), 6.44 (d, *J* = 10.2 Hz, 1H), 3.70 (s, 3H), 2.30 (s, 3H), 1.91 (s, 3H), 1.81 (s, 3H).

^{13}**C NMR** (125 MHz, $CDCl_3$) δ 194.2, 183.1, 170.6, 162.0, 151.8, 145.3, 142.2, 140.2, 137.4, 134.9, 130.5, 129.9, 129.5, 129.5, 128.3, 126.7, 126.5, 124.7, 124.2, 120.8, 116.3, 115.3, 113.6, 60.2, 53.4, 42.2, 39.4, 24.6, 21.5.

IR (neat) υ_{max} 3134, 2957, 2884, 1768, 1720, 1584, 1451, 1257, 1081, 663 cm^{-1}.
HRMS (ESI) *m/z*: $[M+H]^+$ calcd. for $[C_{31}H_{25}NO_6S + H]^+$ 540.1475, found 540.1492.

$[\alpha]^{25}_{589}$ = +103.57 (*c* = 0.87, $CHCl_3$).

Partial Hydrogenation of (+)-**20**:

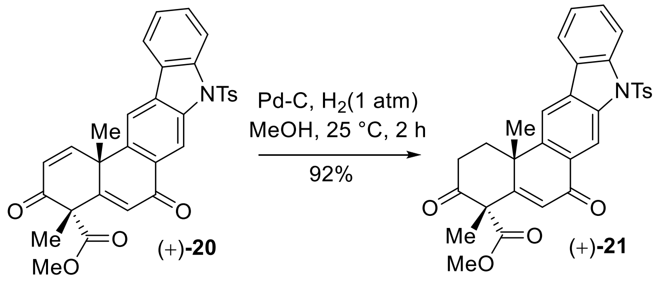

In an oven-dried round-bottom flask compound (+)-**20** (50 mg, 0.092 mmol, 1.0 equiv.) was taken in 3 mL of methanol and degassed with N_2 balloon for 10 min and then 2.5 mg of Pd/C (5% w/w) was added to the reaction mixture. H_2 gas was purged constantly at 25 ºC using a H_2 gas balloon for 2 h until the full consumption of starting material. Then the solvent was evaporated and the crude product was purified by column chromatography with 30% EtOAc in *n*-Hexane to afford compound (+)-**21** with partial hydrogenation as white foam (45.8 mg, 92% yield).

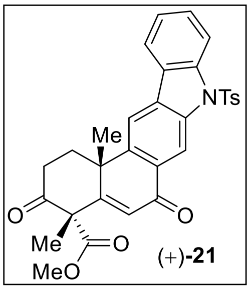

(+)-**21**

Methyl (4*S*,13b*R*)-**4,13b-dimethyl-3,6-dioxo-8-tosyl-2,3,4,6,8,13b-hexahydro-1H-naphtho[2,1-b]carbazole-4-carboxylate** [(+)-**21**]: (+)-**21** was obtained as colourless foam (0.092 mmol scale of reaction; 45.8 mg; 92%). R_f = 0.27 (40 % EtOAc in *n*-hexane).

^{1}H NMR (500 MHz, $CDCl_3$) δ 9.11 (s, 1H), 8.42 (d, *J* = 8.4 Hz, 1H), 8.11 (s, 1H), 8.01 (d, *J* = 7.8 Hz, 1H), 7.83 (d, *J* = 8.1 Hz, 2H), 7.62 (t, *J* = 7.2 Hz, 1H), 7.45 (d, *J* = 7.5 Hz, 1H), 7.16 (d, *J* = 8.2 Hz, 2H), 6.56 (s, 1H), 3.73 (s, 3H), 3.02 – 2.94 (m, 2H), 2.73 (t, *J* = 7.0 Hz, 1H), 2.38 (td, *J* = 8.0, 4.0 Hz, 1H), 2.30 (s, 3H), 1.77 (s, 3H), 1.50 (s, 3H).

^{13}C NMR (125 MHz, $CDCl_3$) δ 206.6, 183.8, 169.9, 165.1, 145.2, 144.6, 140.1, 137.2, 135.0, 130.3, 129., 129.3, 126.8, 125.7, 124.9, 124.1, 120.8, 117.1, 115.2, 113.0, 60.2, 53.6, 40.3, 34.9, 32.0, 27.2, 24.2, 21.6, 14.1.

IR (neat) υ_{max} 3147, 3042, 2981, 1752, 1704, 1582, 1482, 1223, 1068, 753 cm^{-1}.
HRMS (ESI) *m/z*: $[M+H]^+$ calcd. for $[C_{31}H_{27}NO_6S + H]^+$ 542.1632, found 542.1639.

$[\alpha]^{25}_{589}$ = +62.54 (*c* = 0.78, $CHCl_3$).

Procedure for the Synthesis of Diketone (+)-5:

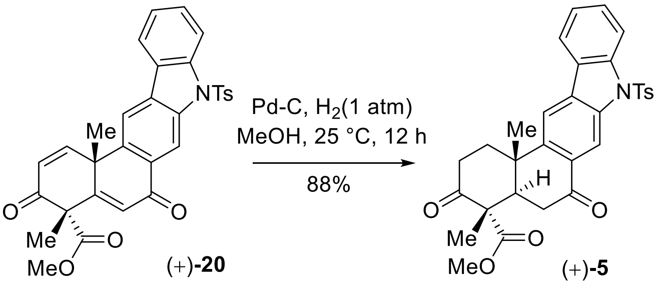

(+)-**20** → (+)-**5**

In an oven-dried round-bottom flask (+)-**20** (260 mg, 0.48 mmol, 1.0 equiv.) was taken in 5 mL of methanol and degassed with N_2 balloon for 10 min and then 13 mg of Pd/C (5% w/w) was added to the reaction mixture. H_2 gas was purged constantly at 25 ºC using a H_2 gas balloon for 12 h until the full consumption of starting material. Then the solvent was evaporated and the crude product was purified by column chromatography with 30% EtOAc in *n*-Hexane to afford diketone (+)-**5** as colorless foam (226 mg, 88% yield).

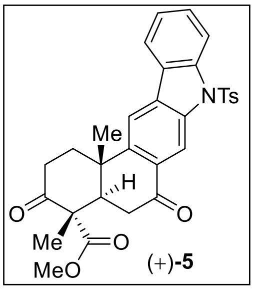

(+)-**5**

Methyl (4*S*,13b*S*)**-4,13b-dimethyl-3,6-dioxo-8-tosyl-2,3,4,4a,5,6,8,13b-octahydro-1H-naphtho[2,1-*b*]carbazole-4-carboxylate** ([(+)-**5**]: (+)-**5** was obtained as colourless foam (0.48 mmol scale of reaction; 226 mg; 95%). R_f = 0.3 (40 % EtOAc in *n*-hexane).

^{1}H NMR (500 MHz, $CDCl_3$) δ 8.98 (s, 1H), 8.39 (d, *J* = 8.2 Hz, 1H), 7.98 (d, *J* = 7.8 Hz, 1H), 7.91 (s, 1H), 7.79 (d, *J* = 8.0 Hz, 2H), 7.61 (t, *J* = 7.7 Hz, 1H), 7.43 (t, *J* = 7.7 Hz, 1H), 7.17 (d, *J* = 8.6 Hz, 2H), 3.77 (s, 3H), 3.37 (dd, *J* = 14.3, 3.7 Hz, 1H), 2.98 – 2.91 (m, 2H), 2.84 (d, *J* = 8.3 Hz, 2H), 2.71 (dd, *J* = 15.3, 4.8 Hz, 1H), 2.48 – 2.43 (m, 1H), 2.31 (s, 3H), 1.58 (s, 3H), 1.54 (s, 3H).

^{13}C NMR (125 MHz, $CDCl_3$) δ 208.4, 196.1, 172.4, 148.4, 145.2, 140.1, 136.9, 134.9, 131.1, 129.9, 129.8, 129.2, 126.7, 125.1, 124.1, 120.9, 115.3, 115.2, 114.4, 60.7, 53.0, 44.9, 37.2, 37.1, 36.5, 34.7, 23.2, 21.6, 16.8.

IR (neat) υ_{max} 3163, 3064, 2972, 1761, 1727, 1519, 1432, 1261, 1063, 689 cm^{-1}.
HRMS (ESI) *m/z*: $[M+ Na]^+$ calcd. for $[C_{31}H_{29}NO_6S + Na]^+$ 566.1608, found 566.1605.

$[\alpha]^{25}_{589}$ = +84.00 (*c* = 0.84, $CHCl_3$).

Procedure for the Synthesis of Diol (+)-**22**:

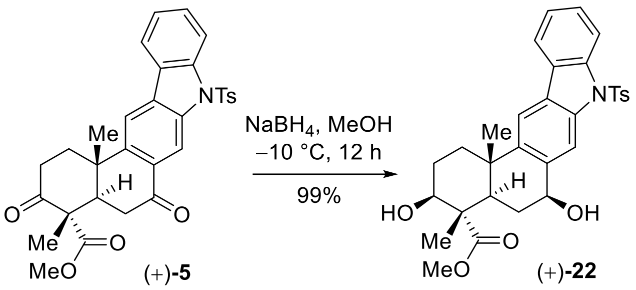

In an oven-dried round-bottom flask (+)-**5** (190 mg, 0.35 mmol, 1.0 equiv.) in MeOH (4 mL) at −10 ºC, $NaBH_4$ (16 mg, 0.42 mmol, 1.2 equiv.) was added portion wise and the reaction mixture was stirred at same temperature for 15 min. After complete consumption of starting material (judged by TLC analysis), it was quenched with saturated NH_4Cl (5 mL) and extracted with EtOAc (5 mL X 2). The organic layers were dried over Na_2SO_4 and concentrated on a rotary evaporator under reduced pressure. The crude products were purified by flash chromatography with 50% EtOAc in *n*-hexane to afford (+)-**22** as colourless oil (188 mg, 99% yield).

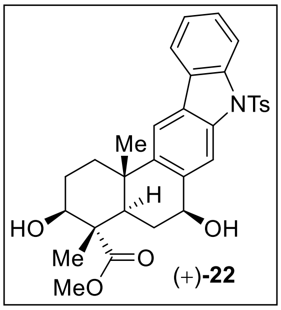

Methyl (3*S*,4*S*,6*S*,13b*S*)-**3,6-dihydroxy-4,13b-dimethyl-8-tosyl-2,3,4,4a,5,6,8,13b-octahydro-1H-naphtho[2,1-*b*]carbazole-4-carboxylate** [(+)-**22**]: (+)-**22** was obtained as colourless oil (0.35 mmol scale of reaction; 188 mg; 99%). R_f = 0.25 (50 % EtOAc in *n*-hexane).

^{1}H NMR (500 MHz, $CDCl_3$) δ 8.44 (s, 1H), 8.31 (d, *J* = 8.4 Hz, 1H), 7.89 (s, 1H), 7.74 (d, *J* = 7.9 Hz, 3H), 7.49 (t, *J* = 7.8 Hz, 1H), 7.36 (t, *J* = 7.5 Hz, 1H), 7.14 (d, *J* = 8.1 Hz, 2H), 5.05 (t, *J* = 8.6 Hz, 1H), 4.06 (dd, *J* = 11.7, 4.6 Hz, 1H), 3.80 (s, 3H), 2.49 (d, *J* = 12.9 Hz, 1H), 2.30 (s, 3H), 2.23 (dd, *J* = 12.1, 2.3 Hz, 1H), 1.99 (s, 2H), 1.88 (d, *J* = 12.6 Hz, 1H), 1.70 (dt, *J* = 13.7, 6.6 Hz, 2H), 1.36 (s, 3H), 1.33 (s, 3H).

^{13}C NMR (125 MHz, $CDCl_3$) δ 177.5, 144.8, 138.9, 137.8, 137.0, 135.1, 129.7, 127.4, 126.6, 126.1, 126.0, 123.8, 119.8, 115.6, 115.1, 113.8, 75.0, 71.0, 53.4, 52.5, 44.0, 37.8, 37.1, 32.2, 27.1, 26.1, 21.5, 10.8.

IR (neat) υ_{max} 3668, 3543, 3192, 3072, 1773, 1752, 1541, 1162, 1021, 653 cm^{-1}.
HRMS (ESI) *m/z*: $[M+ Na]^+$ calcd. for $[C_{31}H_{33}NO_6S + Na]^+$ 570.1921, found 570.1908.

$[\alpha]^{25}_{589}$ = +94.20 (*c* = 0.89, $CHCl_3$).

Procedure for the preparation of (+)-**Xiamycin D** [(+)-**2b**]**:**

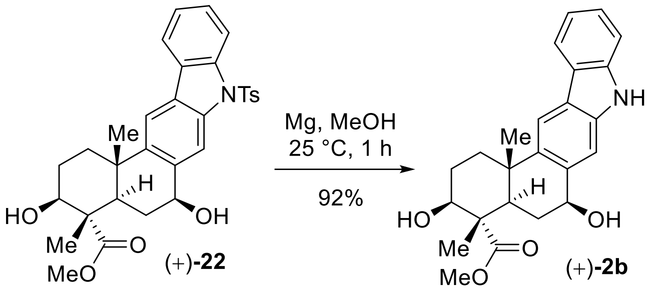

To a stirred solution of dihydroxy (+)-**22** (76 mg, 0.139 mmol, 1.0 equiv.) in MeOH (3 mL) at 25 °C magnesium powder (6.7 mg, 0.278 mmol, 2.0 equiv.) was added and allowed to stir the resulting reaction mixture vigorously for 1 h at the same temperature. After the full consumption of the starting materials, it was quenched with saturated NH_4Cl solution (2 mL), and the reaction mixture was extracted with EtOAc (5 mL X 3). All organic layers were separated, dried over Na_2SO_4 and concentrated in rotary evaporator under reduced pressure. The residue was purified by silica gel column chromatography with 70% EtOAc in *n*-hexane to afford naturally occurring Xiamycin D (+)-**2b** as yellow oil (50.3 mg, 92% yield).

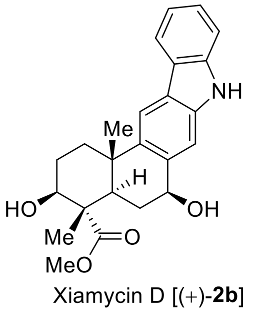

Xiamycin D [(+)-**2b**]

Methyl (3*S*,4*S*,4a*R*,6*S*,13b*S*)**-3,6-dihydroxy-4,13b-dimethyl-2,3,4,4a,5,6,8,13b-octahydro-1H-naphtho[2,1-*b*]carbazole-4-carboxylate** [(+)-**2b**]**:** Xiamycin D [(+)-**2b**] was obtained as yellow oil (0.139 mmol scale of reaction, 50.3 mg, 92% yield). R_f = 0.25 (50% EtOAc in hexane)

^{1}H NMR (400 MHz, CD_3OD) δ 8.00 (d, *J* = 7.7 Hz, 1H), 7.93 (s, 1H), 7.56 (s, 1H), 7.37 (d, *J* = 8.0 Hz, 1H), 7.31 (t, *J* = 7.5 Hz, 1H), 7.10 (t, *J* = 7.4 Hz, 1H), 4.85 – 4.83 (m, 1H), 4.08 – 4.03 (m, 1H), 3.73 (s, 3H), 2.63 (dt, *J* = 12.3, 3.1 Hz, 1H), 2.16 (d, *J* = 12.8 Hz, 1H), 1.97 (d, *J* = 2.5 Hz, 1H), 1.92 – 1.85 (m, 2H), 1.69 (dq, *J* = 13.4, 6.6, 5.9 Hz, 2H), 1.37 (s, 3H), 1.28 (s, 3H).

^{13}C NMR (125 MHz, CD_3OD) δ 179.4, 142.4, 141.3, 140.2, 137.3, 126.5, 124.4, 124.2, 120.9, 119.5, 116.4, 111.6, 110.1, 76.1, 71.9, 55.3, 52.7, 46.3, 39.1, 38.9, 33.2, 28.5, 26.8, 11.4.

IR (neat) υ_{max} 3403, 2927, 2823, 1712, 1628, 1443, 1353, 1197, 1083, 903, 810 cm^{-1}.
HRMS (ESI) *m/z*: $[M+ Na]^+$ calcd. for $[C_{24}H_{27}O_4N + Na]^+$ 416.1832, found 416.1806.

$[\alpha]^{25}_{589}$ = +121.0 (*c* = 0.25, CH_3OH); lit.[2] $[\alpha]_D^{20}$= +134 (*c* = 0.1, CH_3OH), lit.[1] $[\alpha]_D^{20}$ = +133.9 (*c* = 0.1, CH_3OH).

Comparison of ^{1}H-NMR Data of (+)-Xiamycin D [(+)-**2b**] of this report with natural (+)-**2b** by Oh[1] and literature of (+)-**2b** by Dethe[2]:

Oh's Isolation report (+)-Xiamycin D [(+)-**2b**] (^{1}H-NMR, 500 MHz, CD_3OD)[1]			
δ (ppm)	Int.	mult.	J (Hz)
8.01	1H	d	*J* = 8.0 Hz
7.94	1H	s	-
7.58	1H	s	-
7.38	1H	d	*J* = 8.0 Hz
7.32	1H	dd	*J* = 8.0, 8.0 Hz
7.11	1H	dd	*J* = 8.0, 8.0 Hz
4.85	1H	m	-
4.07	1H	dd	*J* = 9.5, 6.5 Hz
3.74	3H	s	-
2.64	1H	ddd	*J* = 13.0, 3.5, 3.5 Hz
2.18	1H	dd	*J* = 13.0, 1.5 Hz
2.00	1H	m	-
1.92	2H	m	-
1.70	1H	m	-
1.68	1H	m	-
1.38	3H	s	-
1.28	3H	s	-

Dethe's report (+)-Xiamycin D [(+)-**2b**] (^{1}H-NMR, 500 MHz, CD_3OD)[2]			
δ (ppm)	Int.	mult.	J (Hz)
8.00	1H	d	*J* = 8.0 Hz,
7.94	1H	s	-
7.57	1H	s	-
7.38	1H	d	*J* = 7.9 Hz

7.34-7.30	1H	m	-
7.12-7.09	1H	m	-
4.85−4.83	1H	m	-
4.10−4.05	1H	m	-
3.74	3H	s	-
2.64	1H	d	*J* = 12.8 Hz
2.17	1H	d	*J* = 12.7 Hz
2.03-1.95	1H	m	-
1.89	2H	dd	*J* = 8.0, 2.6 Hz
1.70	2H	dd	*J* = 11.4, 6.8, Hz
1.38	3H	s	-
1.28	3H	s	-

This report (+)-Xiamycin D [(+)-**2b**] (^{1}H-NMR, 400 MHz, CD_3OD)			
δ (ppm)	Int.	mult.	J (Hz)
8.00	1H	d	*J* = 7.7 Hz
7.93	1H	s	-
7.56	1H	s	-
7.37	1H	d	*J* = 8.0 Hz
7.31	1H	t	*J* = 7.5 Hz
7.10	1H	t	*J* = 7.4 Hz
4.85 – 4.83	1H	m	-
4.08 – 4.03	1H	m	-
3.73	3H	s	-
2.63	1H	dt	*J* = 12.3, 3.1 Hz
2.16	1H	d	*J* = 12.8 Hz
1.97	1H	d	*J* = 2.5 Hz
1.92 – 1.85	2H	m	-
1.69	2H	dq	*J* = 13.4, 6.6, 5.9 Hz
1.37	3H	s	-
1.28	3H	s	-

Comparison of ^{13}C-NMR Data:

Oh's report (+)-Xiamycin D [(+)-**2b**] (^{13}C-NMR, 150 MHz, CD_3OD)[1]	Dethe's report (+)-Xiamycin D [(+)-**2b**] (^{13}C-NMR, 126 MHz, CD_3OD)[2]	This report (+)-Xiamycin D [(+)-**2b**] (^{13}C-NMR, 125 MHz, CD_3OD)
179.4	179.4	179.4
142.4	142.3	142.4
141.3	141.2	141.3
140.2	140.2	140.2
137.3	137.2	137.3
126.5	126.5	126.5
124.4	124.3	124.4
124.2	124.2	124.2
120.9	120.8	120.9
119.5	119.4	119.5
116.4	116.3	116.4
111.6	111.5	111.6
110.1	110.0	110.1
76.1	76.1	76.1
72.0	71.9	71.9
55.3	55.3	55.3
52.7	52.6	52.7
46.3	46.3	46.3
39.1	39.1	39.1
39.0	38.9	38.9
33.2	33.2	33.2
28.5	28.4	28.5
26.8	26.7	26.8
11.4	11.4	11.4

Procedure for the preparation of (+)-**Xiamycin E** [(+)-**2c**]**:**

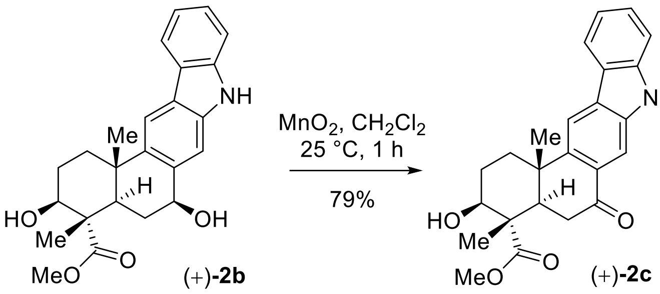

(+)-**2b** (114 mg, 0.289 mmol, 1.0 equiv.) was taken in dry CH_2Cl_2 (5 mL), to the solution MnO_2 (151 mg, 1.74 mmol, 6.0 equiv.) was added to it at 25 °C and stirred for 6 h at the same temperature. After completion of the reaction (monitored by TLC), the reaction mixture was filtered through celite pad. The organic layer was collected and concentrated under reduced pressure. The crude product was purified by flash column chromatography 40% EtOAc in *n*-hexane to afford naturally occurring Xiamycin E (+)-**2c** as yellow oil (89.4 mg, 79% yield).

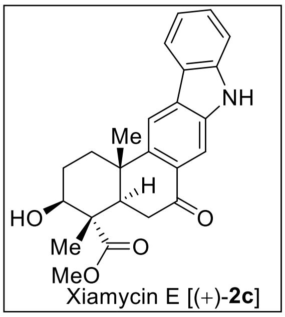

Methyl (3*S*,4*S*,4a*R*,13b*S*)-**3-hydroxy-4,13b-dimethyl-6-oxo-2,3,4,4a,5,6,8,13b-octahydro-1H-naphtho[2,1-*b*]carbazole-4-carboxylate** [(+)-**2c**]**:** (+)-Xiamycin E [(+)-**2c**] was obtained as yellow oil (0.289 mmol scale of reaction; 280 mg; 79% yield). R_f = 0.2 (40% EtOAc in *n*-hexane).

^{1}H NMR (500 MHz, CD_3OD) δ 8.16 (d, *J* = 7.8 Hz, 1H), 8.14 (s, 1H), 8.07 (s, 1H), 7.48 – 7.46 (m, 2H), 7.21 (ddd, *J* = 8.1, 5.1, 2.9 Hz, 1H), 4.12 (dd, *J* = 9.3, 6.0 Hz, 1H), 3.73 (s, 3H), 2.97 (dd, *J* = 18.0, 14.1 Hz, 1H), 2.75 – 2.70 (m, 1H), 2.63 (dd, *J* = 14.2, 3.6 Hz, 1H), 2.20 (dd, *J* = 18.0, 3.6 Hz, 1H), 1.99 – 1.94 (m, 3H), 1.36 (s, 3H), 1.35 (s, 3H).

^{13}C NMR (125 MHz, CD_3OD) δ 200.6, 178.5, 147.4, 143.8, 139.5, 129.9, 129.2, 128.6, 123.6, 122.2, 120.3, 115.9, 112.2, 110.6, 75.9, 54.7, 52.8, 46.8, 38.6, 38.5, 38.0, 28.1, 24.8, 10.9.

^{1}H NMR (500 MHz, $CDCl_3$) δ 8.41 (s, 1H), 8.15 (s, 1H), 8.12 (d, *J* = 7.9 Hz, 1H), 8.03 (s, 1H), 7.53 – 7.44 (m, 2H), 7.27 (dd, *J* = 6.6, 1.2 Hz, 1H), 4.17 (dd, *J* = 11.3, 4.0 Hz, 1H), 3.75 (s, 3H), 2.94 (dd, *J* = 18.0, 14.1 Hz, 1H), 2.74 – 2.64 (m, 2H), 2.45 (dd, *J* = 18.1, 3.6 Hz, 1H), 2.08 – 2.04 (m, 1H), 2.02 – 1.94 (m, 2H), 1.40 (s, 3H), 1.36 (s, 3H).

^{13}C NMR (125 MHz, $CDCl_3$) δ 198.4, 176.8, 146.1, 141.6, 137.7, 128.6, 128.4, 127.7, 122.5, 121.2, 119.9, 114.8, 111.1, 109.8, 74.8, 52.9, 52.5, 44.6, 37.6, 37.4, 36.5, 26.9, 24.6, 10.5.

IR (neat) υ_{max} 3372, 2895, 2853, 1706, 1593, 1477, 1213, 969, 813, 717 cm^{-1}.
HRMS (ESI) *m/z*: $[M+ Na]^+$ calcd. for $[C_{24}H_{25}O_4N + Na]^+$ 414.1676, found 414.1670.

$[\alpha]^{25}_{589}$ = +29.4 (*c* = 0.27, CH_3OH); lit.[2] $[\alpha]_D^{20}$= +22 (*c* = 0.2, CH_3OH), lit.[1] $[\alpha]_D^{20}$ = +23.6 (*c* = 0.1, CH_3OH).

Comparison of ^{1}H-NMR Data of (+)-Xiamycin E [(+)-**2c**] of this report with natural (+)-**2c** by Oh[1] and literature of (+)-**2c** by Dethe[2]:

Oh's Isolation report (+)-Xiamycin E [(+)-**2c**] (^{1}H-NMR, 500 MHz, CD_3OD)[1]			
δ (ppm)	Int.	mult.	*J* (Hz)
8.15	1H	d	*J* = 8.0 Hz
8.14	1H	s	-
8.06	1H	s	-
7.46	1H	d	*J* = 8.0 Hz
7.45	1H	dd	*J* = 8.0, 8.0 Hz
7.20	1H	dd	*J* = 8.0, 8.0 Hz
4.11	1H	m	-
3.72	3H	s	-
2.97	1H	dd	*J* = 18.0, 14.0, Hz

2.74	1H	m	-
2.63	1H	dd	*J* = 14.0, 3.5 Hz
2.19	1H	dd	*J* = 18.0, 3.5 Hz
1.98	2H	m	-
1.95	1H	m	-
1.37	3H	s	-
1.35	3H	s	-

Dethe's report (+)-Xiamycin E [(+)-**2c**] (^{1}H-NMR, 500 MHz, CD_3OD)[2]			
δ (ppm)	Int.	mult.	*J* (Hz)
8.15	1H	d	*J* = 7.0 Hz
8.14	1H	s	-
8.06	1H	s	-
7.48-7.44	2H	m	-
7.20	1H	ddd	*J* = 8.0, 5.2, 2.8 Hz
4.13-4.10	1H	m	-
3.72	3H	s	-
3.0-2.94	1H	m	-
2.75-2.72	1H	m	-
2.65-2.61	1H	m	-
2.19	1H	dd	*J* = 18.0, 3.5 Hz
1.99-1.94	3H	m	-
1.36	3H	s	-
1.35	3H	s	-

This report (+)-Xiamycin E [(+)-**2c**] (^{1}H-NMR, 500 MHz, CD_3OD)			
δ (ppm)	Int.	mult.	*J* (Hz)
8.16	1H	d	*J* = 7.8 Hz
8.14	1H	s	-
8.07	1H	s	-
7.48 – 7.46	2H	m	-
7.21	1H	ddd	*J* = 8.1, 5.1, 2.9 Hz
4.12	1H	dd	*J* = 9.3, 6.0 Hz
3.73	3H	s	-
2.97	1H	dd	*J* = 18.0, 14.1 Hz
2.75 – 2.70	1H	m	-
2.63	1H	dd	*J* = 14.2, 3.6 Hz
2.20	1H	dd	*J* = 18.0, 3.6 Hz
1.99 – 1.94	3H	m	-
1.36	3H	s	-
1.35	3H	s	-

Comparison of ^{13}C-NMR Data:

Oh's Isolation report (+)-Xiamycin E [(+)-**2c**] (^{13}C-NMR, 150 MHz, CD_3OD)[1]	Dethe's report (+)-Xiamycin E [(+)-**2c**] (^{13}C-NMR, 125 MHz, CD_3OD)[2]	This report (+)-Xiamycin E [(+)-**2c**] (^{13}C-NMR, 125 MHz, CD_3OD)
200.7	200.7	200.6
178.5	178.5	178.5
147.4	147.4	147.4
143.9	143.9	143.8
139.5	139.5	139.5
129.9	126.9	129.9
129.2	129.2	129.2

128.6	128.6	128.6
123.6	123.6	123.6
122.2	122.2	122.2
120.3	120.3	120.3
116.0	116.0	115.9
112.7	112.2	112.2
110.6	110.6	110.6
75.9	75.9	75.9
54.7	54.7	54.7
52.8	52.8	52.8
46.8	46.8	46.8
38.7	38.7	38.6
38.6	38.6	38.5
38.1	38.0	38.0
28.1	28.1	28.1
24.8	24.8	24.8
10.9	11.0	10.9

Procedure for the preparation of Xiamycin F [(+)-**2d**]**:**

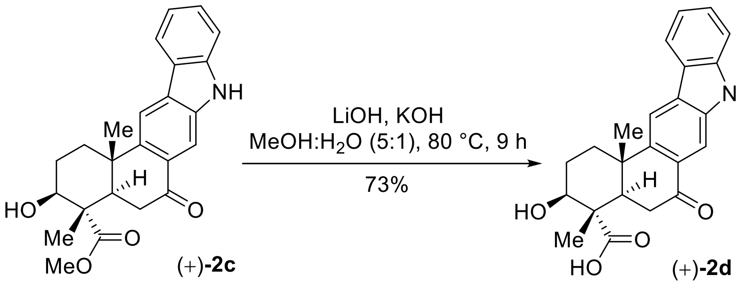

In an oven dried round-bottom flask (+)-Xiamycin E [(+)-**2c**] (65 mg, 0.166 mmol, 1.0 equiv.) was taken in a mixture of methanol and water [MeOH: H_2O (5:1)]. To the solution KOH (280 mg, 5.0 mmol, 30.0 equiv.) and LiOH (139 mg, 3.32 mmol, 20 equiv.) were added subsequently and reaction mixture was refluxed for 9 h at 80 ºC. After completion of the reaction confirmed by TLC, reaction mixture was quenched with 4(*N*) HCl at 0 ºC and the pH of the reaction mixture was adjusted to ~1-2. Then the reaction mixture was extracted with ethyl acetate (8

mL X 2). The organic layer was collected, dried over Na_2SO_4 and concentrated under reduced pressure. The crude product was purified by flash column chromatography with ~80-90% EtOAc in *n*-Hexane to afford Xiamycin F [(+)-**2d**] as yellow foam (45.7 mg, 73% yield).

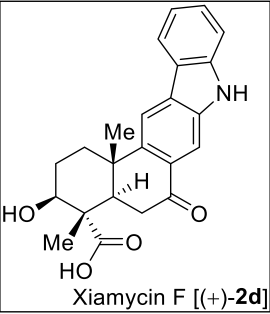

Xiamycin F [(+)-**2d**]

(3*S*,4*S*,4a*R*,13b*S*)-**3-Hydroxy-4,13b-dimethyl-6-oxo-2,3,4,4a,5,6,8,13b-octahydro-1H-naphtho[2,1-*b*]carbazole-4-carboxylic acid** [(+)-**2d**]**:** Xiamycin F [(+)-**2d**] was obtained as yellow foam (0.166 mmol scale of reaction, 45.7 mg of product, 73% yield). R_f = 0.18 (70% EtOAc in hexane).

^{1}H NMR (500 MHz, CD_3OD) δ 8.14 (d, *J* = 7.8 Hz, 1H), 8.10 (s, 1H), 8.05 (s, 1H), 7.46 (d, *J* = 5.3 Hz, 2H), 7.24 – 7.16 (m, 1H), 4.11 (d, *J* = 8.7 Hz, 1H), 2.93 (t, *J* = 15.9 Hz, 1H), 2.74 – 2.62 (m, 2H), 2.49 (d, *J* = 17.5 Hz, 1H), 1.94 (dt, *J* = 20.2, 9.8 Hz, 3H), 1.34 (s, 3H), 1.32 (s, 3H).

^{13}C NMR (125 MHz, CD_3OD) δ 201.2, 180.2, 147.4, 143.4, 139.0, 129.4, 128.9, 128.1, 123.2, 121.7, 119.8, 115.5, 111.7, 110.1, 75.8, 54.4, 46.2, 38.4, 38.2, 37.7, 27.7, 24.4, 11.2.

IR (neat) υ_{max} 3402, 2937, 2203, 1697, 1633, 1453, 1190, 1013 cm^{-1}.
HRMS (ESI) *m/z*: $[M+ Na]^+$ calcd. for $[C_{23}H_{23}O_4N + Na]^+$ 400.1519, found 400.1506.

$[\alpha]^{25}_{589}$ = +110.0 (*c* = 0.45, CH_3OH); lit.[4] $[\alpha]_D^{22}$= +130.1 (*c* = 0.4, CH_3OH).

Comparison of ^{1}H-NMR Data of Xiamycin F [(+)-**2d**] of this report with isolation of (+)-**2d** by Zhang[3] and literature of (+)-**2d** by Sarpong[4]**:**

Zhang's Isolation report (+)-Xiamycin F [(+)-**2d**] (^{1}H-NMR, 600 MHz, CD_3OD)[3]			
δ (ppm)	Int.	mult.	*J* (Hz)
8.16	1H	d	*J* = 8.0 Hz
8.12	1H	s	-
8.06	1H	s	-
7.47	1H	m	-
7.47	1H	m	-
7.20	1H	m	-
4.13	1H	m	-
2.96	1H	m	-
2.73	2H	m	-
2.67	1H	m	-
2.43	1H	d	*J* = 17.5 Hz
1.96	2H	m	-
1.94	1H	m	-
1.39	3H	s	-
1.33	3H	s	-

Sarpong's report (+)-Xiamycin F [(+)-**2d**] (^{1}H-NMR, 700 MHz, CD_3OD)[4]			
δ (ppm)	Int.	mult.	*J* (Hz)
8.16–8.13	1H	d	*J* = 7.6 Hz
8.12	1H	s	-
8.05	1H	s	-
7.47–7.42	2H	m	-
7.19	1H	td	*J* = 6.8, 2.0 Hz
4.13–4.06	1H	m	-
2.91	1H	t	*J* = 15.9 Hz
2.72–2.64	2H	m	-
2.57	1H	d	*J* = 11.8 Hz
1.98–1.90	3H	m	-

1.35	3H	s	-
1.30	3H	s	-

This report (+)-Xiamycin F [(+)-**2d**] (^{1}H-NMR, 500 MHz, CD_3OD)			
δ (ppm)	Int.	mult.	*J* (Hz)
8.14	1H	d	*J* = 7.8 Hz
8.10	1H	s	-
8.05	1H	s	-
7.46	2H	d	*J* = 5.3 Hz
7.24 – 7.16	1H	m	-
4.11	1H	d	*J* = 8.7 Hz
2.93	1H	t	*J* = 15.9 Hz
2.74 – 2.62	2H	m	-
2.49	1H	d	*J* = 17.5 Hz
1.94	3H	dt	*J* = 20.2, 9.8 Hz
1.34	3H	s	-
1.32	3H	s	-

Comparison of ^{13}C-NMR Data:

Zhang's Isolation report (+)-Xiamycin F [(+)-**2d**] (^{13}C-NMR, 125.77 MHz, CD_3OD)[3]	Sarpong's report (+)-Xiamycin F [(+)-**2d**] (^{13}C-NMR, 176 MHz, CD_3OD)[4]	This report (+)-Xiamycin F [(+)-**2d**] (^{13}C-NMR, 125 MHz, CD_3OD)
201.3	201.1	201.2
180.0	180.1	180.2
147.5	147.7	147.4
143.9	144.0	143.4
139.6	139.6	139.0
130.2	130.1	129.4

129.5	129.4	128.9
128.6	128.8	128.1
123.8	123.8	123.2
122.2	122.3	121.7
120.3	120.4	119.8
116.1	116.2	115.5
112.2	112.3	111.7
110.7	110.7	110.1
76.8	76.0	75.8
54.3	54.3	54.4
46.8	46.9	46.2
38.7	38.8	38.4
38.2	38.7	38.2
38.2	38.3	37.7
28.3	28.4	27.7
24.8	24.9	24.4
11.2	11.2	11.2

Procedure for the preparation of Xiamycin C [(+)-**2a**]:

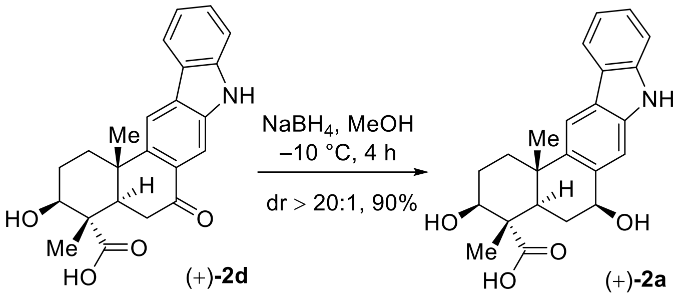

(+)-**2d** (15 mg, 0.040 mmol, 1.0 equiv.) was taken in MeOH (2 mL) and cooled the reaction vessel at −10 °C. $NaBH_4$ was slowly added (1.5 mg, 0.040 mmol, 1.0 equiv.) portion wise and it was allowed to stir for an additional 4 h. Upon completion of the reaction (judged by running TLC), the reaction was quenched with saturated aqueous NH_4Cl solution (2 mL), and the aqueous mixture was extracted with EtOAc (5 mL X 3). All organic layers were dried over Na_2SO_4 and concentrated in a rotary evaporator under vacuum to give crude product, which

was purified by column chromatography with 15% MeOH in CH_2Cl_2 to furnish Xiamycin C (+)-**2a** as yellow foam (13 mg, 90% yield).

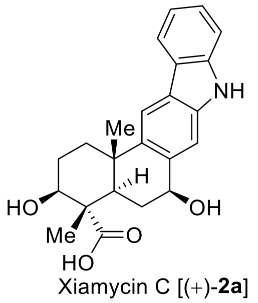

(3*S*,4*S*,4a*R*,6*S*,13b*S*)**-3,6-Dihydroxy-4,13b-dimethyl-2,3,4,4a,5,6,8,13b-octahydro-1H-naphtho[2,1-*b*]carbazole-4-carboxylic acid** [(+)-**2a**]**:** Xiamycin C [(+)-**2a**] was obtained as yellow foam (0.040 mmol scale of reaction, 13 mg of product, 90% yield). R_f = 0.15 (10% MeOH in CH_2Cl_2).

^{1}H NMR (500 MHz, CD_3OD) δ 8.02 (d, *J* = 7.6 Hz, 1H), 7.95 (s, 1H), 7.60 (s, 1H), 7.42 – 7.38 (m, 1H), 7.33 (t, *J* = 7.5 Hz, 1H), 7.12 (t, *J* = 7.5 Hz, 1H), 4.91 (d, *J* = 8.5 Hz, 1H), 4.10 (t, *J* = 8.2 Hz, 1H), 2.64 (d, *J* = 12.8 Hz, 1H), 2.22 (d, *J* = 12.3 Hz, 1H), 2.01 (q, *J* = 11.9 Hz, 2H), 1.93 (d, *J* = 12.1 Hz, 3H), 1.73 (dd, *J* = 13.0, 7.5 Hz, 1H), 1.40 (s, 3H), 1.28 (s, 3H).

^{13}C NMR (125 MHz, CD_3OD) δ 180.3, 142.4, 141.6, 140.2, 137.5, 126.4, 124.4, 124.2, 120.8, 119.4, 116.4, 111.6, 110.1, 76.3, 72.2, 54.9, 46.1, 39.2, 39.0, 33.2, 28.6, 26.8, 11.8.

IR (neat) υ_{max} 3656, 3579, 2937, 2013, 1692, 1353, 1197, 997 cm^{-1}.

HRMS (ESI) *m/z*: $[M+ Na]^+$ calcd. for $[C_{23}H_{25}O_4N + Na]^+$ 402.1676, found 402.1677.

$[\alpha]^{25}_{589}$ = +117.2 (*c* = 0.15, CH_3OH); lit.[4] $[\alpha]_D{}^{22}$= +120.6 (*c* = 0.1, CH_3OH), lit.[1] $[\alpha]_D{}^{20}$ = +123.6 (*c* = 0.1, CH_3OH).

Comparison of ^{1}H-NMR Data of Xiamycin C [(+)-**2a**] of this report with isolation of (+)-**2a** by Oh[1] and literature of (+)-**2a** by Sarpong[4]**:**

Oh's Isolation report (+)-Xiamycin C [(+)-**2a**] (^{1}H-NMR, 500 MHz, CD_3OD)[1]			
δ (ppm)	Int.	mult.	*J* (Hz)
8.00	1H	d	*J* = 8.0 Hz
7.94	1H	s	-
7.58	1H	s	-
7.38	1H	d	*J* = 8.0 Hz
7.31	1H	dd	*J* = 8.0, 8.0 Hz
7.10	1H	dd	*J* = 8.0, 8.0 Hz
4.91	1H	dd	*J* = 10.0, 7.5 Hz
4.08	1H	dd	*J* = 8.0, 6.5 Hz
2.62	1H	ddd	*J* = 13.0, 3.5, 3.5 Hz
2.20	1H	dd	*J* = 12.0, 2.0 Hz
1.97	1H	m	-
1.93	1H	m	-
1.89	2H	m	-
1.70	1H	m	-
1.38	3H	s	-
1.26	3H	s	-

Sarpong's report (+)-Xiamycin C [(+)-**2a**] (^{1}H-NMR, 600 MHz, CD_3OD)[4]			
δ (ppm)	Int.	mult.	*J* (Hz)
8.00	1H	d	*J* = 7.7 Hz
7.94	1H	s	-
7.58	1H	s	-
7.37	1H	d	*J* = 8.0 Hz
7.10	1H	d	*J* = 7.9, 8.0 Hz
4.89	1H	m	-
4.09	1H	dd	*J* = 8.0, 6.6 Hz
2.63	1H	ddd	*J* = 13.2, 3.4, 3.2 Hz

2.19	1H	d	*J* = 12.6 Hz
2.09 – 1.95	2H	m	-
1.93 – 1.82	3H	m	-
1.75 – 1.65	1H	m	-
1.38	3H	s	-
1.26	3H	s	-

This report (+)-Xiamycin C [(+)-**2a**] (^{1}H-NMR, 500 MHz, CD_3OD)			
δ (ppm)	Int.	mult.	*J* (Hz)
8.02	1H	d	*J* = 7.6 Hz
7.95	1H	s	-
7.60	1H	s	-
7.42 – 7.38	1H	m	-
7.33	1H	t	*J* = 7.5 Hz
7.12	1H	t	*J* = 7.5 Hz
4.91	1H	d	*J* = 8.5 Hz
4.10	1H	d	*J* = 8.2 Hz
2.64	1H	d	*J* = 12.8 Hz
2.22	1H	d	*J* = 12.3 Hz
2.01	2H	q	*J* = 11.9 Hz
1.93	3H	d	*J* = 12.1 Hz
1.73	1H	dd	*J* = 13.0, 7.5 Hz
1.40	3H	s	-
1.28	3H	s	-

Comparison of ^{13}C-NMR Data:

Oh's Isolation report (+)-Xiamycin C [(+)-**2a**] (^{13}C-NMR, 125 MHz, CD_3OD)[1]	Sarpong's report (+)-Xiamycin C [(+)-**2a**] (^{13}C-NMR, 176 MHz, CD_3OD)[4]	This report (+)-Xiamycin C [(+)-**2a**] (^{13}C-NMR, 125 MHz, CD_3OD)
182.0	180.4	180.3
142.4	142.3	142.4
141.7	142.1	141.6
140.2	140.1	140.2
137.6	137.9	137.5
126.4	126.3	126.4
124.4	124.5	124.4
124.1	124.0	124.2
120.8	120.8	120.8
119.4	119.3	119.4
116.3	116.3	116.4
111.6	111.5	111.6
110.1	110.1	110.1
76.3	76.8	76.3
72.2	72.3	72.2
54.8	55.1	54.9
46.1	46.0	46.1
39.2	39.2	39.2
39.0	39.0	39.0
33.2	33.3	33.2
28.5	28.4	28.6
26.8	26.9	26.8
11.9	12.7	11.8

Spectral Data

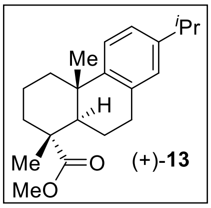

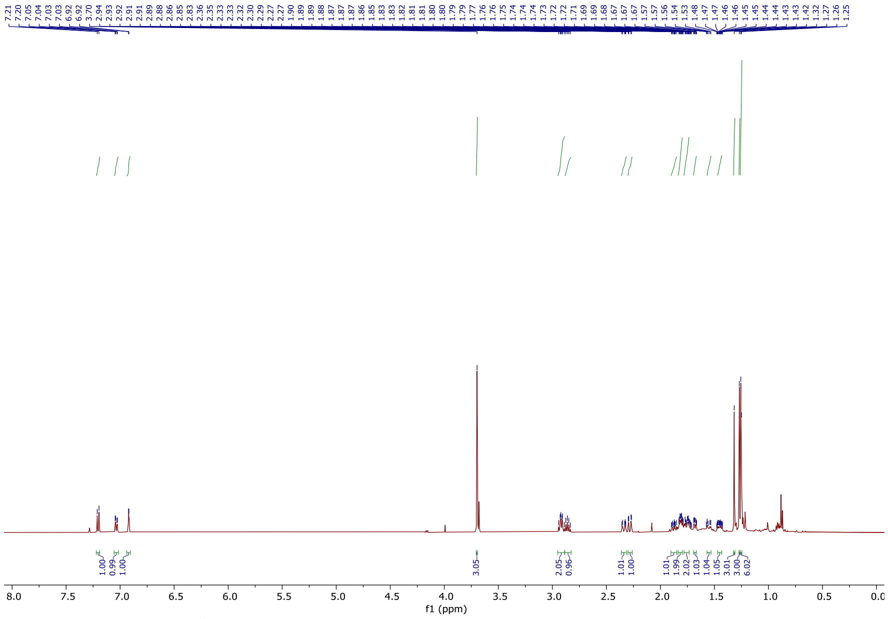

^{1}H NMR (500 MHz, $CDCl_3$) of (+)-**13**

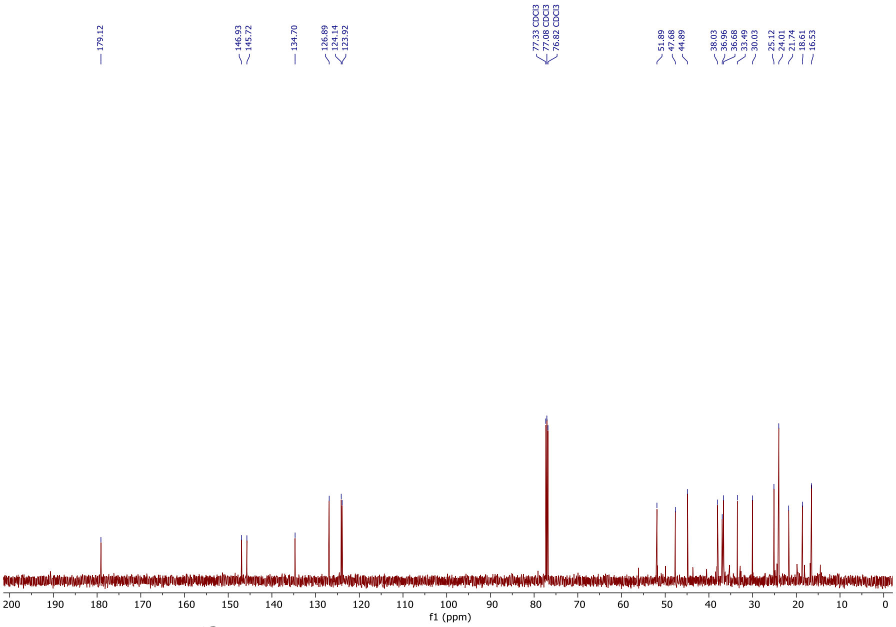

^{13}C NMR (125 MHz, $CDCl_3$) of (+)-**13**

Display Report

Analysis Info

Analysis Name	D:\Data\User data\2022\FEB\AB-MM_01_267.d	Acquisition Date	2/11/2022 11:36:44 AM
Method	Tune_pos_Mid.m	Operator	IISER Kolkata
Sample Name	AB-MM_01_267	Instrument	maXis impact 8282001.00127
Comment			

Acquisition Parameter

Source Type	ESI	Ion Polarity	Positive	Set Nebulizer	0.4 Bar
Focus	Active	Set Capillary	3400 V	Set Dry Heater	200 °C
Scan Begin	50 m/z	Set End Plate Offset	-500 V	Set Dry Gas	4.0 l/min
Scan End	3000 m/z	Set Charging Voltage	2000 V	Set Divert Valve	Source
		Set Corona	0 nA	Set APCI Heater	0 °C

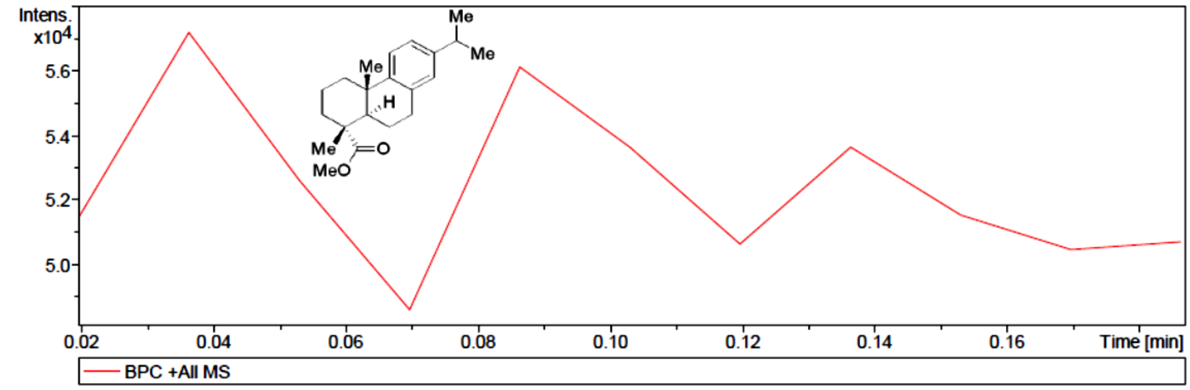

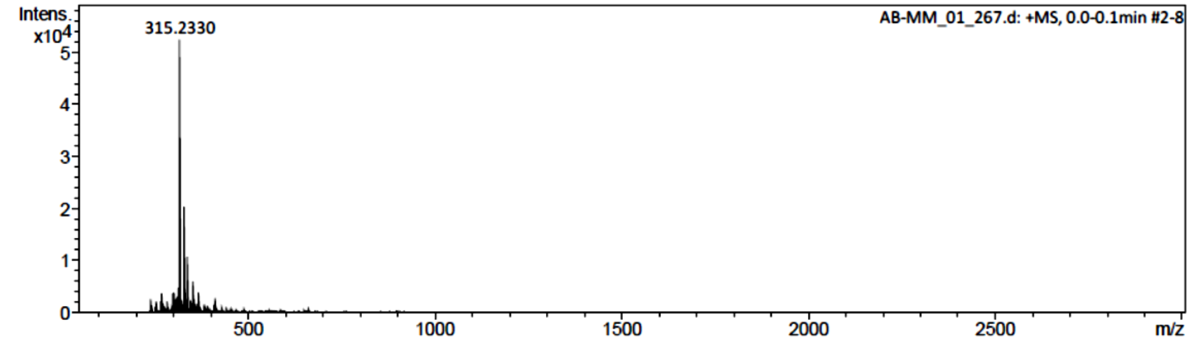

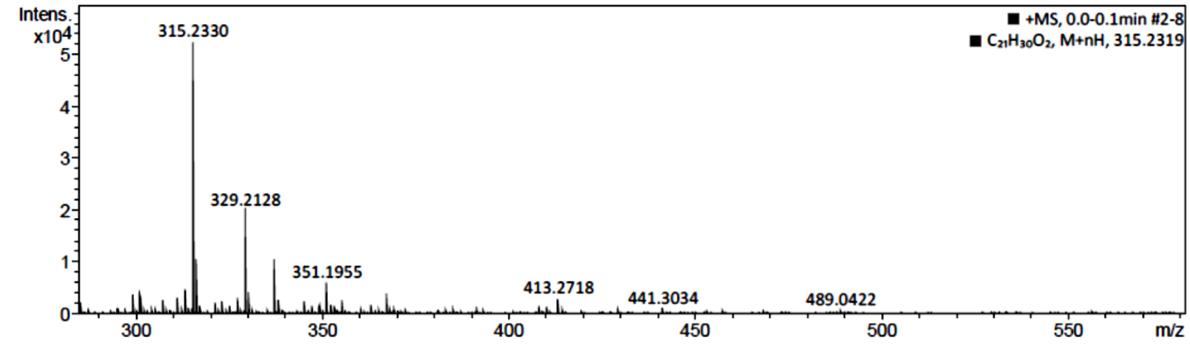

AB-MM_01_267.d

Bruker Compass DataAnalysis 4.1 printed: 2/11/2022 11:40:08 AM by: IISER Kolkata Page 1 of 1

HRMS data of (+)-**13**

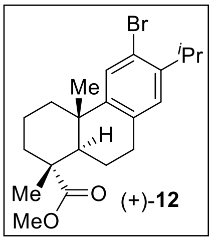

(+)-**12**

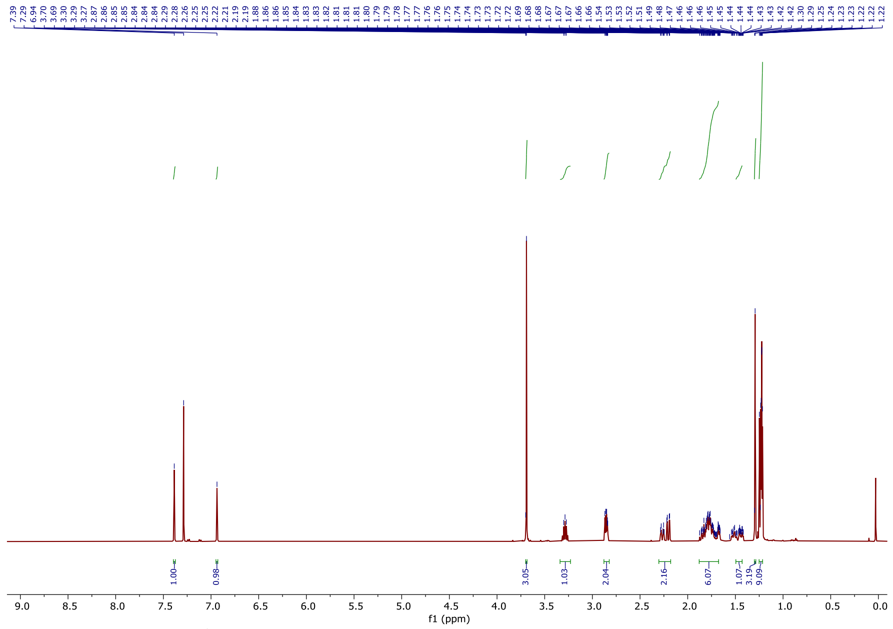

^{1}H NMR (500 MHz, $CDCl_3$) of (+)-**12**

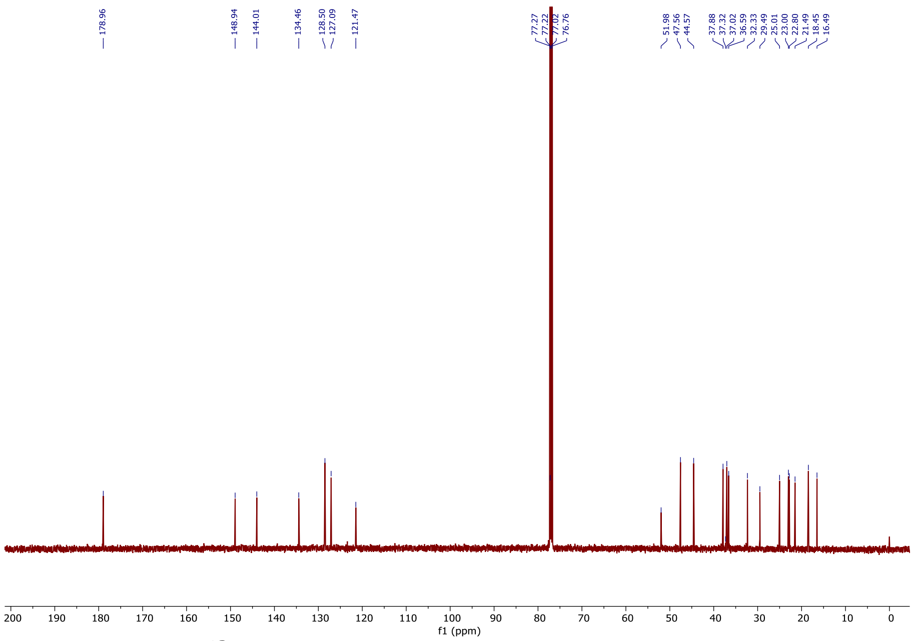

^{13}C NMR (125 MHz, $CDCl_3$) of (+)-**12**

Display Report

Analysis Info

Analysis Name	D:\Data\NEW USER DATA 2017\2019\APR\15A\Dr. A Bisai-RN-01-56.d	Acquisition Date	4/15/2019 11:57:00 AM
Method	tune mix_low.New.021117.m	Operator	RUCHI
Sample Name	RN-01-56	Instrument	micrOTOF-Q II 10330
Comment			

Acquisition Parameter

Source Type	ESI	Ion Polarity	Positive	Set Nebulizer	0.4 Bar
Focus	Not active	Set Capillary	4500 V	Set Dry Heater	180 °C
Scan Begin	50 m/z	Set End Plate Offset	-500 V	Set Dry Gas	4.0 l/min
Scan End	3000 m/z	Set Collision Cell RF	500.0 Vpp	Set Divert Valve	Waste

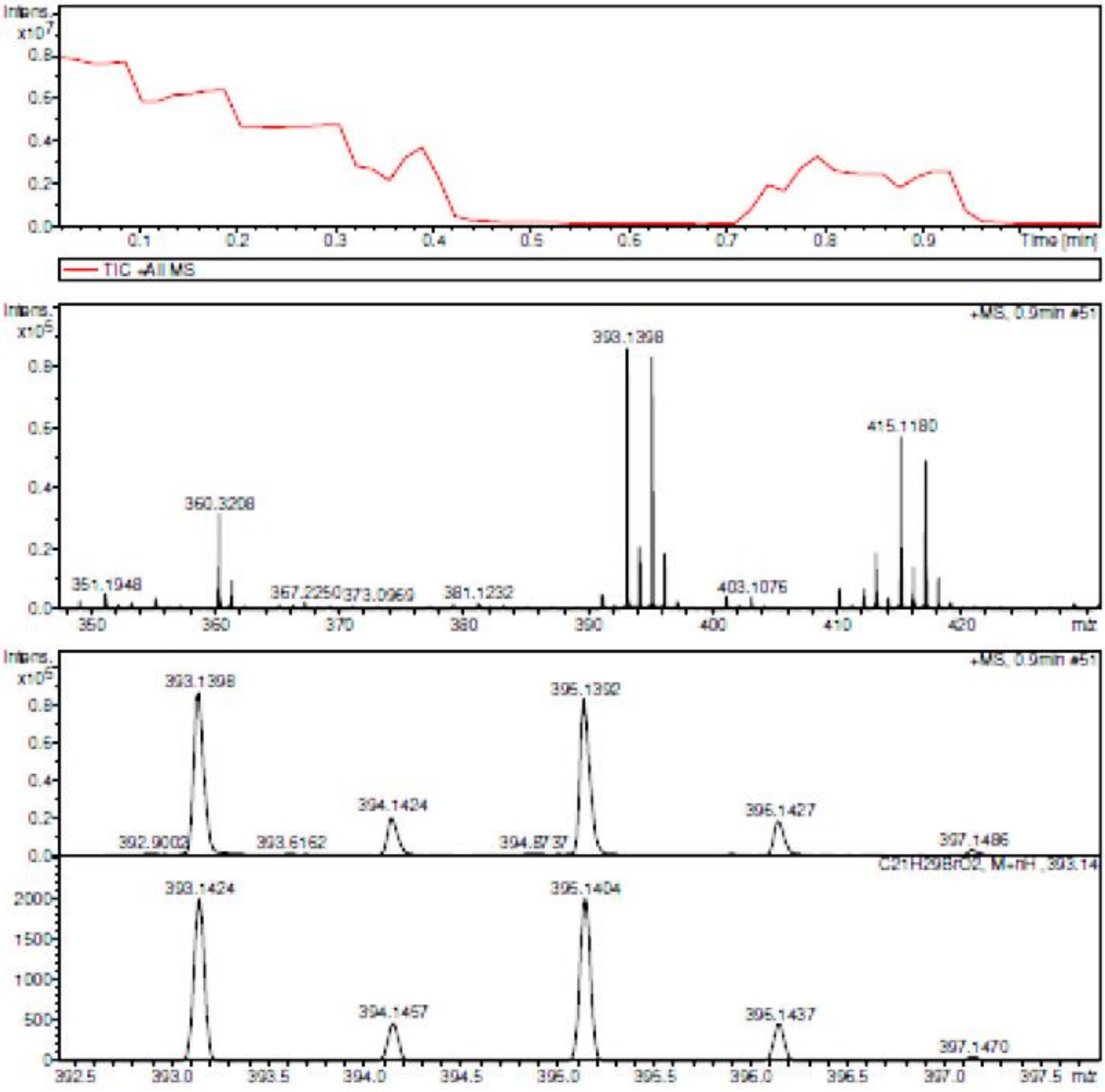

Bruker Compass DataAnalysis 4.0 printed: 4/15/2019 11:59:18 AM Page 1 of 1

HRMS data of (+)-**12**

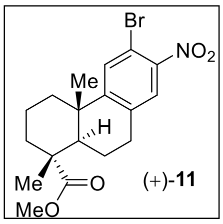

(+)-**11**

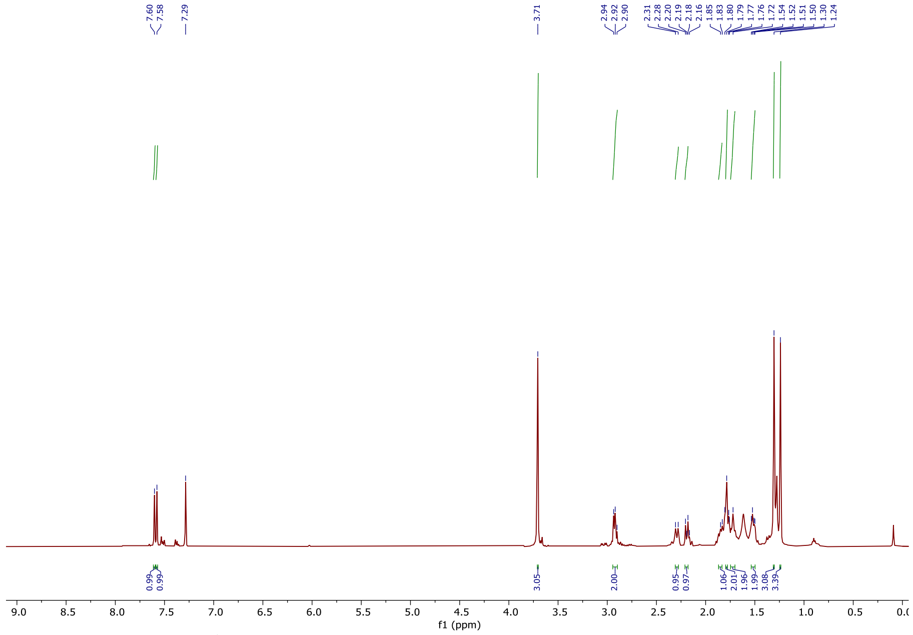

^{1}H NMR (400 MHz, $CDCl_3$) of (+)-**11**

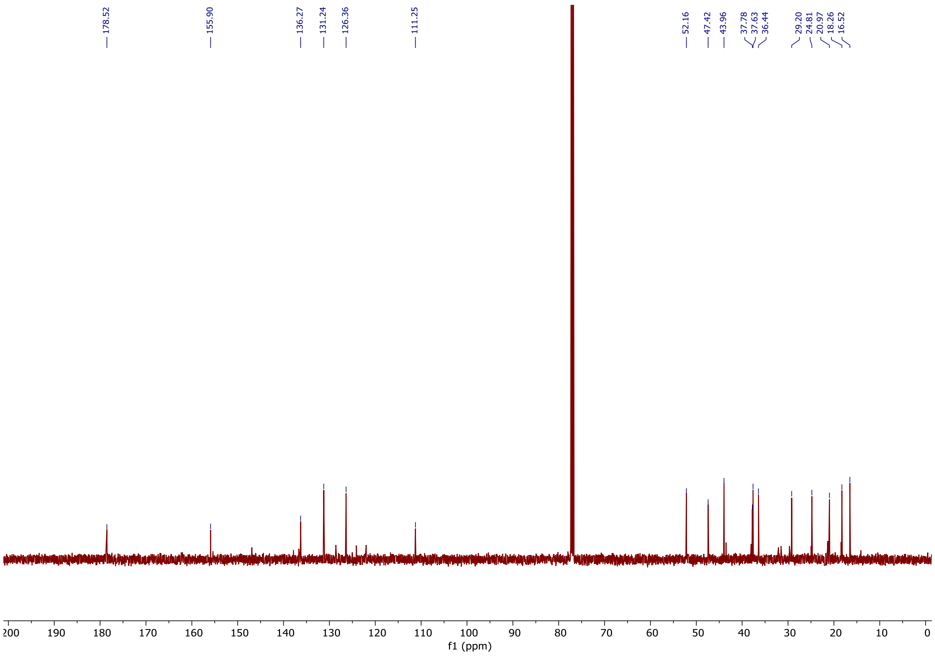

^{13}C NMR (125 MHz, $CDCl_3$) of (+)-**11**

Display Report

Analysis Info

Analysis Name	D:\Data\User data\2022\JAN\ab_sk_115.d	Acquisition Date	1/19/2022 1:07:32 PM
Method	Tune_pos_Standard.m	Operator	IISER Kolkata
Sample Name	ab_sk_115	Instrument	maXis impact 8282001.00127
Comment			

Acquisition Parameter

Source Type	ESI	Ion Polarity	Positive	Set Nebulizer	0.5 Bar
Focus	Active	Set Capillary	3400 V	Set Dry Heater	200 °C
Scan Begin	50 m/z	Set End Plate Offset	-500 V	Set Dry Gas	4.0 l/min
Scan End	1000 m/z	Set Charging Voltage	2000 V	Set Divert Valve	Source
		Set Corona	0 nA	Set APCI Heater	0 °C

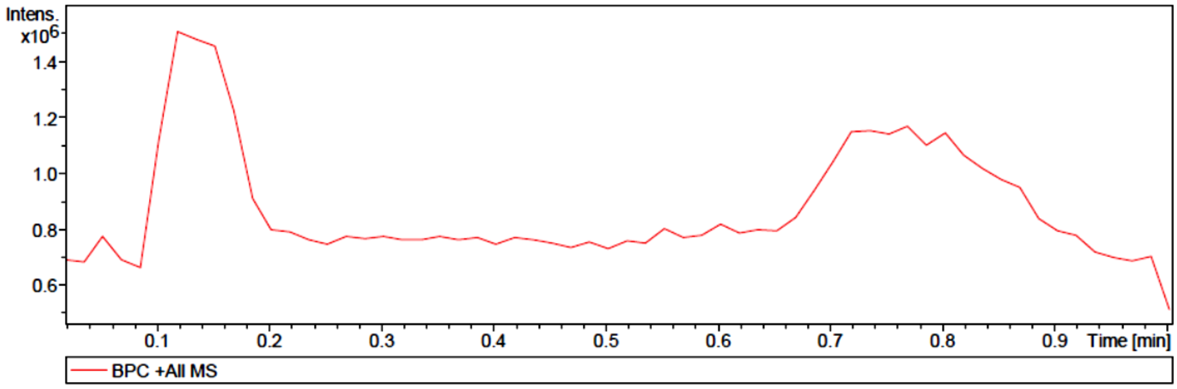

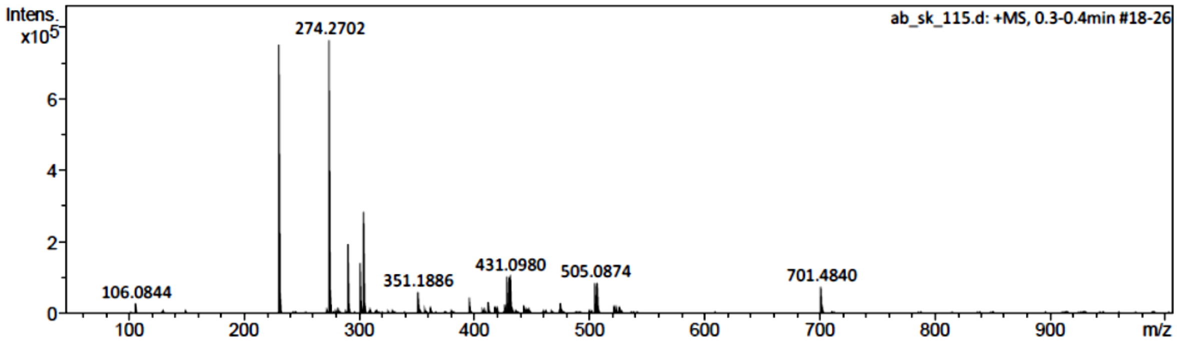

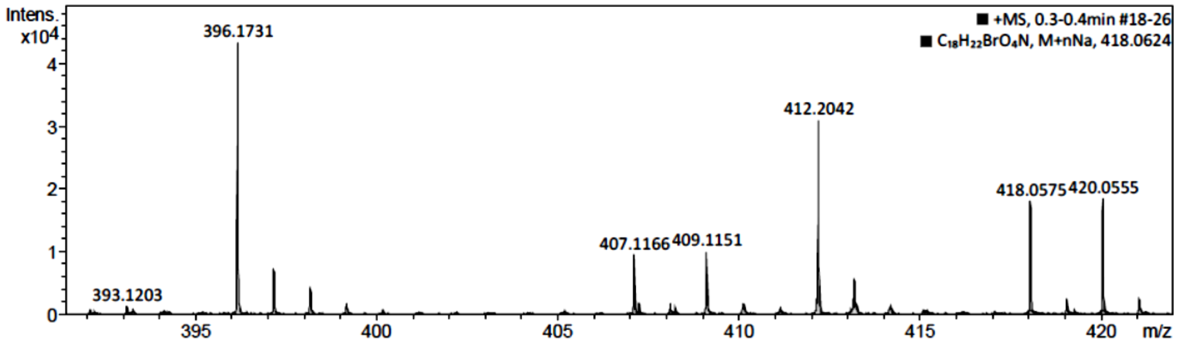

ab_sk_115.d			
Bruker Compass DataAnalysis 4.1	printed: 1/19/2022 1:11:09 PM	by: IISER Kolkata	Page 1 of 1

HRMS data of (+)-**11**

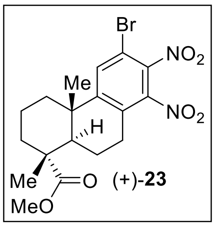

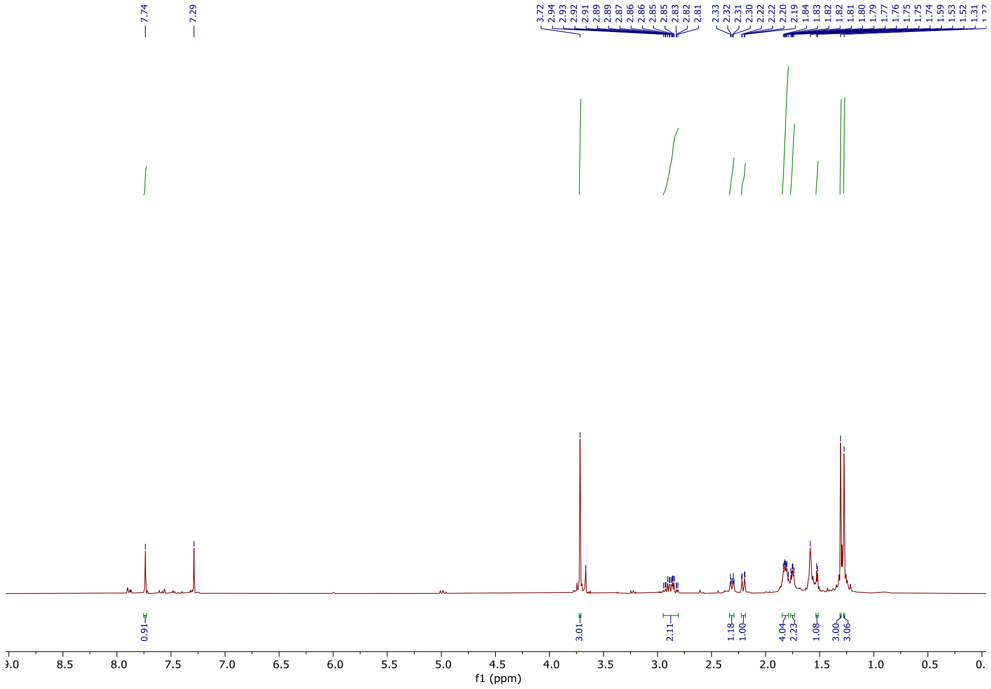

^{1}H NMR (500 MHz, $CDCl_3$) of (+)-**23**

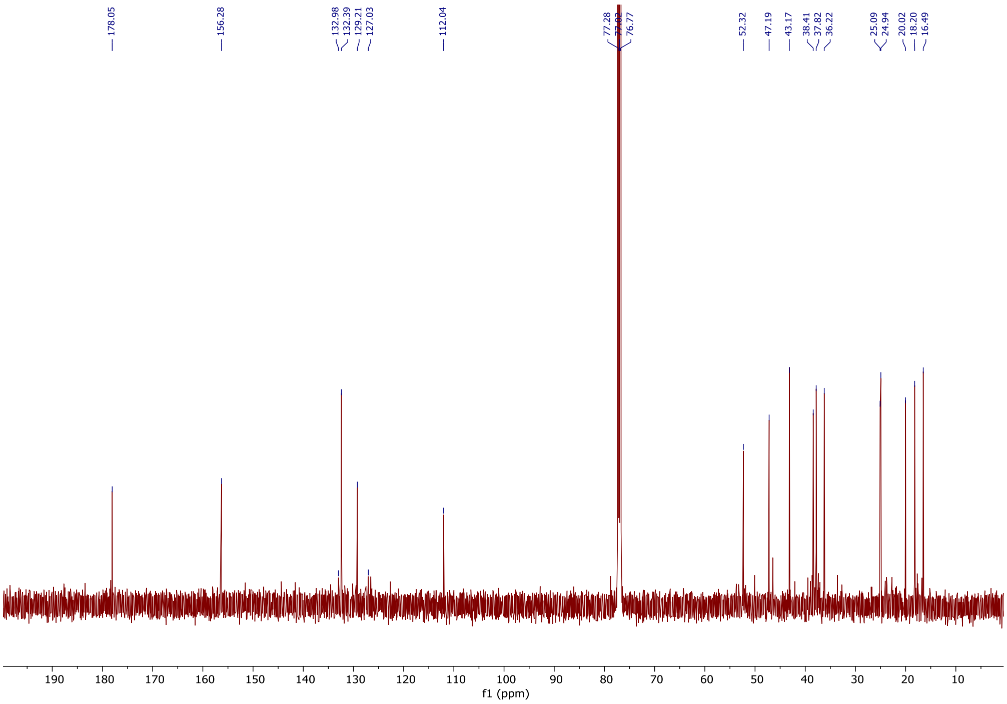

^{13}C NMR (125 MHz, $CDCl_3$) of (+)-**23**

Display Report

Analysis Info

Analysis Name	D:\Data\NEW USER DATA 2020\DEC-2020\17-dec\Prof.A.Bisai-AB-VR-03-352-P1.d	Acquisition Date	12/17/2020 2:43:32 PM
Method	tune_wide _APCI_23.06.m	Operator	RUCHI
Sample Name	AB-VR-03-352-P1	Instrument	micrOTOF-Q II 10330
Comment			

Acquisition Parameter

Source Type	Multi Mode	Ion Polarity	Positive	Set Nebulizer	2.0 Bar
Focus	Not active	Set Capillary	2500 V	Set Dry Heater	200 °C
Scan Begin	50 m/z	Set End Plate Offset	-500 V	Set Dry Gas	5.0 l/min
Scan End	3000 m/z	Set Collision Cell RF	600.0 Vpp	Set Divert Valve	Waste

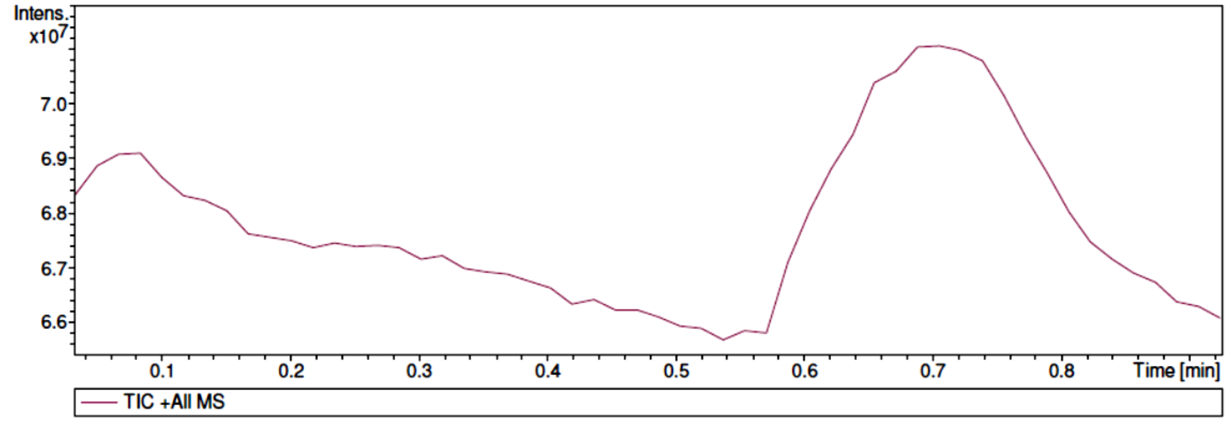

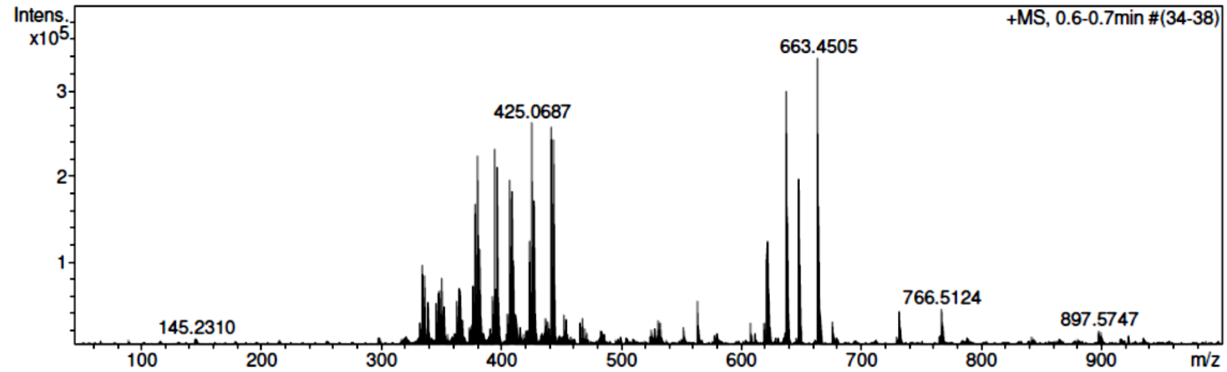

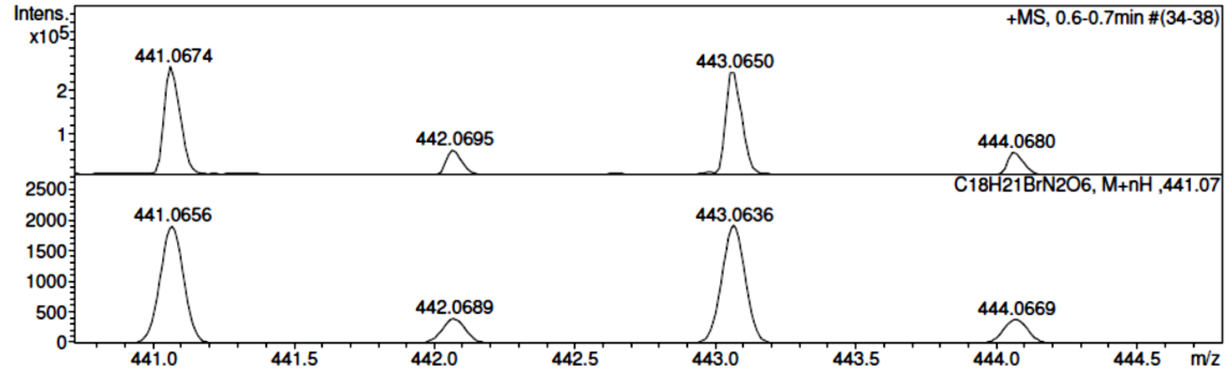

Bruker Compass DataAnalysis 4.0 printed: 12/17/2020 2:45:26 PM Page 1 of 1

HRMS data of (+)-**23**

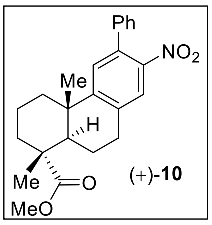

(+)-**10**

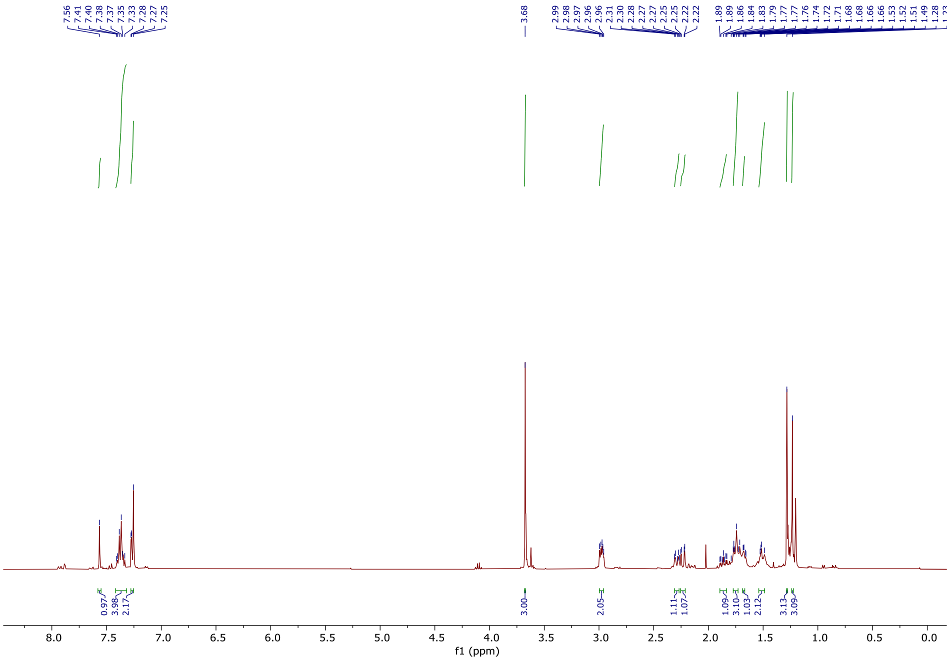

^{1}H NMR (500 MHz, $CDCl_3$) of (+)-**10**

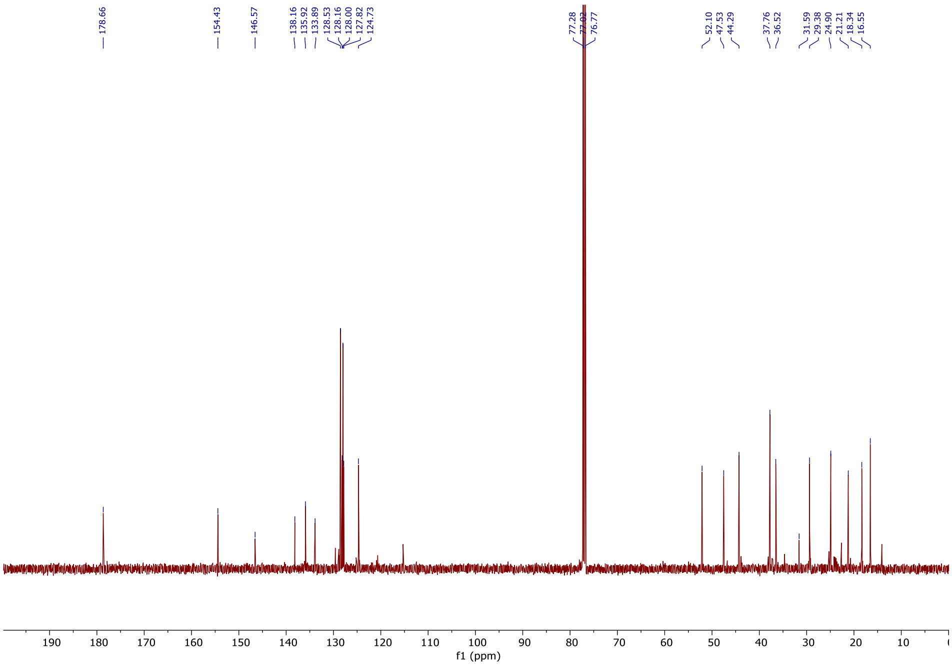

^{13}C NMR (125 MHz, $CDCl_3$) of (+)-**10**

Display Report

Analysis Info

Analysis Name	D:\Data\User data\2021\JAN\AB-DGJ-SK116.d	Acquisition Date	1/22/2021 11:39:55 AM
Method	Tune_pos_Standard.m	Operator	IISER Kolkata
Sample Name	AB-DGJ-SK116	Instrument	maXis impact 8282001.00127
Comment			

Acquisition Parameter

Source Type	ESI	Ion Polarity	Positive	Set Nebulizer	0.5 Bar
Focus	Active	Set Capillary	3400 V	Set Dry Heater	200 °C
Scan Begin	50 m/z	Set End Plate Offset	-500 V	Set Dry Gas	4.0 l/min
Scan End	1000 m/z	Set Charging Voltage	2000 V	Set Divert Valve	Source
		Set Corona	0 nA	Set APCI Heater	0 °C

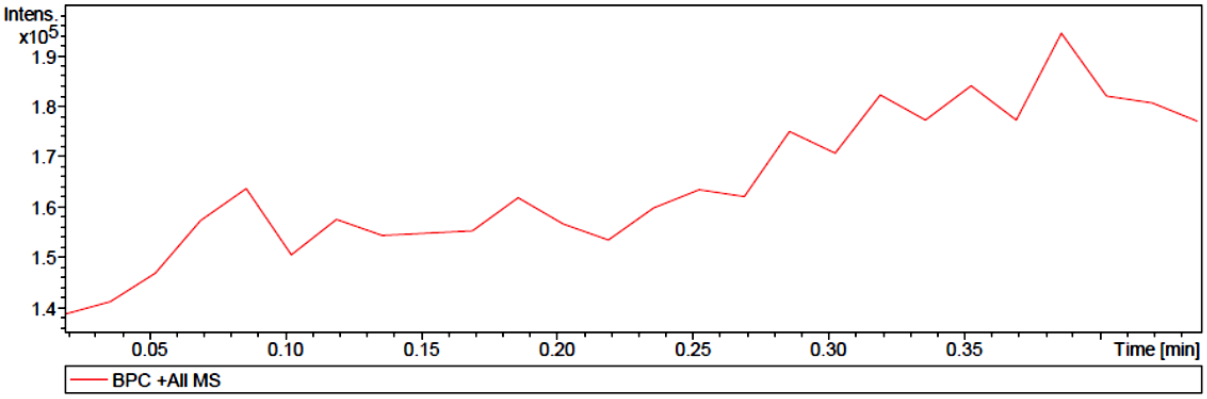

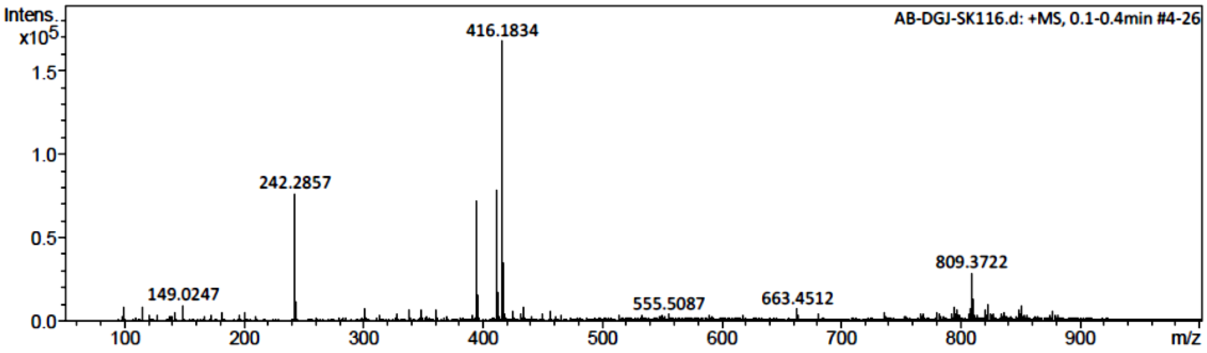

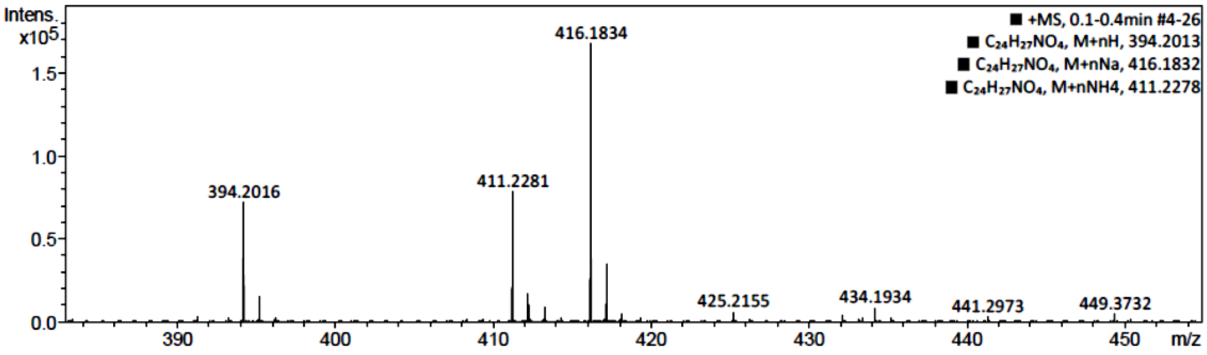

AB-DGJ-SK116.d

Bruker Compass DataAnalysis 4.1 printed: 1/22/2021 11:45:41 AM by: IISER Kolkata Page 1 of 1

HRMS data of (+)-**10**

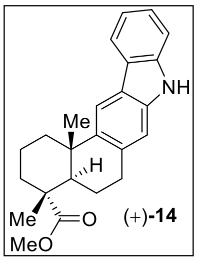

(+)-**14**

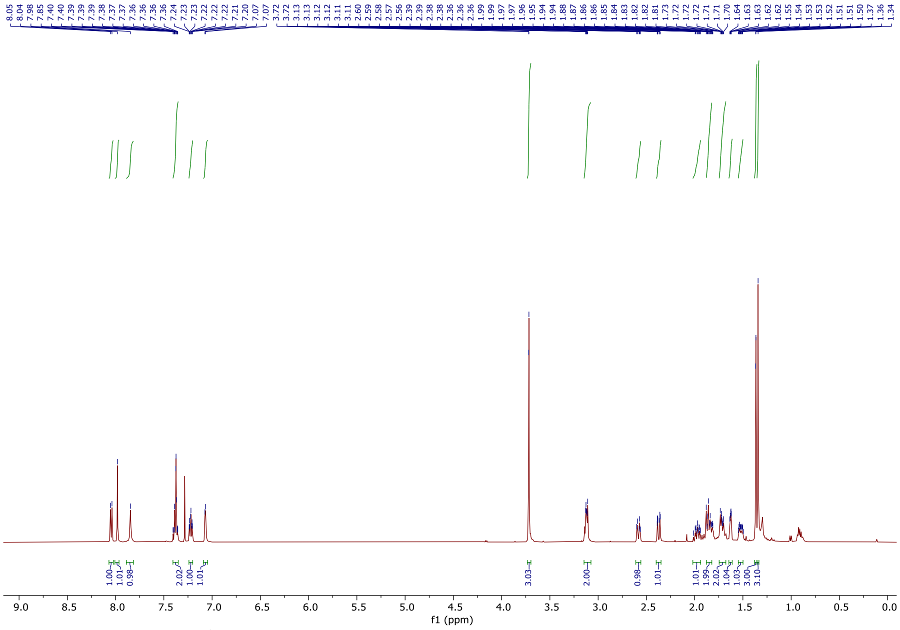

^{1}H NMR (500 MHz, $CDCl_3$) of (+)-**14**

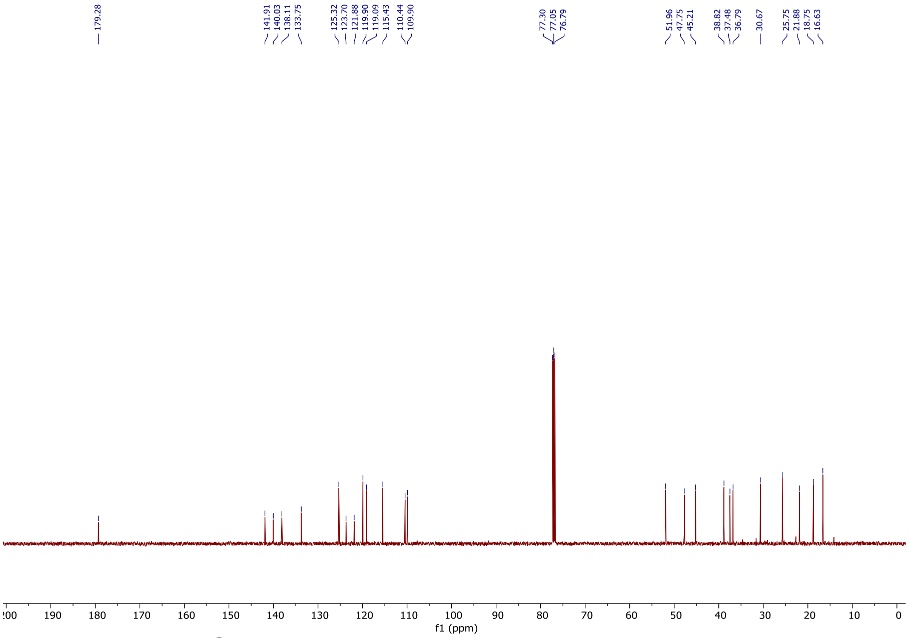

^{13}C NMR (125 MHz, $CDCl_3$) of (+)-**14**

Display Report

Analysis Info

Analysis Name	D:\Data\User data\2021\FEB\AB_MM_01_104.d	Acquisition Date	2/26/2021 11:45:11 AM
Method	Tune_pos_Mid.m	Operator	IISER Kolkata
Sample Name	AB_MM_01_104	Instrument	maXis impact 8282001.00127
Comment			

Acquisition Parameter

Source Type	ESI	Ion Polarity	Positive	Set Nebulizer	0.4 Bar
Focus	Active	Set Capillary	3400 V	Set Dry Heater	200 °C
Scan Begin	50 m/z	Set End Plate Offset	-500 V	Set Dry Gas	4.0 l/min
Scan End	3000 m/z	Set Charging Voltage	2000 V	Set Divert Valve	Source
		Set Corona	0 nA	Set APCI Heater	0 °C

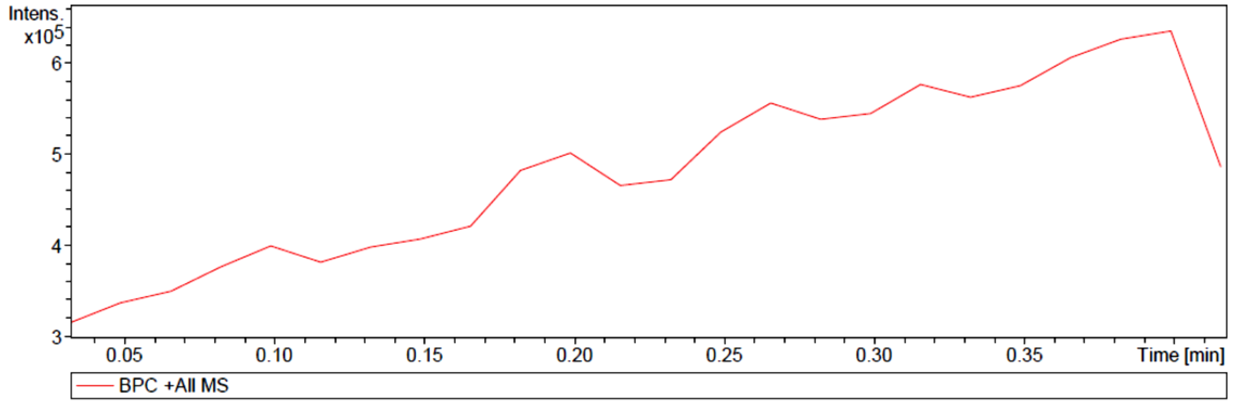

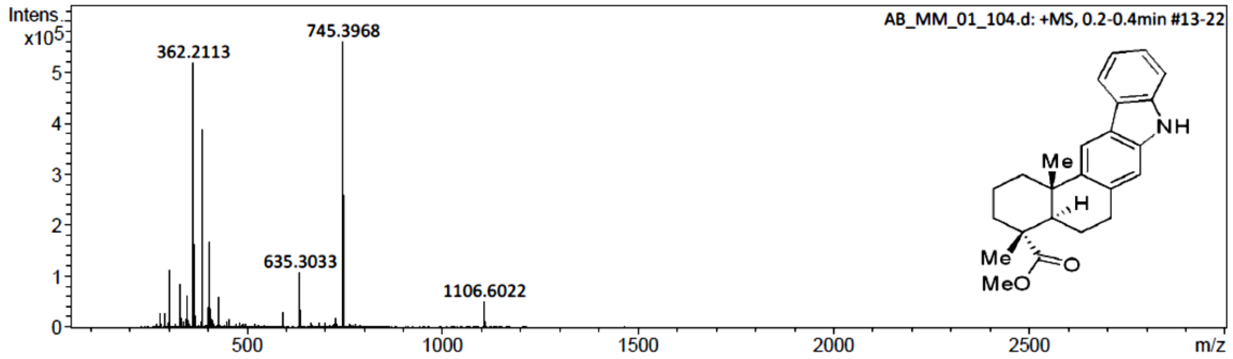

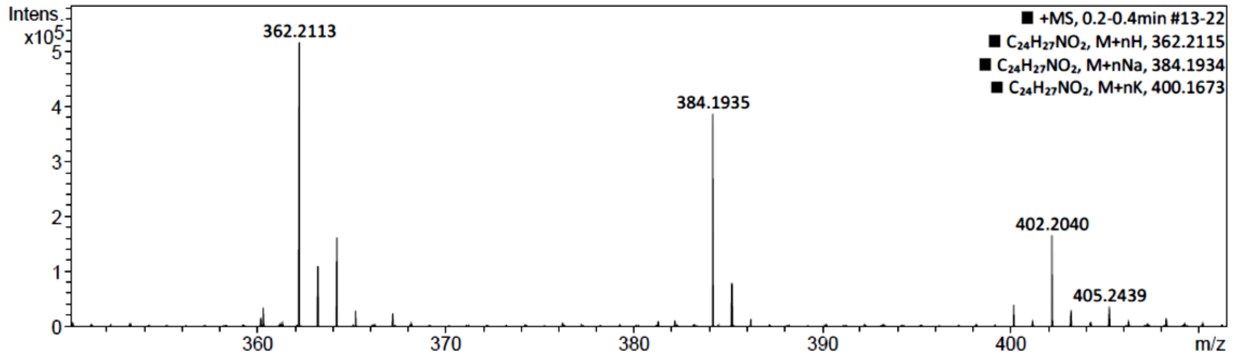

AB_MM_01_104.d

Bruker Compass DataAnalysis 4.1 printed: 2/26/2021 11:48:27 AM by: IISER Kolkata Page 1 of 1

HRMS data of (+)-**14**

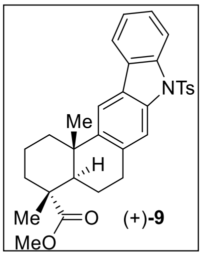

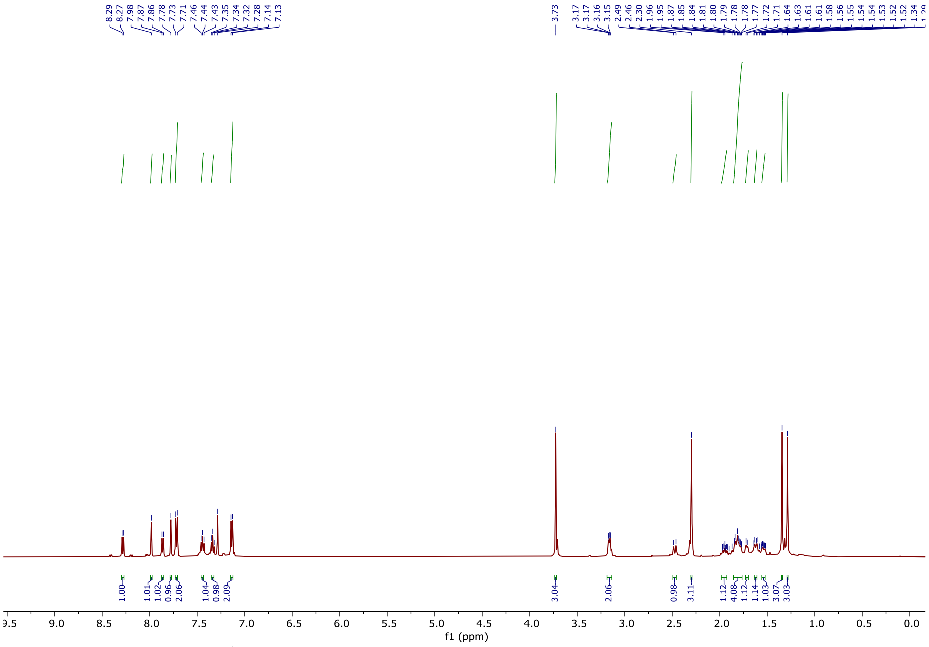

^{1}H NMR (500 MHz, $CDCl_3$) of (+)-**9**

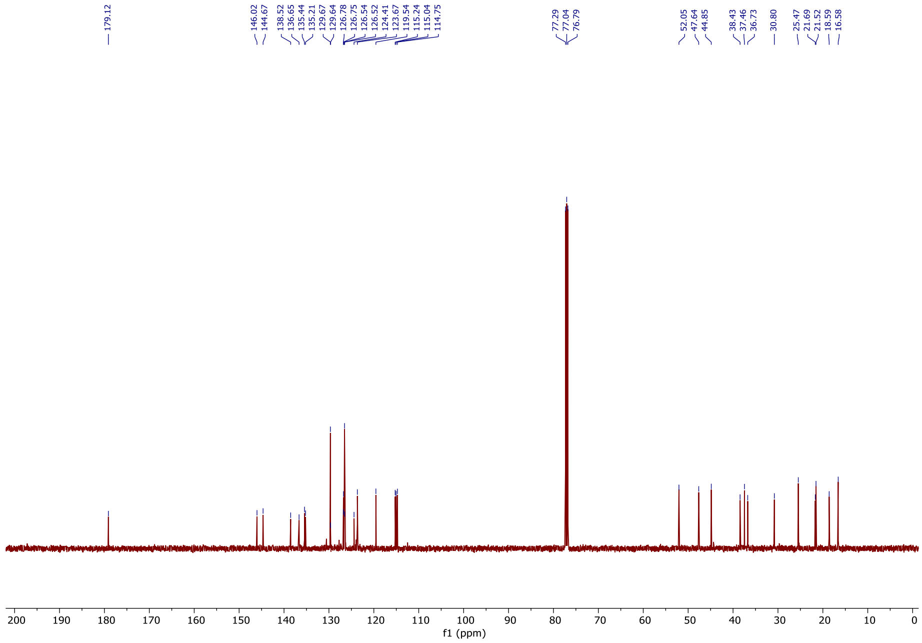

^{13}C NMR (125 MHz, $CDCl_3$) of (+)-**9**

Display Report

Analysis Info

Analysis Name	D:\Data\User data\2022\APRIL\AB_SK_122.d	Acquisition Date	4/5/2022 3:06:08 PM
Method	Tune_pos_Mid.m	Operator	IISER Kolkata
Sample Name	AB_SK_122	Instrument	maXis impact 8282001.00127
Comment			

Acquisition Parameter

Source Type	ESI	Ion Polarity	Positive	Set Nebulizer	0.4 Bar
Focus	Active	Set Capillary	3400 V	Set Dry Heater	200 °C
Scan Begin	50 m/z	Set End Plate Offset	-500 V	Set Dry Gas	4.0 l/min
Scan End	3000 m/z	Set Charging Voltage	2000 V	Set Divert Valve	Source
		Set Corona	0 nA	Set APCI Heater	0 °C

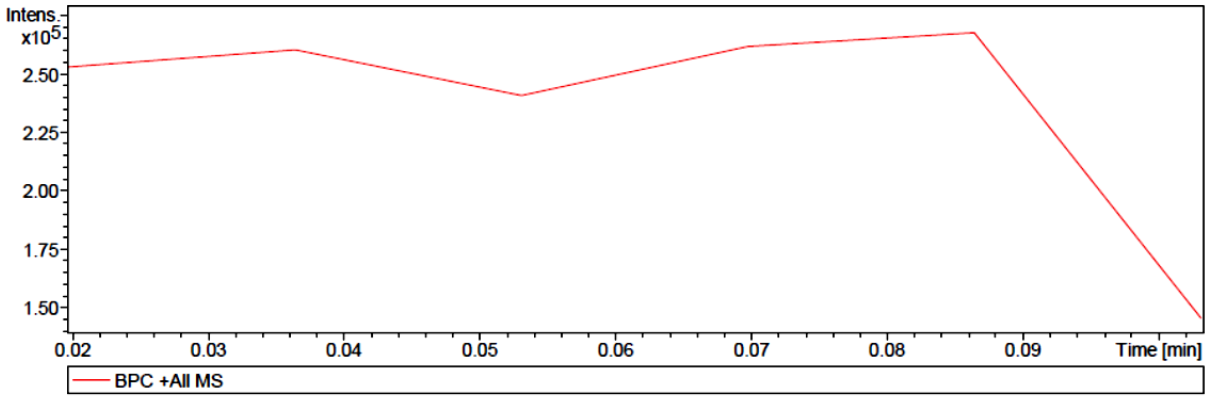

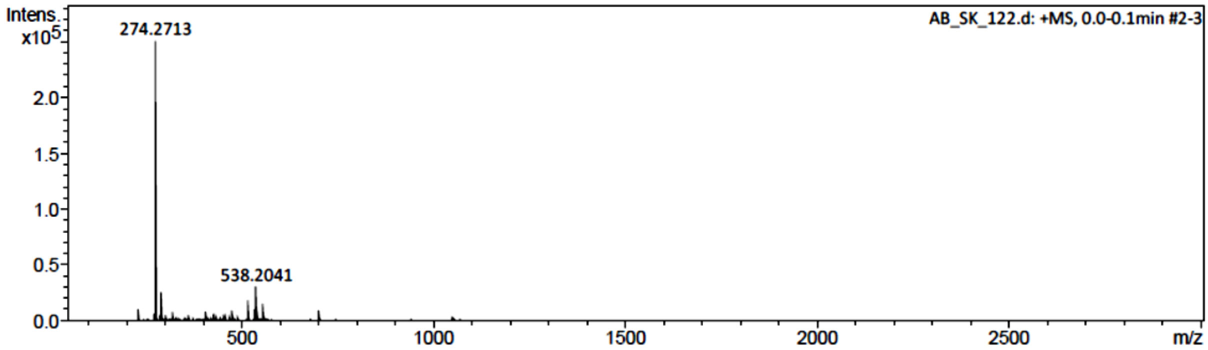

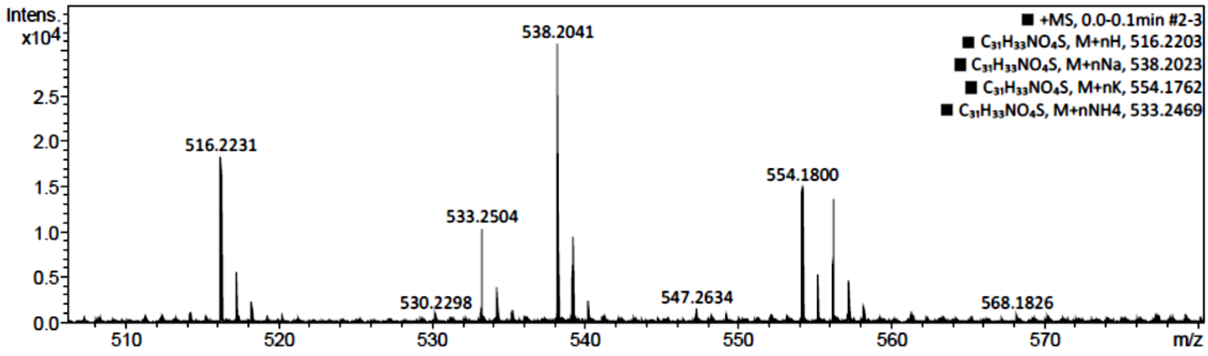

AB_SK_122.d

Bruker Compass DataAnalysis 4.1 printed: 4/5/2022 3:15:35 PM by: IISER Kolkata Page 1 of 1

HRMS data of (+)-**9**

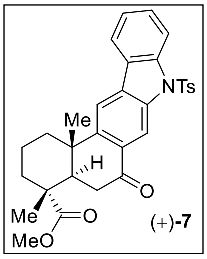

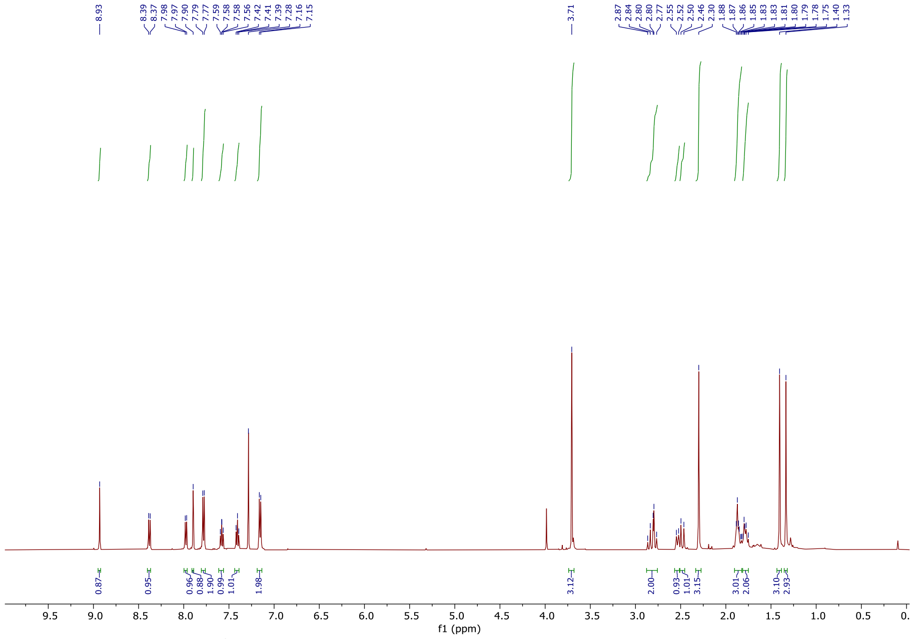

^{1}H NMR (500 MHz, $CDCl_3$) of (+)-**7**

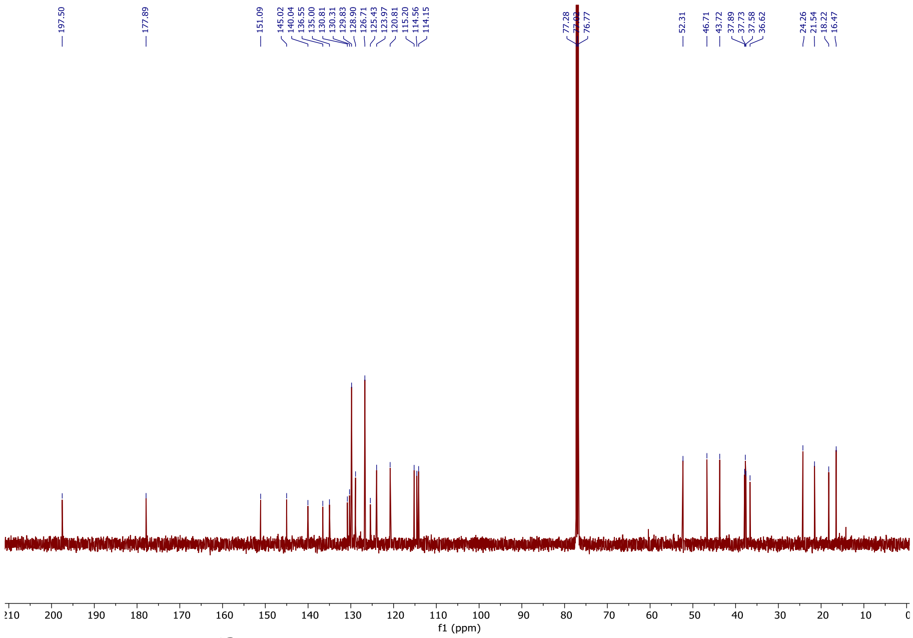

^{13}C NMR (125 MHz, $CDCl_3$) of (+)-**7**

Display Report

Analysis Info

Analysis Name	D:\Data\User data\2021\FEB\AB_MM_01_129.d	Acquisition Date	2/26/2021 12:01:38 PM
Method	Tune_pos_Mid.m	Operator	IISER Kolkata
Sample Name	AB_MM_01_129	Instrument	maXis impact 8282001.00127
Comment			

Acquisition Parameter

Source Type	ESI	Ion Polarity	Positive	Set Nebulizer	0.4 Bar
Focus	Active	Set Capillary	3400 V	Set Dry Heater	200 °C
Scan Begin	50 m/z	Set End Plate Offset	-500 V	Set Dry Gas	4.0 l/min
Scan End	3000 m/z	Set Charging Voltage	2000 V	Set Divert Valve	Source
		Set Corona	0 nA	Set APCI Heater	0 °C

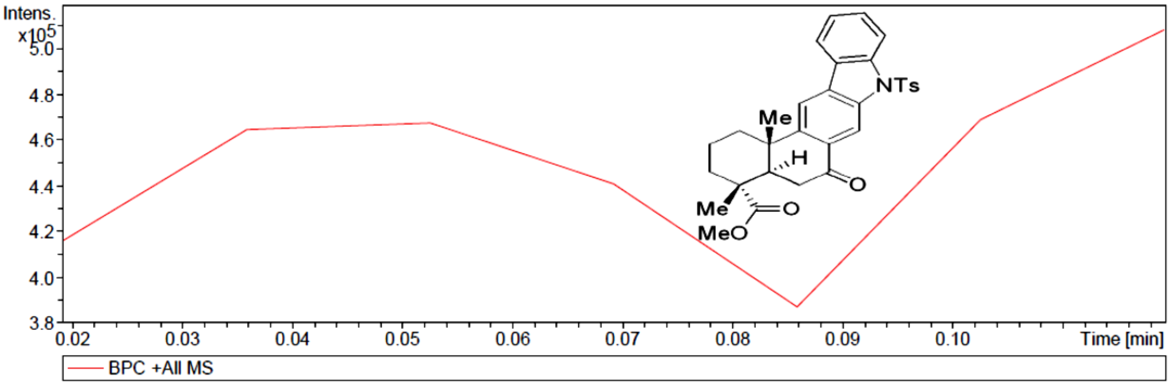

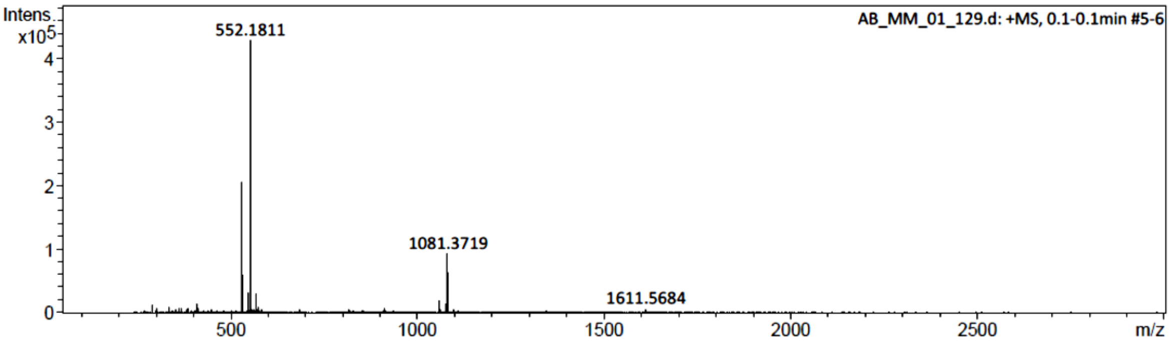

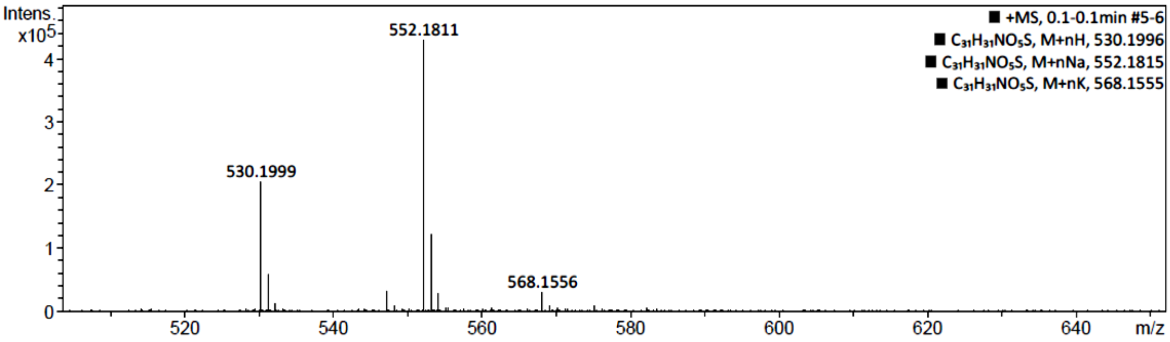

AB_MM_01_129.d

Bruker Compass DataAnalysis 4.1	printed: 2/26/2021 12:04:32 PM	by: IISER Kolkata	Page 1 of 1

HRMS data of (+)-**7**

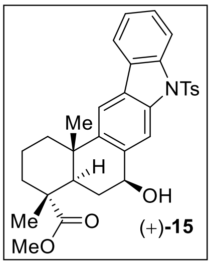

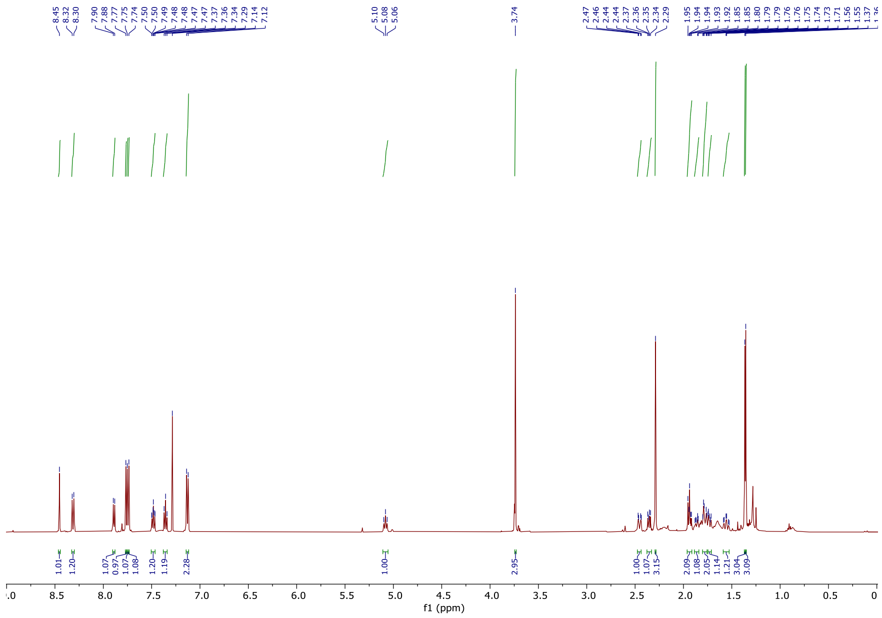

^{1}H NMR (500 MHz, $CDCl_3$) of (+)-**15**

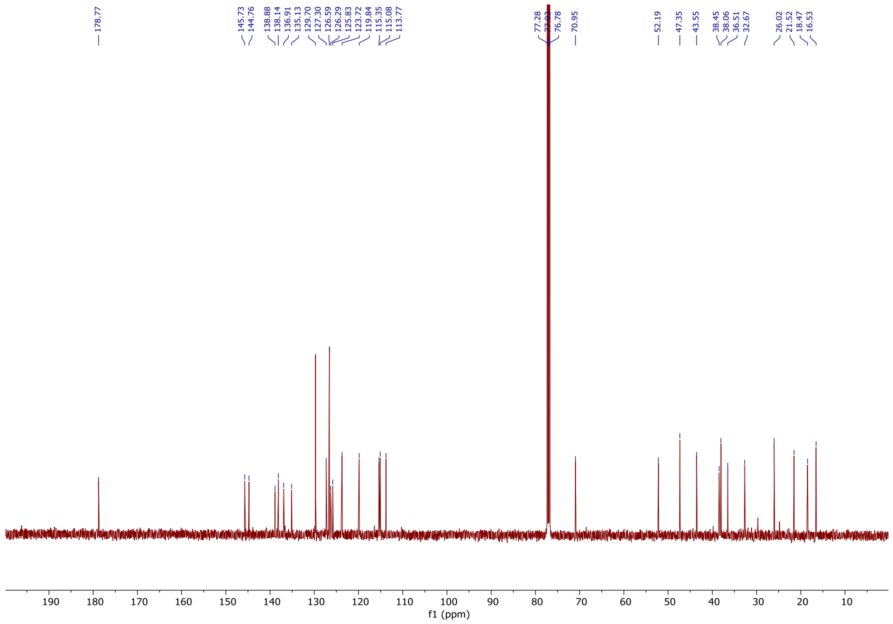

^{13}C NMR (125 MHz, $CDCl_3$) of (+)-**15**

Display Report

Analysis Info

Analysis Name	D:\Data\USER DATA 2022\JUNE2022\22-06-22\Prof.A.Bisai-ABMM01337.d	Acquisition Date	22-06-2022 15:15:57
Method	tune mix_low.New.021117.m	Operator	Bruker
Sample Name	ABMM01337	Instrument	micrOTOF-Q 10330
Comment			

Acquisition Parameter

Source Type	ESI	Ion Polarity	Positive	Set Nebulizer	0.4 Bar
Focus	Not active	Set Capillary	4600 V	Set Dry Heater	180 °C
Scan Begin	50 m/z	Set End Plate Offset	-500 V	Set Dry Gas	4.0 l/min
Scan End	3000 m/z	Set Collision Cell RF	100.0 Vpp	Set Divert Valve	Source

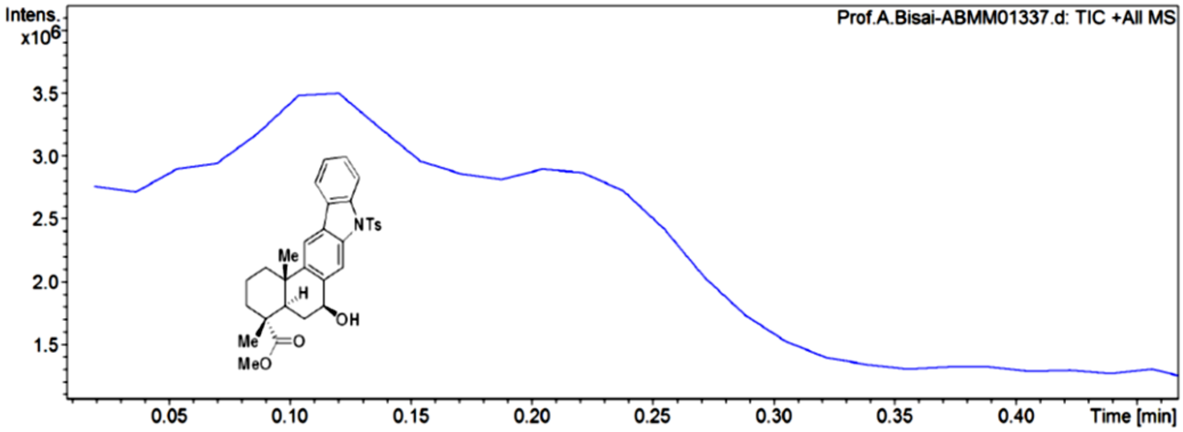

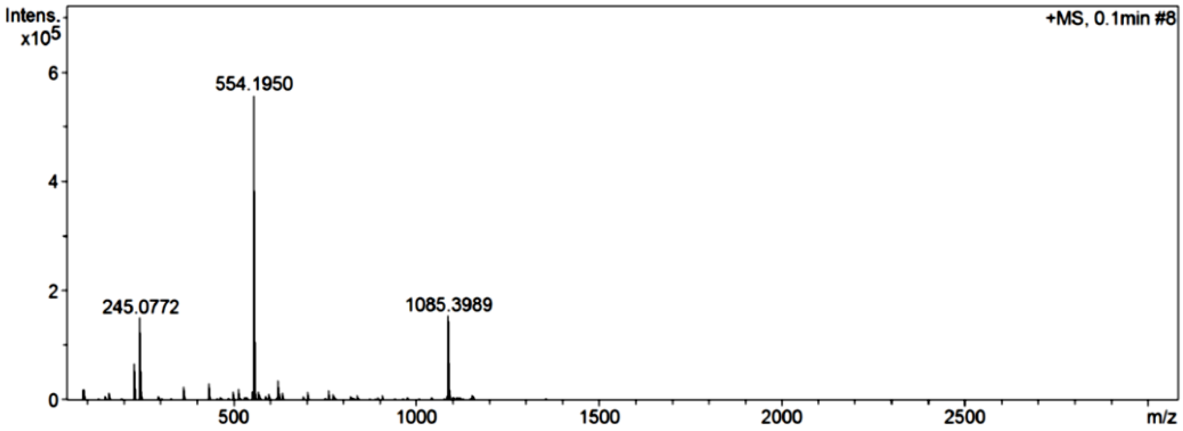

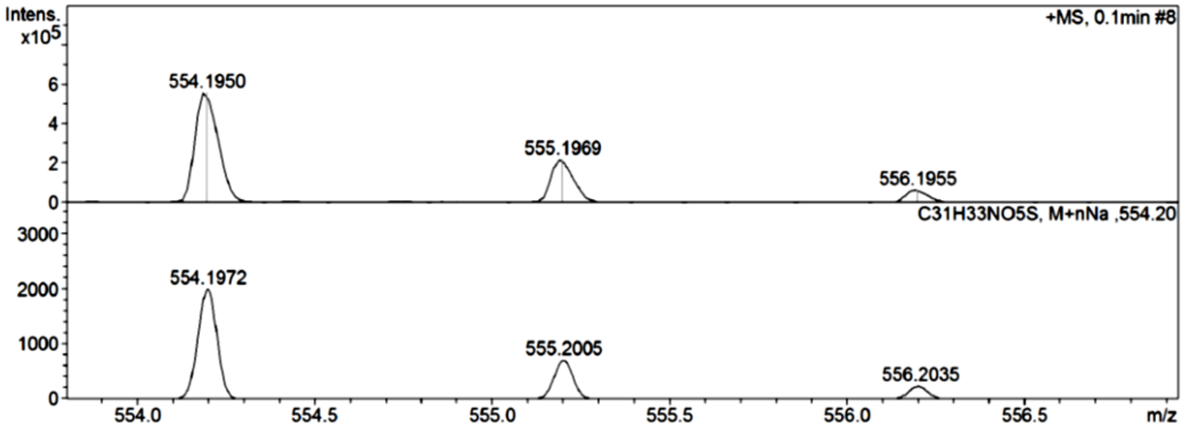

Bruker Compass DataAnalysis 4.0   printed: 22-06-2022 15:18:07   Page 1 of 1

HRMS data of (+)-**15**

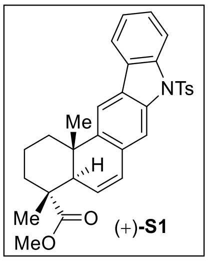

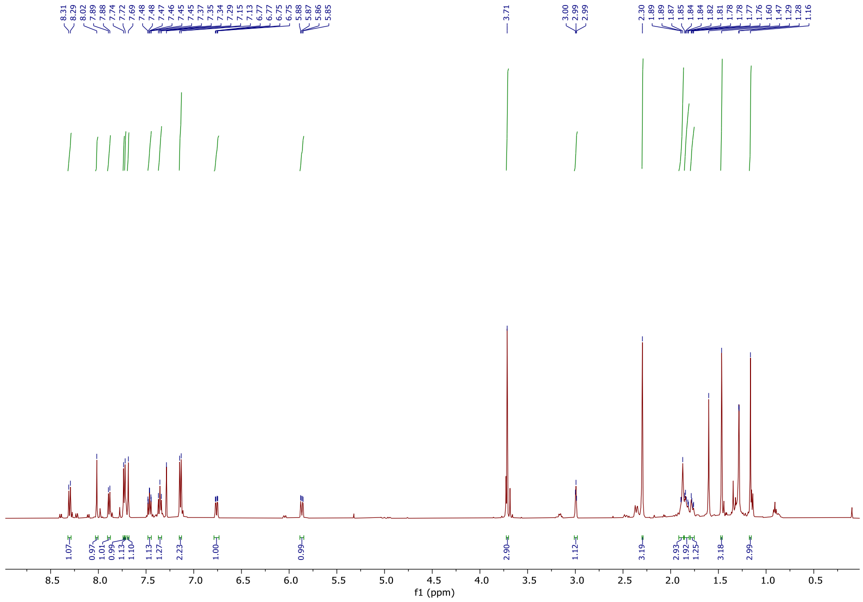

^{1}H NMR (500 MHz, $CDCl_3$) of (+)-**S1**

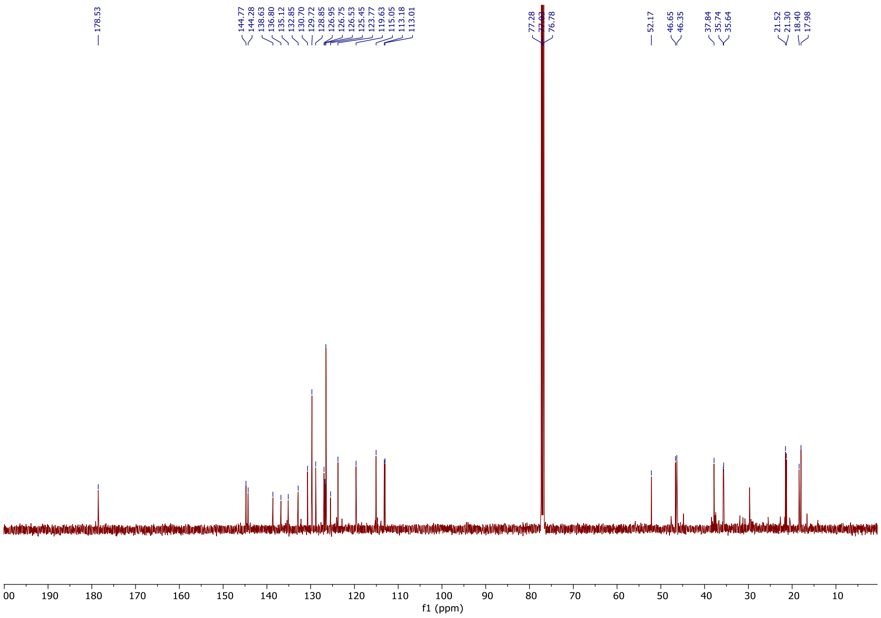

^{13}C NMR (125 MHz, $CDCl_3$) of (+)-**S1**

Display Report

Analysis Info

Analysis Name	D:\Data\USER DATA 2022\JUNE2022\22-06-22\Prof.A.Bisai-ABMM01340NP1.d	Acquisition Date	22-06-2022 15:19:17
Method	tune mix_low.New.021117.m	Operator	Bruker
Sample Name	ABMM01340NP1	Instrument	micrOTOF-Q 10330
Comment			

Acquisition Parameter

Source Type	ESI	Ion Polarity	Positive	Set Nebulizer	0.4 Bar
Focus	Not active	Set Capillary	4600 V	Set Dry Heater	180 °C
Scan Begin	50 m/z	Set End Plate Offset	-500 V	Set Dry Gas	4.0 l/min
Scan End	3000 m/z	Set Collision Cell RF	100.0 Vpp	Set Divert Valve	Source

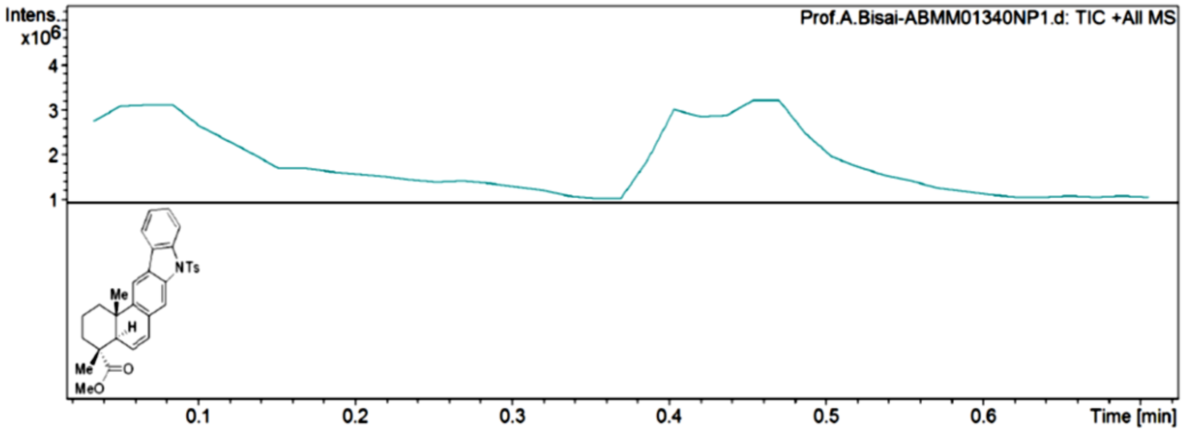

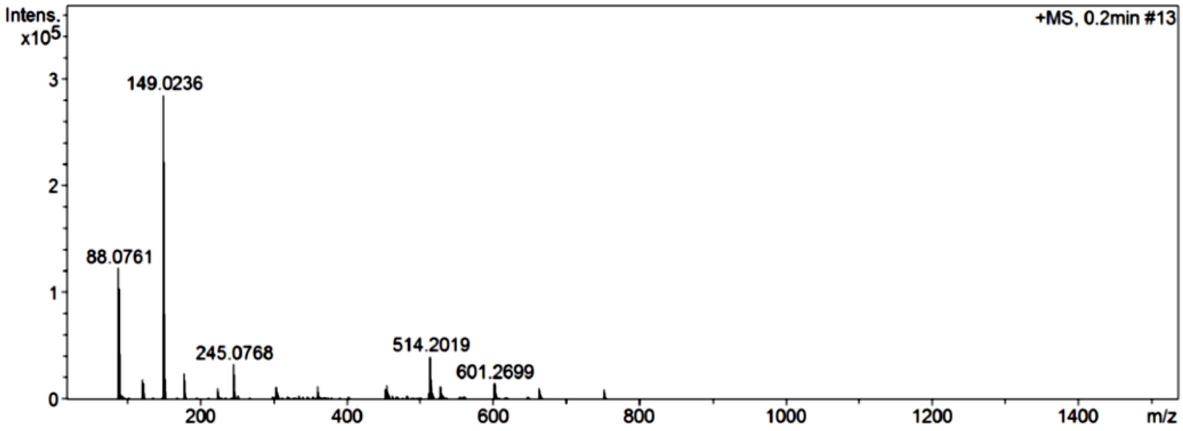

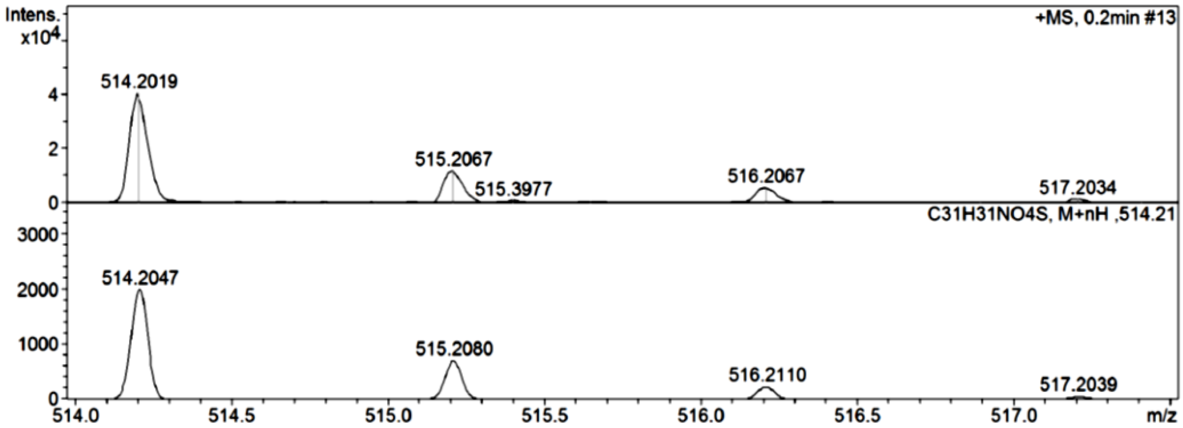

Bruker Compass DataAnalysis 4.0 printed: 22-06-2022 15:21:48 Page 1 of 1

HRMS data of (+)-**S1**

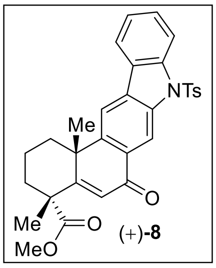

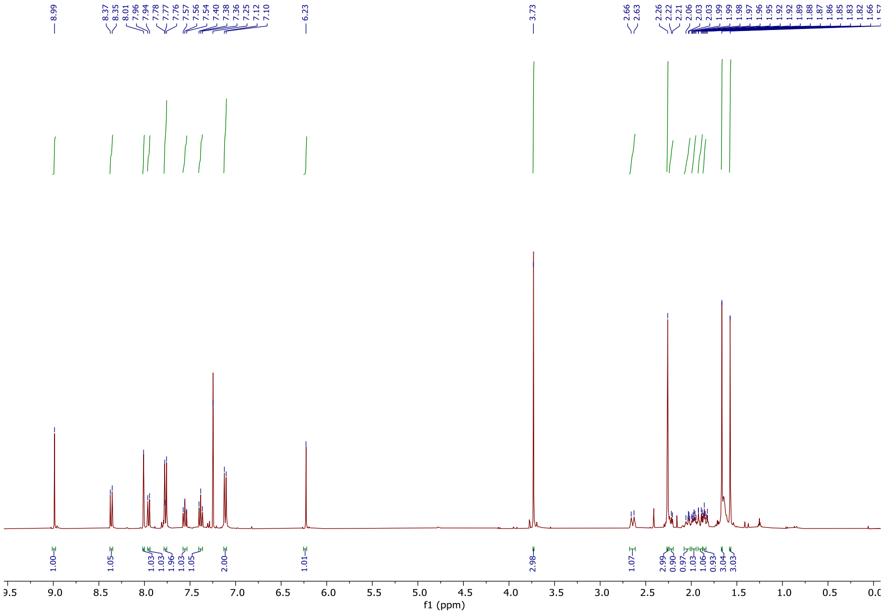

^{1}H NMR (500 MHz, $CDCl_3$) of (+)-**8**

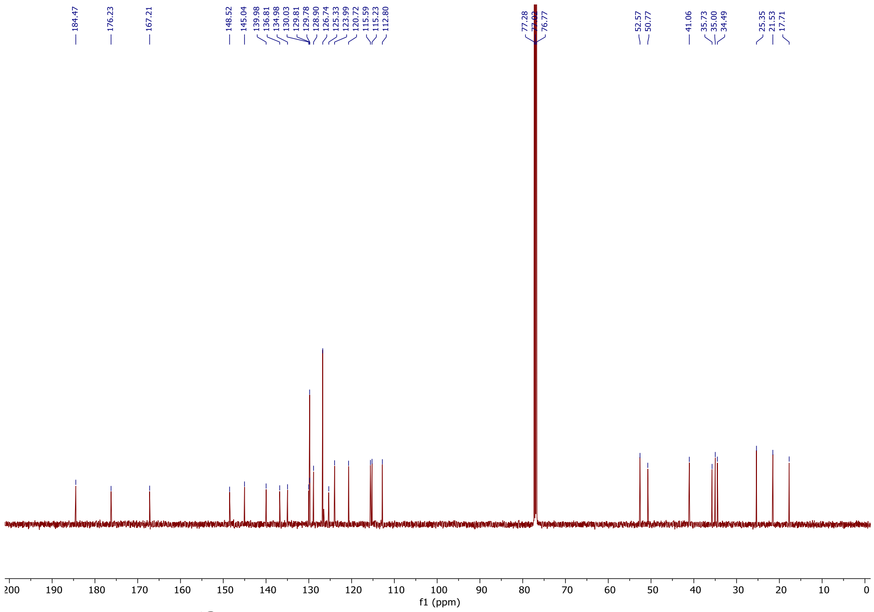

^{13}C NMR (125 MHz, $CDCl_3$) of (+)-**8**

Display Report

Analysis Info

Analysis Name	D:\Data\User data\2022\JAN\ab_mm_01_304.d	Acquisition Date	1/24/2022 1:09:47 PM
Method	Tune_pos_Standard.m	Operator	IISER Kolkata
Sample Name	ab_mm_01_304	Instrument	maXis impact 8282001.00127
Comment			

Acquisition Parameter

Source Type	ESI	Ion Polarity	Positive	Set Nebulizer	0.5 Bar
Focus	Active	Set Capillary	3400 V	Set Dry Heater	200 °C
Scan Begin	50 m/z	Set End Plate Offset	-500 V	Set Dry Gas	4.0 l/min
Scan End	1000 m/z	Set Charging Voltage	2000 V	Set Divert Valve	Source
		Set Corona	0 nA	Set APCI Heater	0 °C

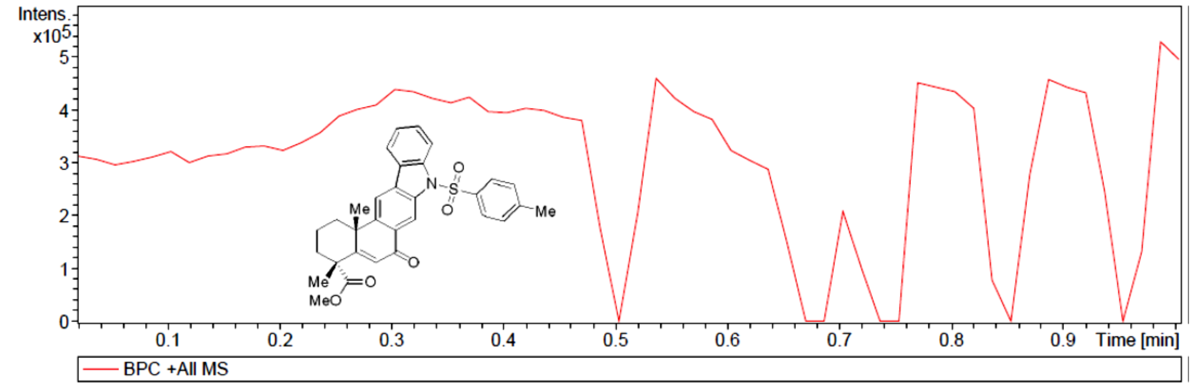

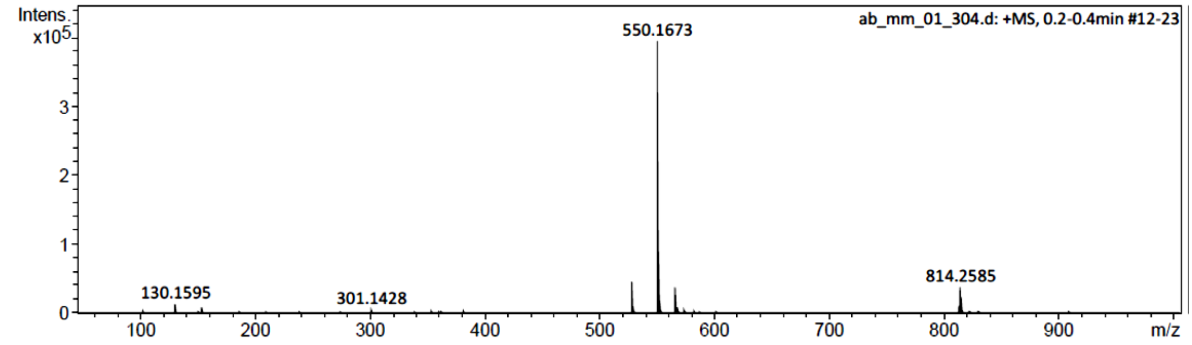

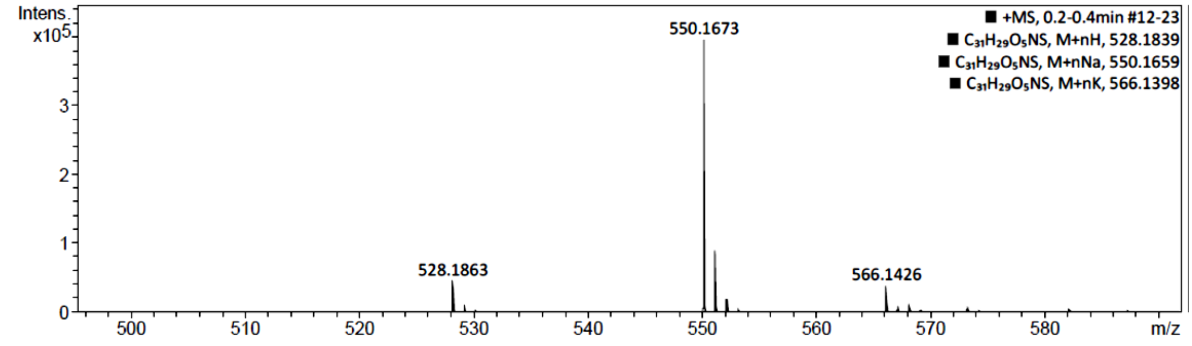

ab_mm_01_304.d

Bruker Compass DataAnalysis 4.1 printed: 1/24/2022 1:14:25 PM by: IISER Kolkata Page 1 of 1

HRMS data of (+)-**8**

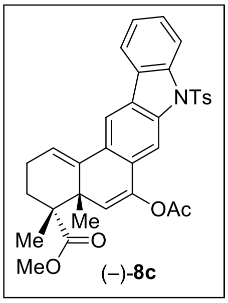

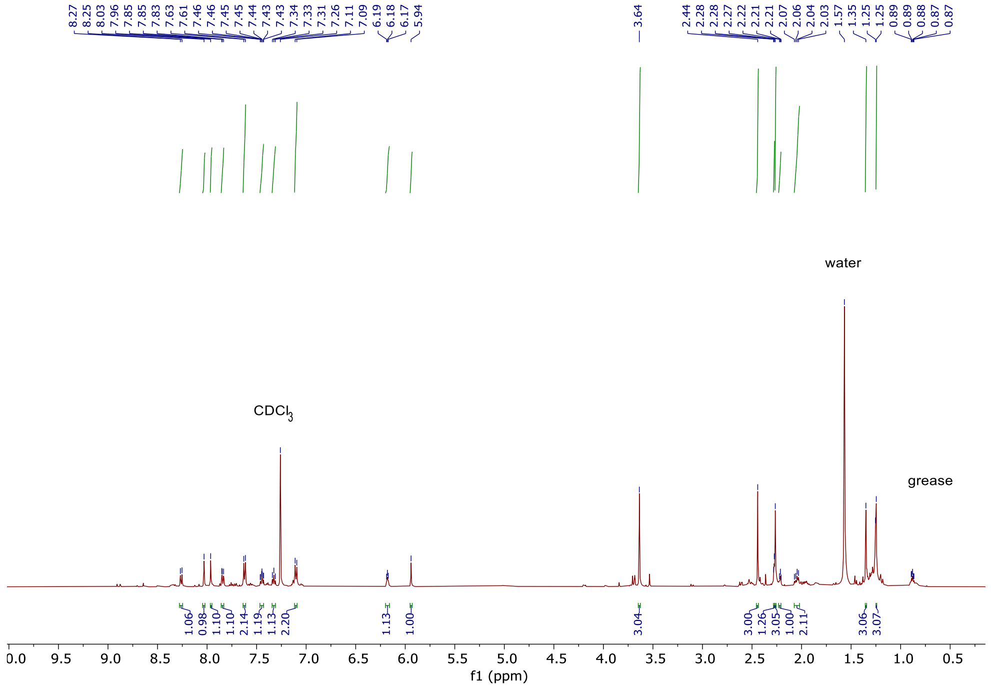

^{1}H NMR (500 MHz, $CDCl_3$) of (–)-**8c**

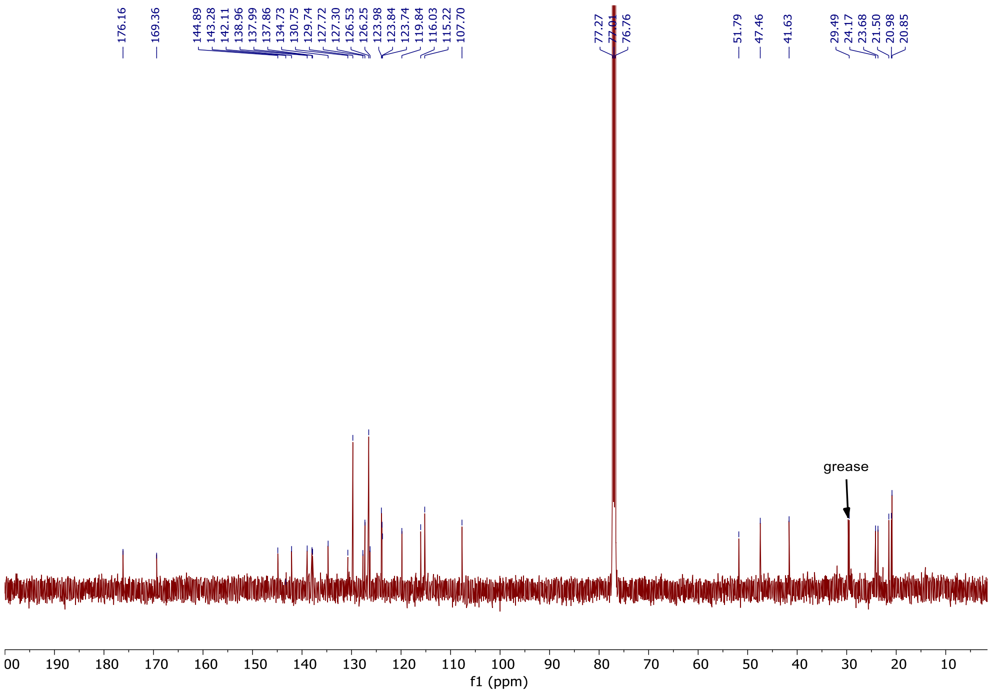

^{13}C NMR (125 MHz, $CDCl_3$) of (–)-**8c**

Display Report

Analysis Info

Analysis Name	D:\Data\User data\2022\AUGUST\ab-sov-mm01344np.d	Acquisition Date	8/5/2022 2:05:28 PM
Method	Tune_pos_Standard_July2022.m	Operator	IISER Kolkata
Sample Name	ab-sov-mm01344np	Instrument	maXis impact 8282001.00127
Comment			

Acquisition Parameter

Source Type	ESI	Ion Polarity	Positive	Set Nebulizer	0.4 Bar
Focus	Active	Set Capillary	4500 V	Set Dry Heater	200 °C
Scan Begin	50 m/z	Set End Plate Offset	-500 V	Set Dry Gas	4.0 l/min
Scan End	1000 m/z	Set Charging Voltage	2000 V	Set Divert Valve	Source
		Set Corona	0 nA	Set APCI Heater	0 °C

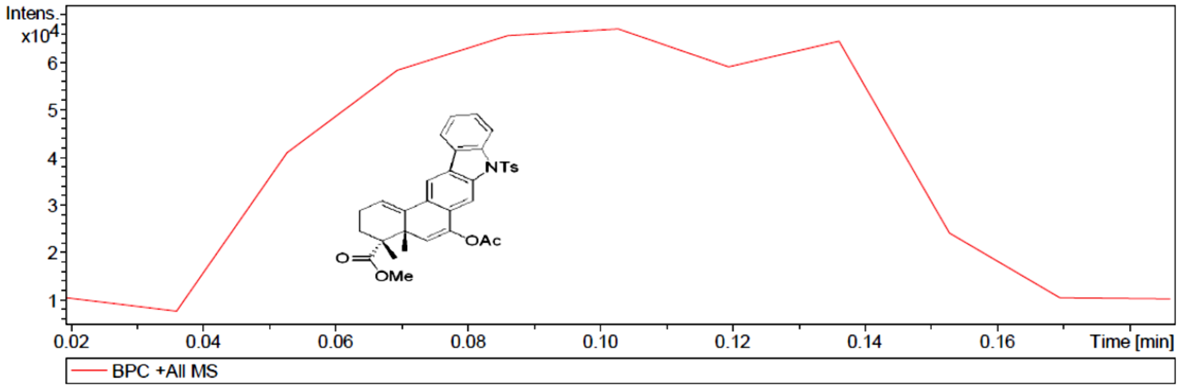

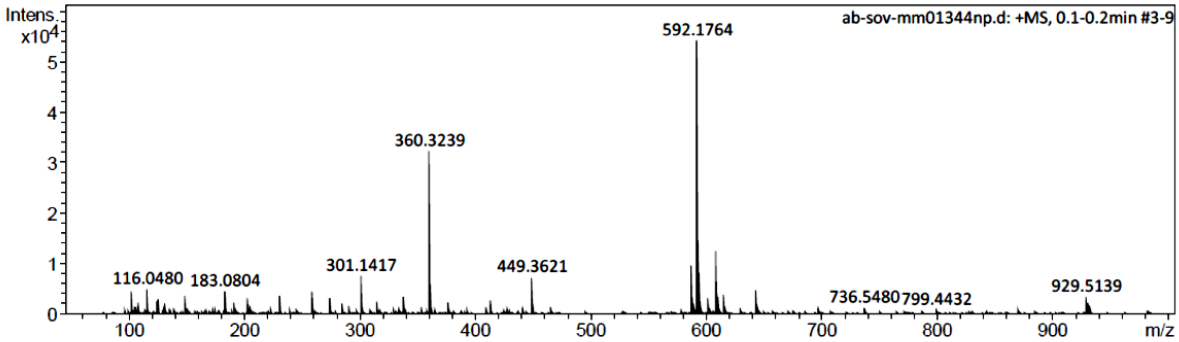

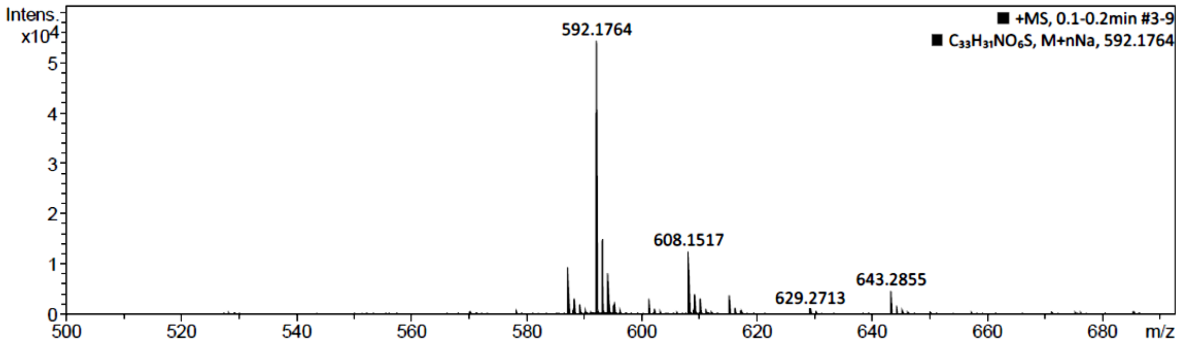

ab-sov-mm01344np.d

Bruker Compass DataAnalysis 4.1 printed: 8/5/2022 2:08:11 PM by: IISER Kolkata Page 1 of 1

HRMS data of (−)-**8c**

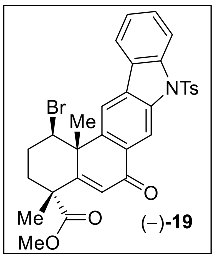

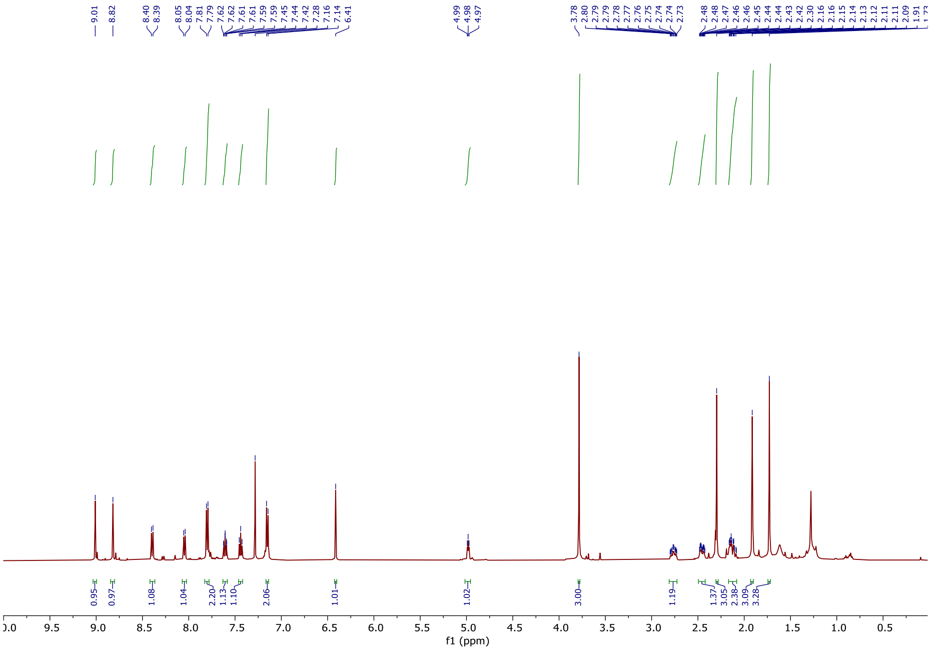

^{1}H NMR (500 MHz, $CDCl_3$) of (−)-**19**

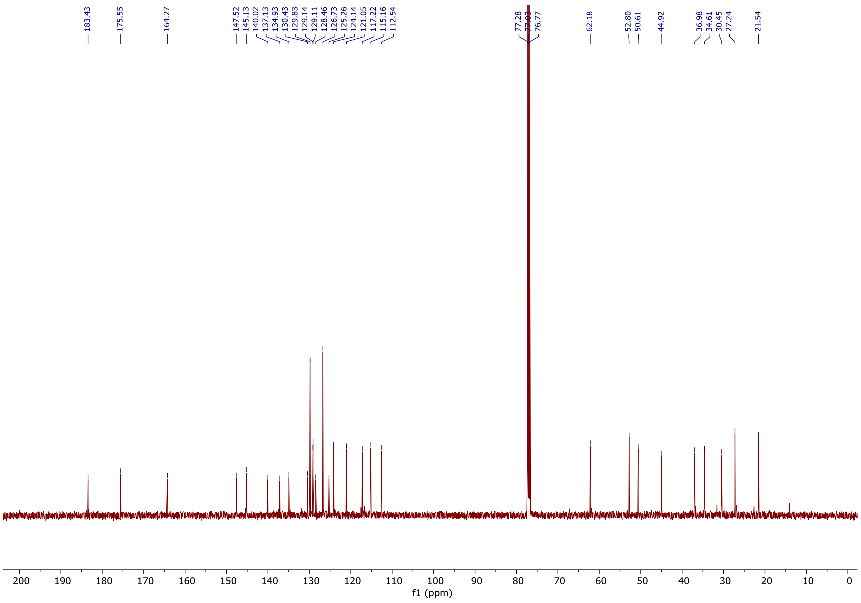

^{13}C NMR (125 MHz, $CDCl_3$) of (−)-**19**

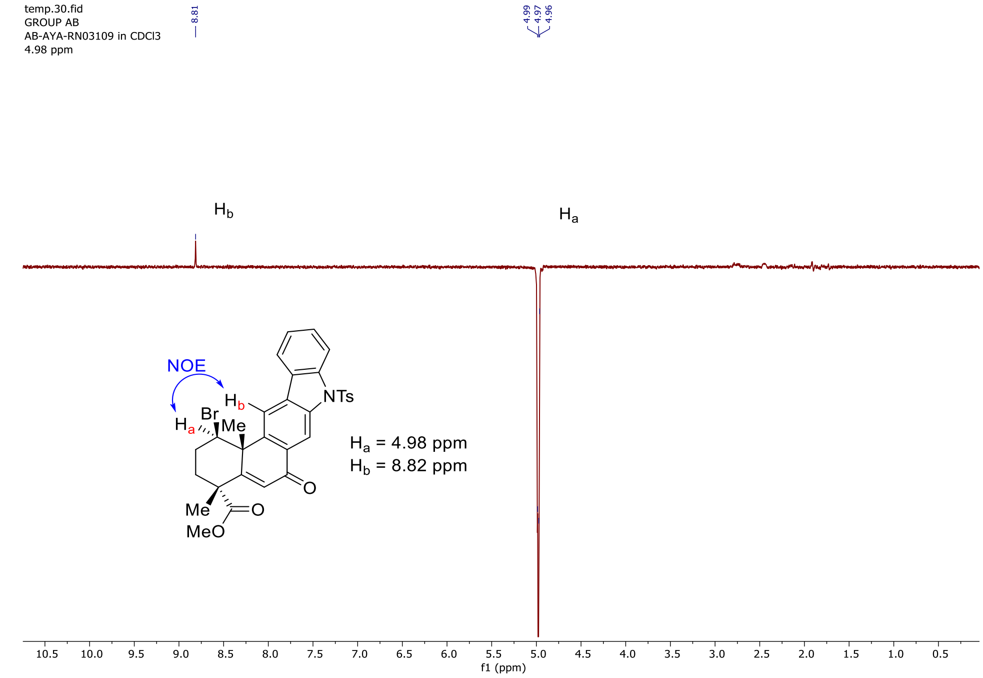

NOE (500 MHz, $CDCl_3$) of (–)-**19** considering 4.98 ppm

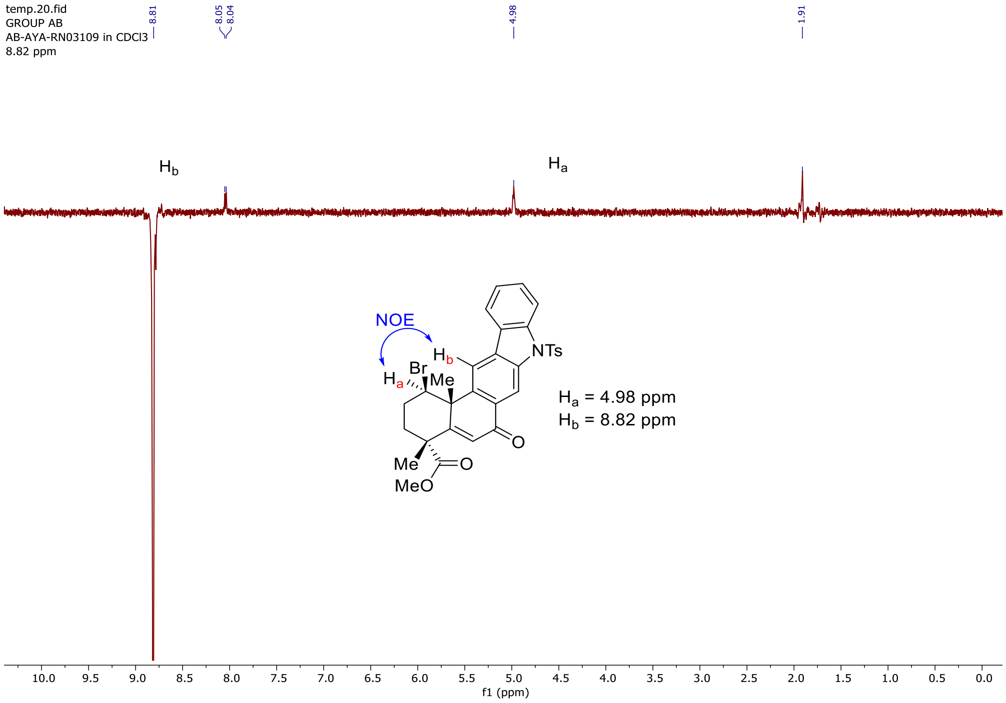

NOE (500 MHz, $CDCl_3$) of (–)-**19** considering 8.82 ppm

Display Report

Analysis Info

Analysis Name	D:\Data\User data\2022\MAR\ab_mm_01_305.d	Acquisition Date	3/10/2022 12:33:39 PM
Method	Tune_pos_Mid.m	Operator	IISER Kolkata
Sample Name	ab_mm_01_305	Instrument	maXis impact 8282001.00127
Comment	signal dropping issue		

Acquisition Parameter

Source Type	ESI	Ion Polarity	Positive	Set Nebulizer	0.4 Bar
Focus	Active	Set Capillary	3400 V	Set Dry Heater	200 °C
Scan Begin	50 m/z	Set End Plate Offset	-500 V	Set Dry Gas	4.0 l/min
Scan End	3000 m/z	Set Charging Voltage	2000 V	Set Divert Valve	Source
		Set Corona	0 nA	Set APCI Heater	0 °C

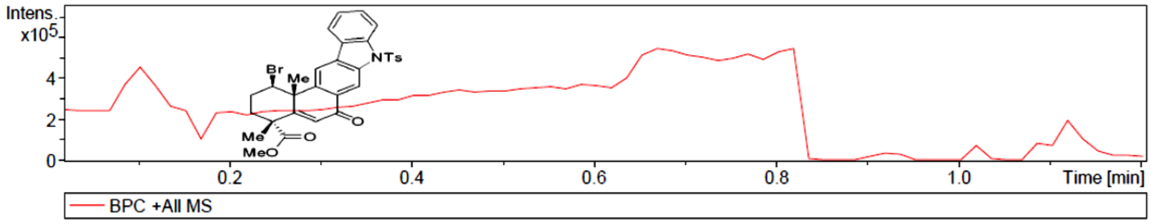

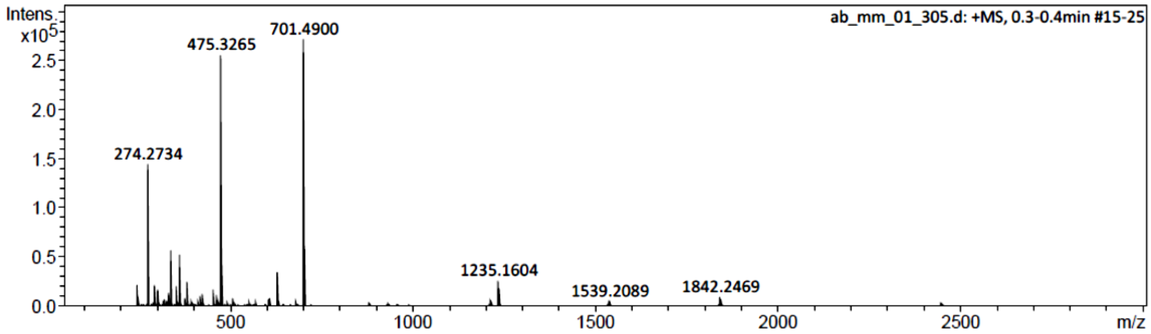

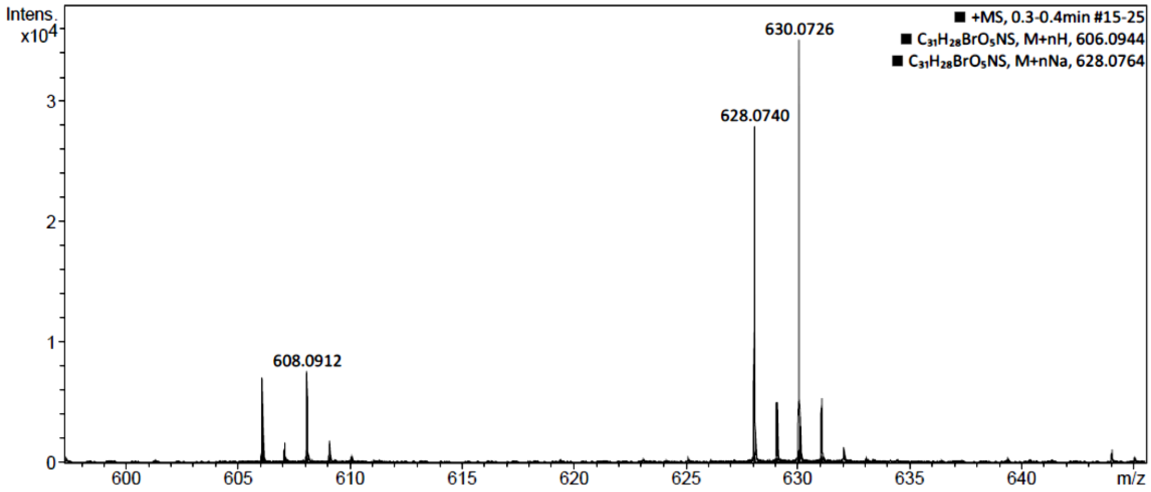

ab_mm_01_305.d

Bruker Compass DataAnalysis 4.1 printed: 3/10/2022 12:37:59 PM by: IISER Kolkata Page 1 of 1

HRMS data of (–)-**19**

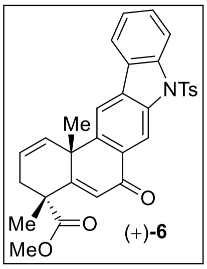

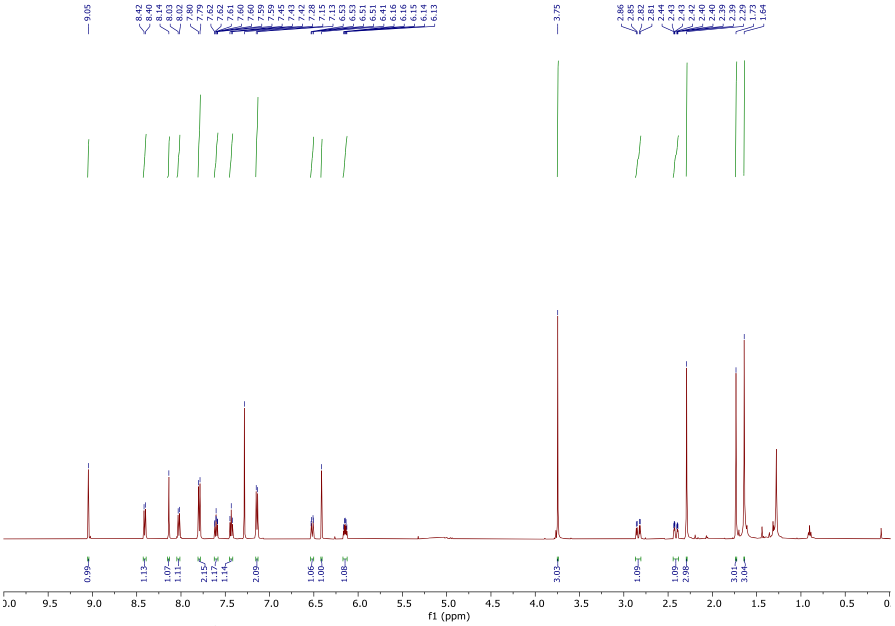

^{1}H NMR (400 MHz, $CDCl_3$) of (+)-**6**

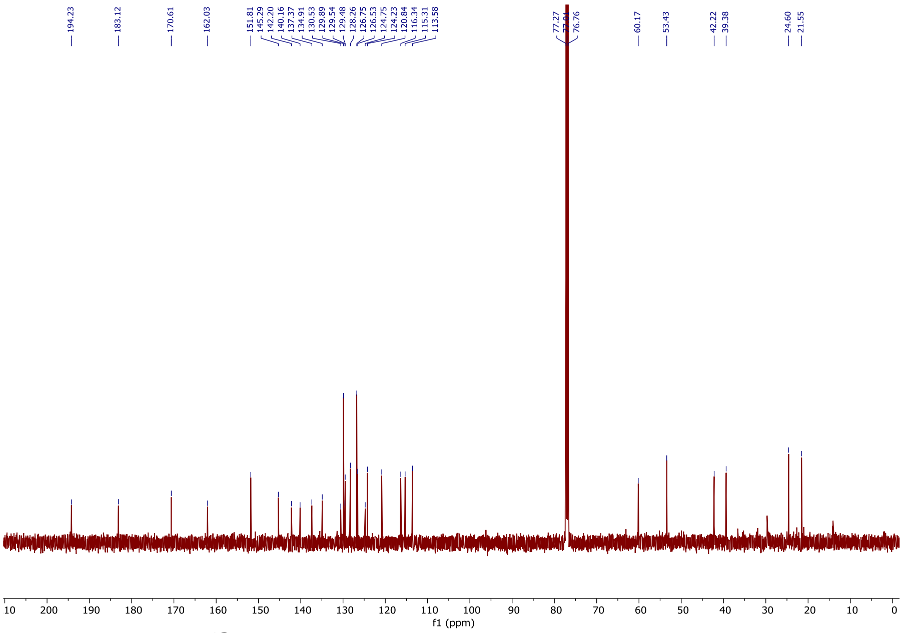

^{13}C NMR (125 MHz, $CDCl_3$) of (+)-**6**

Display Report

Analysis Info

Analysis Name	D:\Data\User data\2022\FEB\AB_MM_01_309.d	Acquisition Date	2/4/2022 11:55:44 AM
Method	Tune_pos_Mid.m	Operator	IISER Kolkata
Sample Name	AB_MM_01_309	Instrument	maXis impact 8282001.00127
Comment			

Acquisition Parameter

Source Type	ESI	Ion Polarity	Positive	Set Nebulizer	0.4 Bar
Focus	Active	Set Capillary	3400 V	Set Dry Heater	200 °C
Scan Begin	50 m/z	Set End Plate Offset	-500 V	Set Dry Gas	4.0 l/min
Scan End	3000 m/z	Set Charging Voltage	2000 V	Set Divert Valve	Source
		Set Corona	0 nA	Set APCI Heater	0 °C

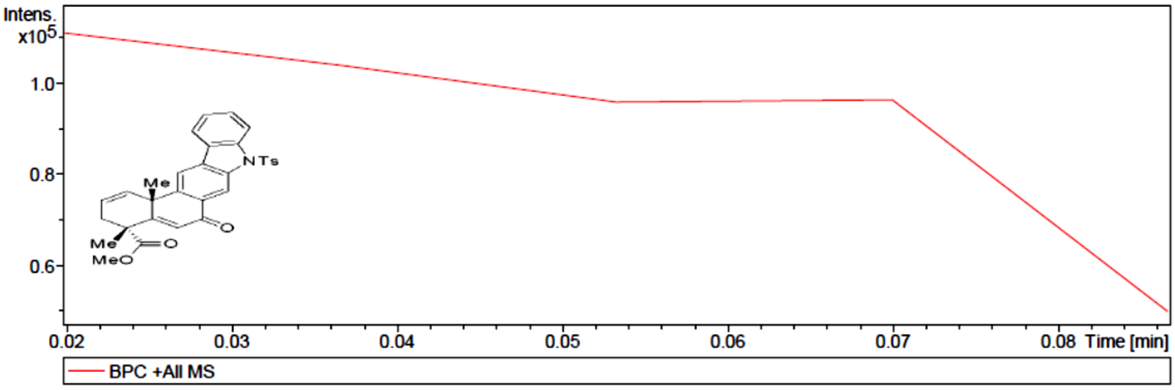

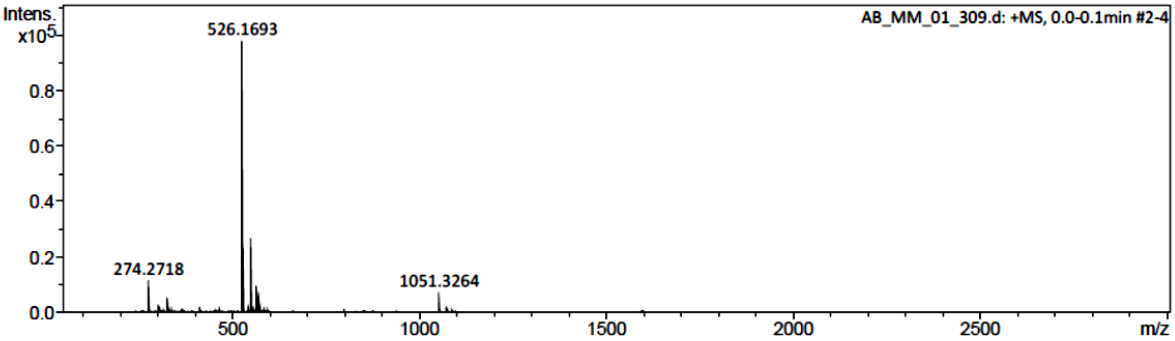

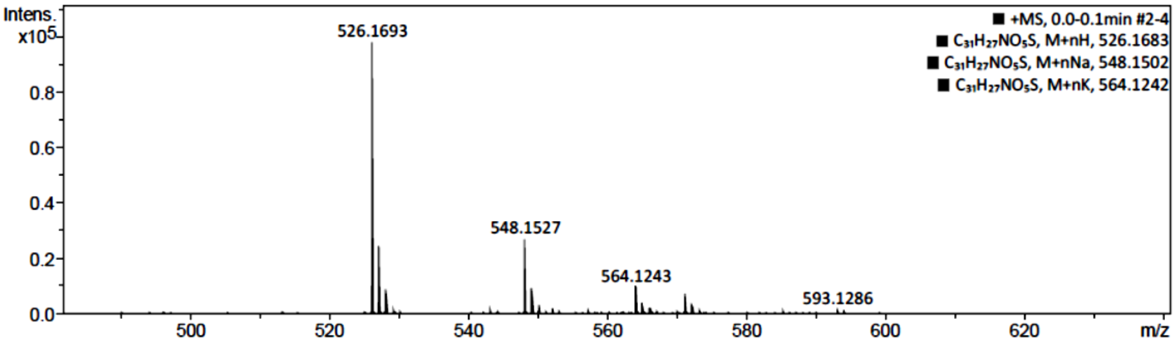

AB_MM_01_309.d
Bruker Compass DataAnalysis 4.1 printed: 2/4/2022 11:58:24 AM by: IISER Kolkata Page 1 of 1

HRMS data of (+)-**6**

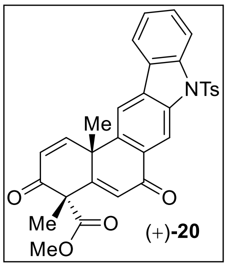

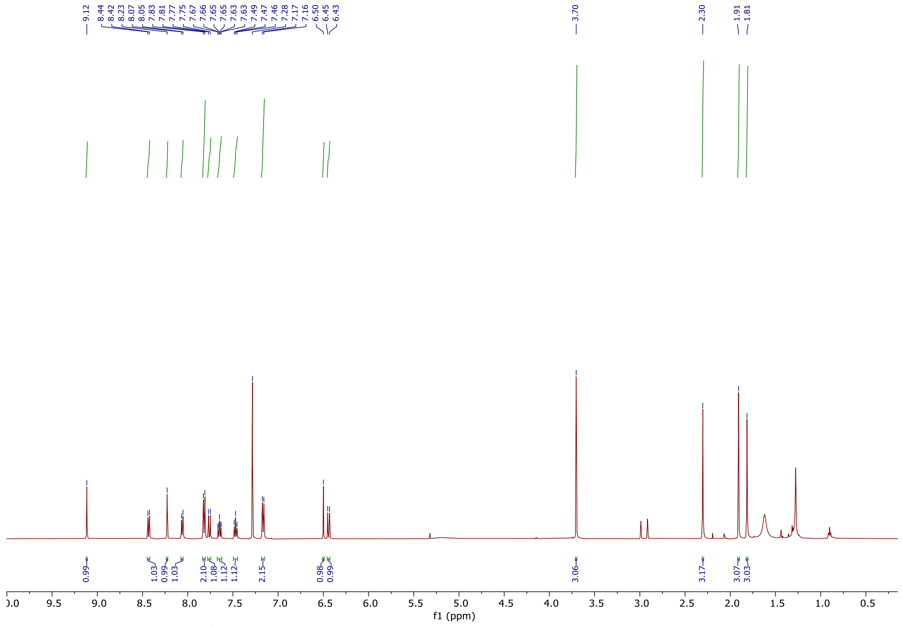

^{1}H NMR (500 MHz, $CDCl_3$) of (+)-**20**

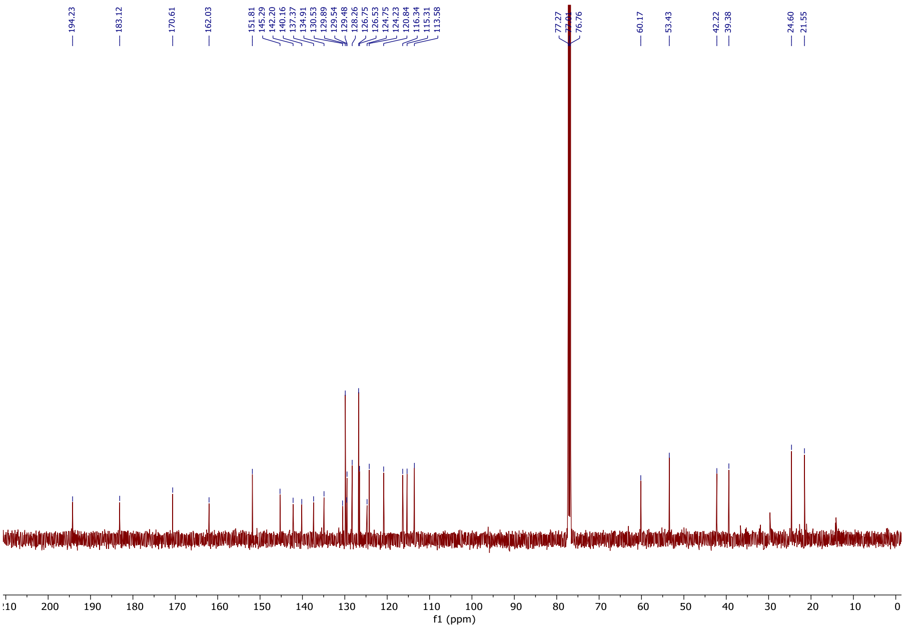

^{13}C NMR (125 MHz, $CDCl_3$) of (+)-**20**

Display Report

Analysis Info

Analysis Name	D:\Data\User data\2022\JAN\AB_SK_370.d	Acquisition Date	1/3/2022 1:18:24 PM
Method	Tune_pos_Standard.m	Operator	IISER Kolkata
Sample Name	AB_SK_370	Instrument	maXis impact 8282001.00127
Comment			

Acquisition Parameter

Source Type	ESI	Ion Polarity	Positive	Set Nebulizer	0.5 Bar
Focus	Active	Set Capillary	3400 V	Set Dry Heater	200 °C
Scan Begin	50 m/z	Set End Plate Offset	-500 V	Set Dry Gas	4.0 l/min
Scan End	1000 m/z	Set Charging Voltage	2000 V	Set Divert Valve	Source
		Set Corona	0 nA	Set APCI Heater	0 °C

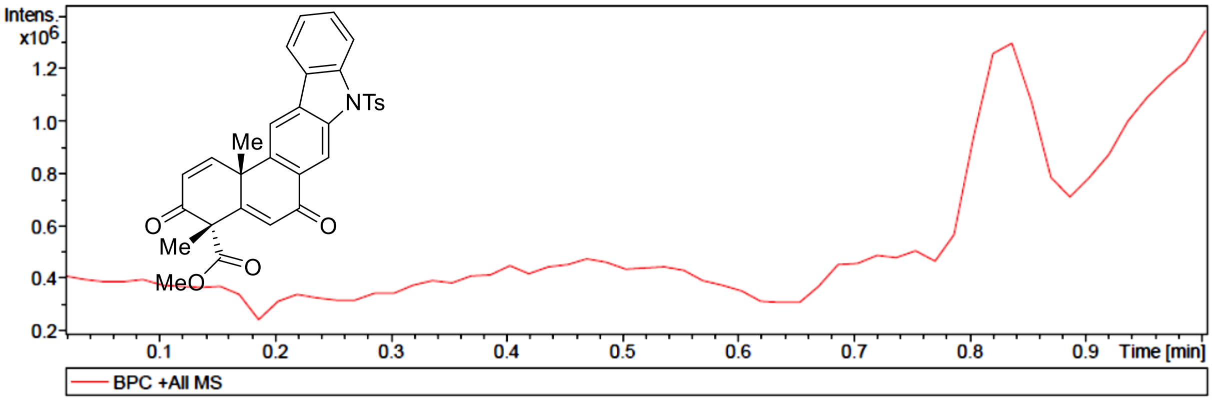

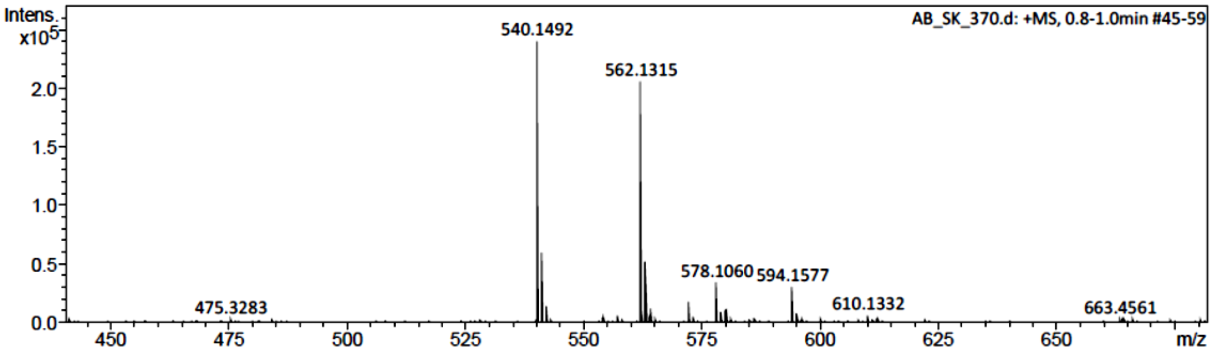

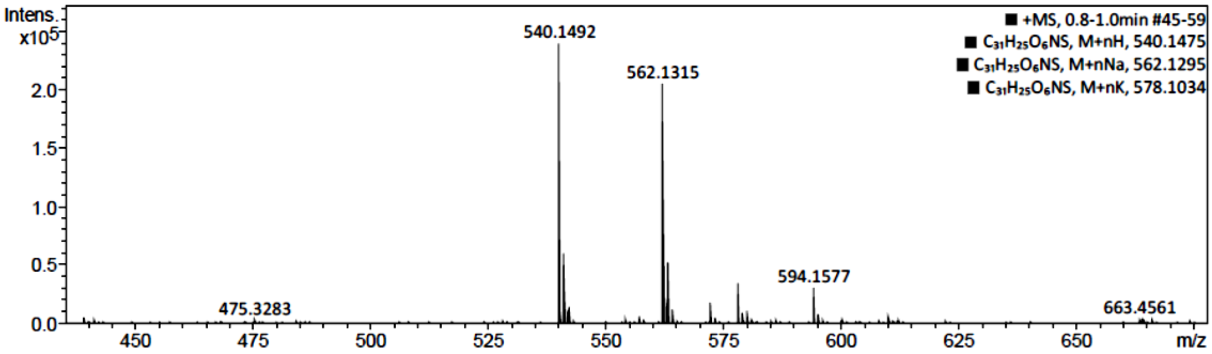

AB_SK_370.d

Bruker Compass DataAnalysis 4.1 printed: 1/3/2022 1:21:35 PM by: IISER Kolkata Page 1 of 1

HRMS data of (+)-**20**

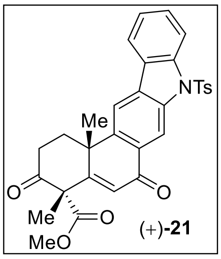

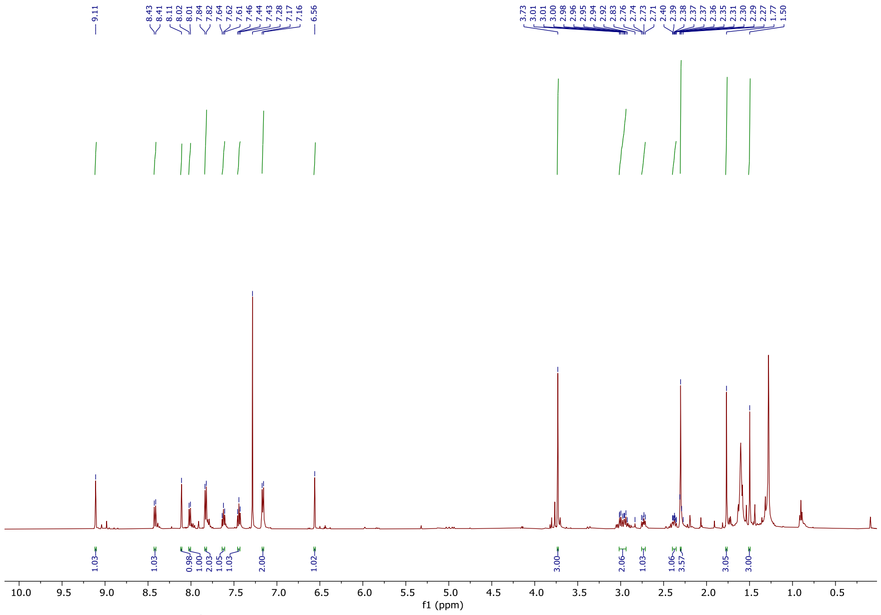

^{1}H NMR (500 MHz, $CDCl_3$) of (+)-**21**

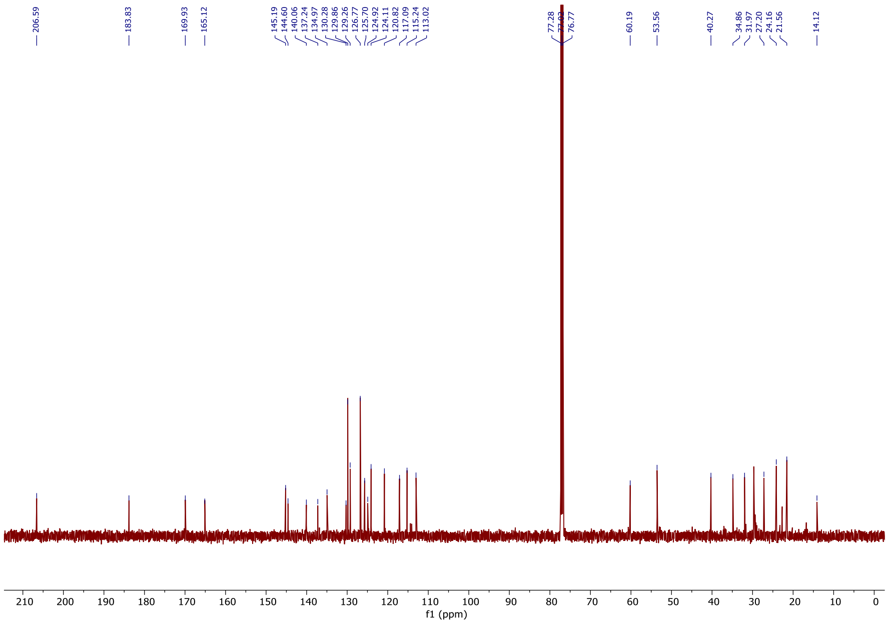

^{13}C NMR (125 MHz, $CDCl_3$) of (+)-**21**

Display Report

Analysis Info

Analysis Name	D:\Data\User data\2021\DEC\AB_SK_375.d	Acquisition Date	12/24/2021 2:38:37 PM
Method	Tune_pos_Mid.m	Operator	IISER Kolkata
Sample Name	AB_SK_375	Instrument	maXis impact 8282001.00127
Comment			

Acquisition Parameter

Source Type	ESI	Ion Polarity	Positive	Set Nebulizer	0.4 Bar
Focus	Active	Set Capillary	3400 V	Set Dry Heater	200 °C
Scan Begin	50 m/z	Set End Plate Offset	-500 V	Set Dry Gas	4.0 l/min
Scan End	3000 m/z	Set Charging Voltage	2000 V	Set Divert Valve	Source
		Set Corona	0 nA	Set APCI Heater	0 °C

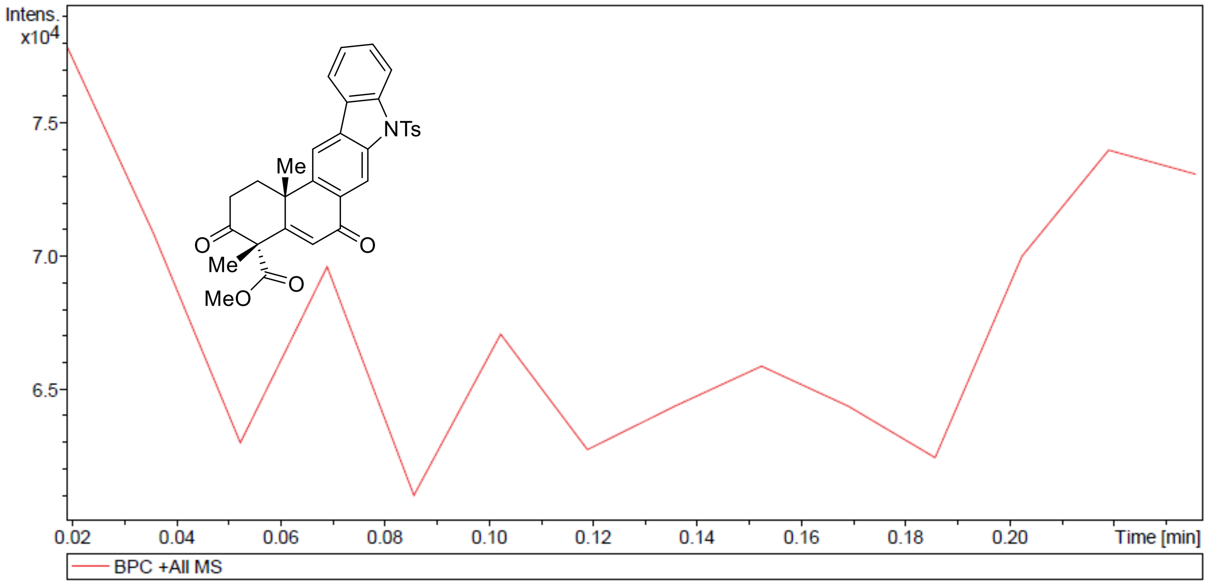

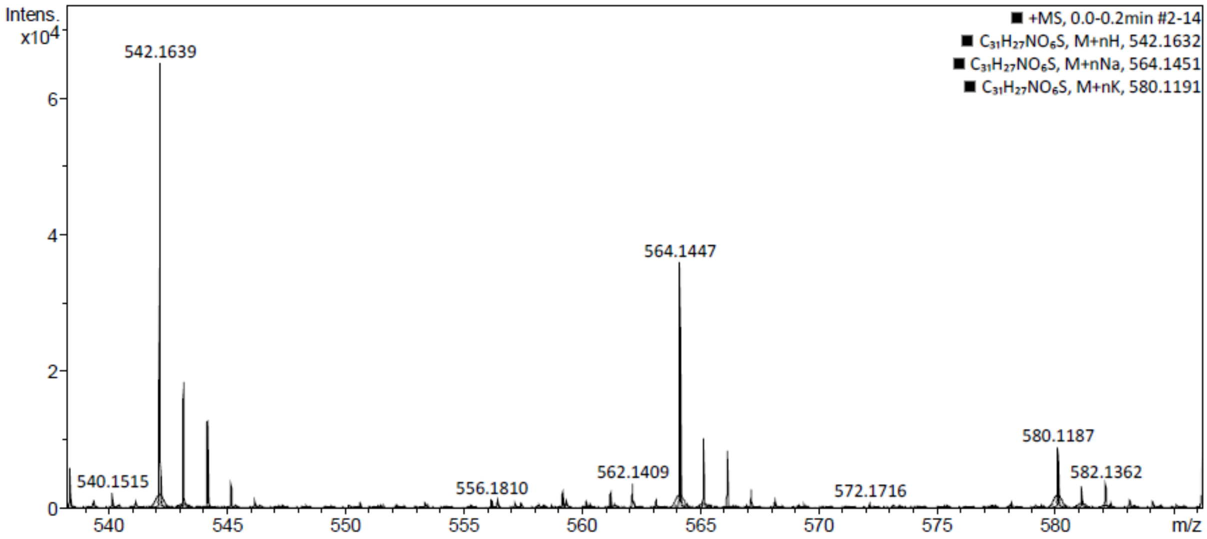

AB_SK_375.d

Bruker Compass DataAnalysis 4.1 printed: 12/24/2021 2:41:23 PM by: IISER Kolkata Page 1 of 1

HRMS data of (+)-**21**

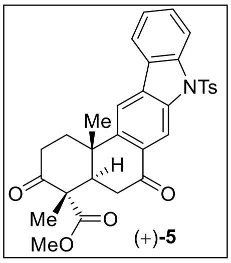

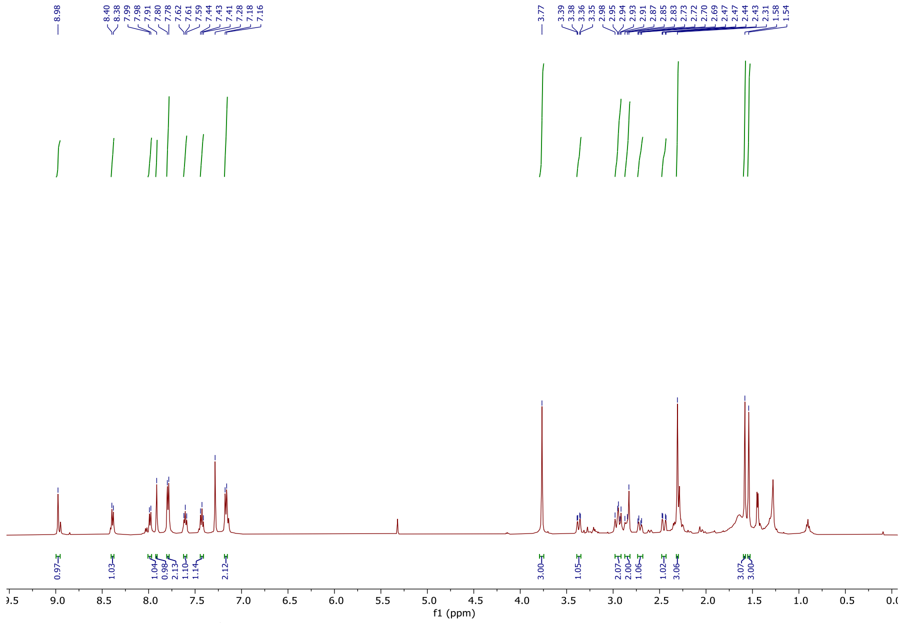

^{1}H NMR (500 MHz, $CDCl_3$) of (+)-**5**

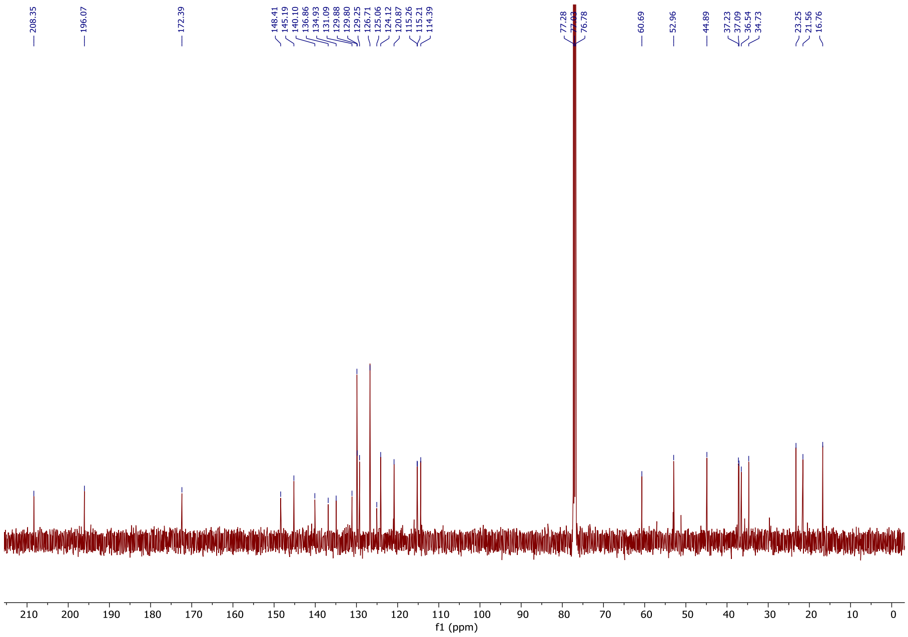

^{13}C NMR (125 MHz, $CDCl_3$) of (+)-**5**

Display Report

Analysis Info

Analysis Name	D:\Data\User data\2022\MAR\ab_sk_02154.d	Acquisition Date	3/25/2022 1:01:48 PM
Method	Tune_pos_Standard.m	Operator	IISER Kolkata
Sample Name	ab_sk_02154	Instrument	maXis impact 8282001.00127
Comment			

Acquisition Parameter

Source Type	ESI	Ion Polarity	Positive	Set Nebulizer	0.5 Bar
Focus	Active	Set Capillary	3400 V	Set Dry Heater	200 °C
Scan Begin	50 m/z	Set End Plate Offset	-500 V	Set Dry Gas	4.0 l/min
Scan End	1000 m/z	Set Charging Voltage	2000 V	Set Divert Valve	Source
		Set Corona	0 nA	Set APCI Heater	0 °C

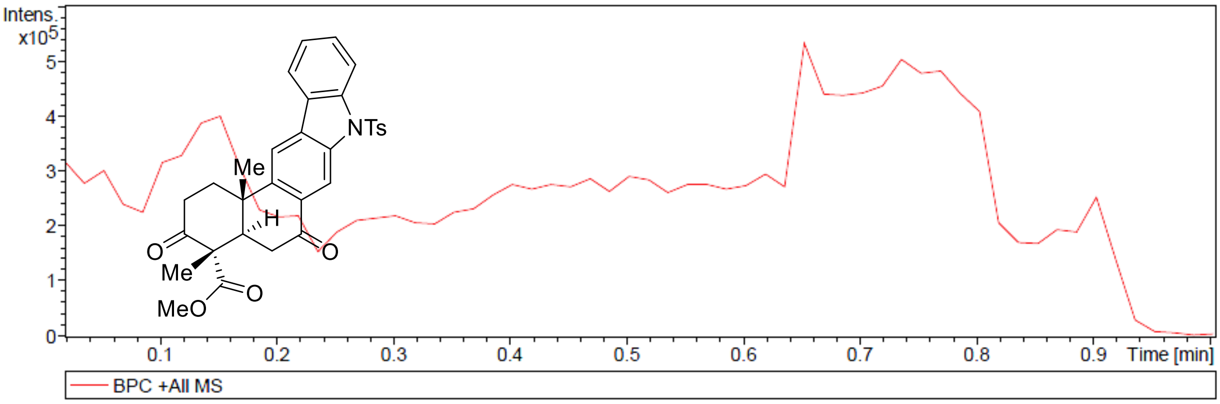

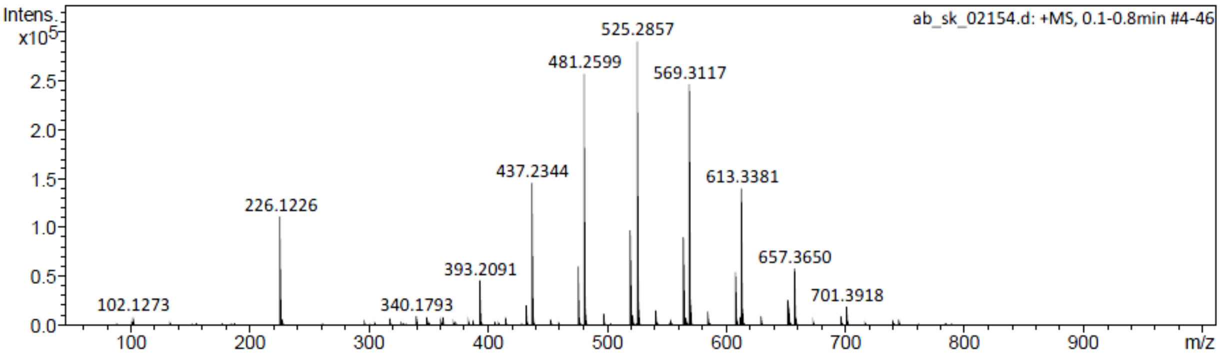

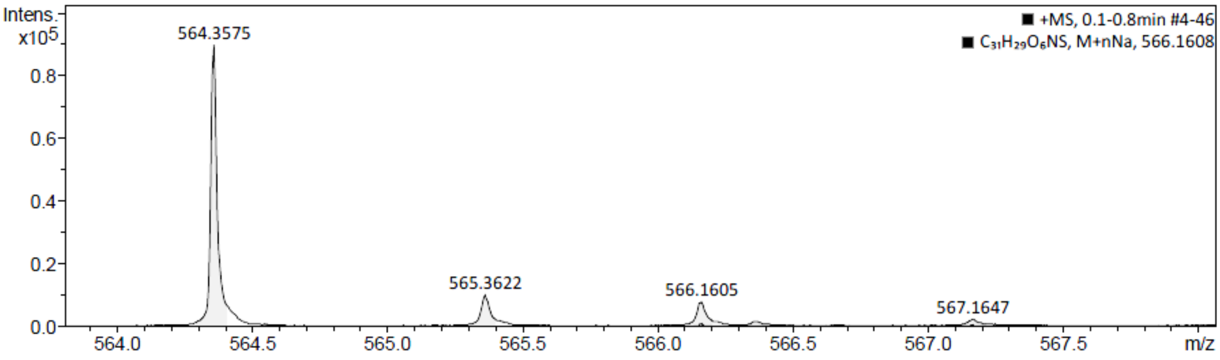

ab_sk_02154.d

Bruker Compass DataAnalysis 4.1 printed: 3/30/2022 1:01:35 PM by: IISER Kolkata Page 1 of 1

HRMS data of (+)-**5**

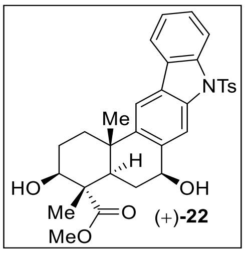

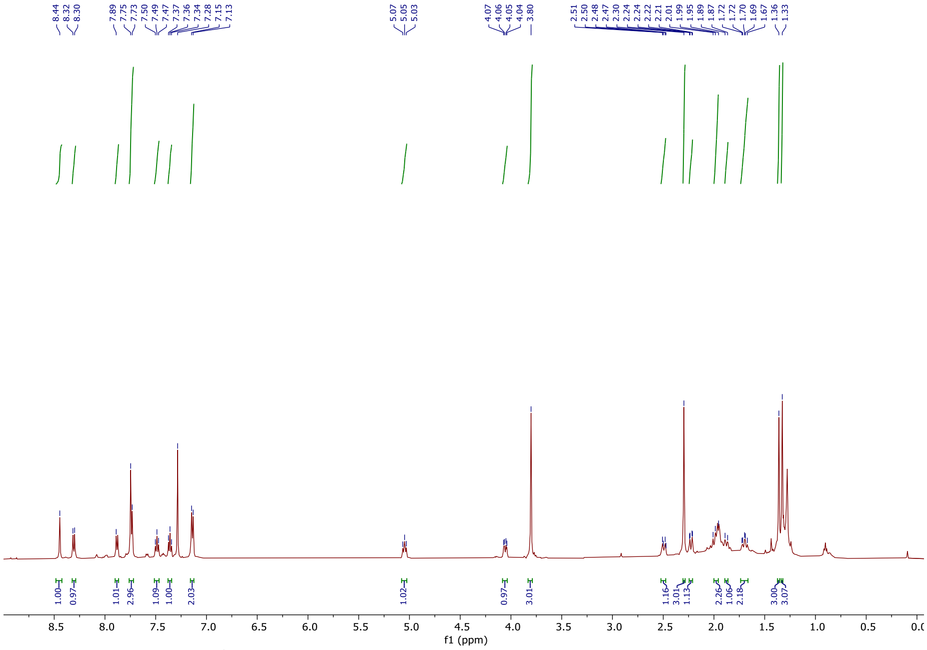

^{1}H NMR (500 MHz, $CDCl_3$) of (+)-**22**

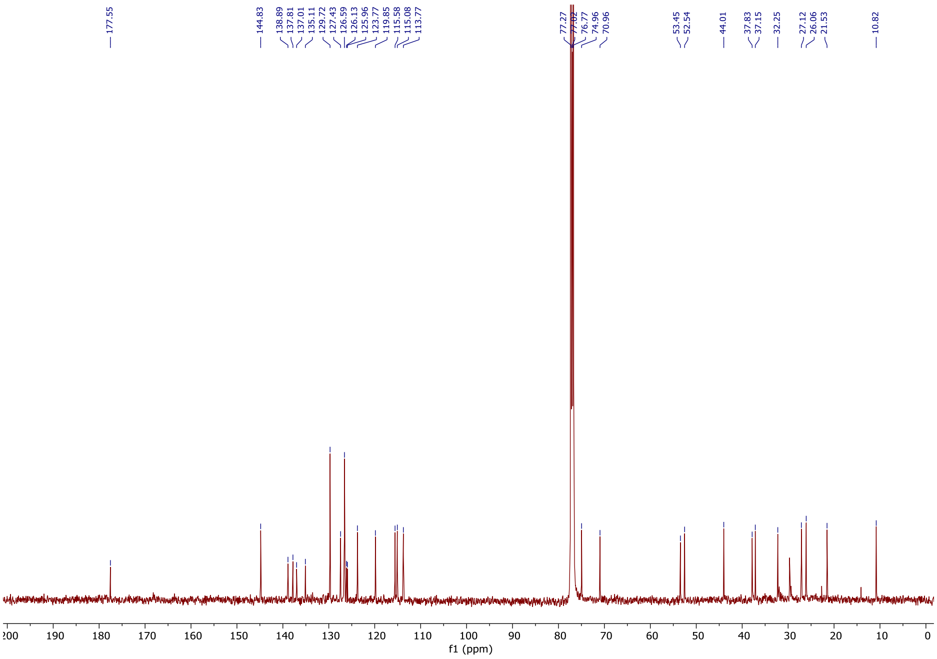

^{13}C NMR (125 MHz, $CDCl_3$) of (+)-**22**

Display Report

Analysis Info

Analysis Name	D:\Data\User data\2022\MAR\AB_SK_0255.d	Acquisition Date	3/31/2022 1:09:12 PM
Method	Tune_pos_Standard.m	Operator	IISER Kolkata
Sample Name	AB_SK_0255	Instrument	maXis impact 8282001.00127
Comment			

Acquisition Parameter

Source Type	ESI	Ion Polarity	Positive	Set Nebulizer	0.5 Bar
Focus	Active	Set Capillary	3400 V	Set Dry Heater	200 °C
Scan Begin	50 m/z	Set End Plate Offset	-500 V	Set Dry Gas	4.0 l/min
Scan End	1000 m/z	Set Charging Voltage	2000 V	Set Divert Valve	Source
		Set Corona	0 nA	Set APCI Heater	0 °C

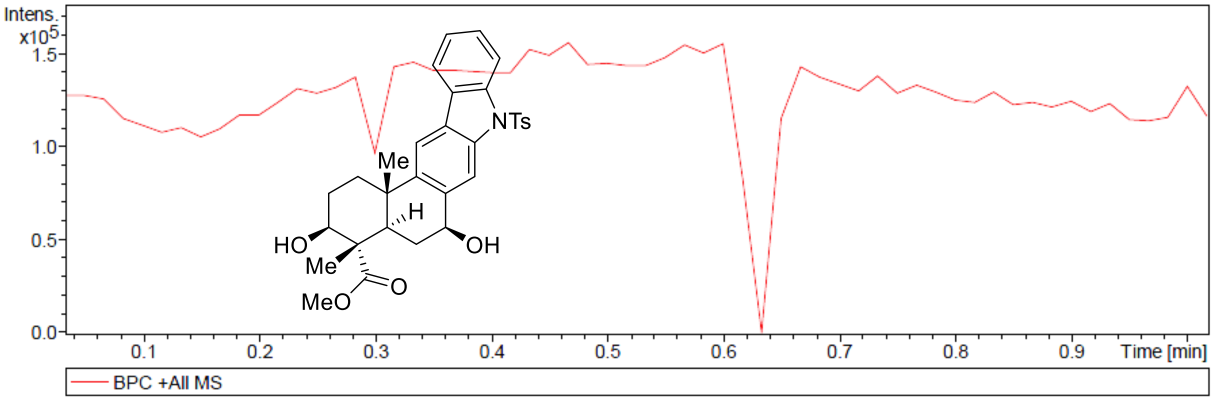

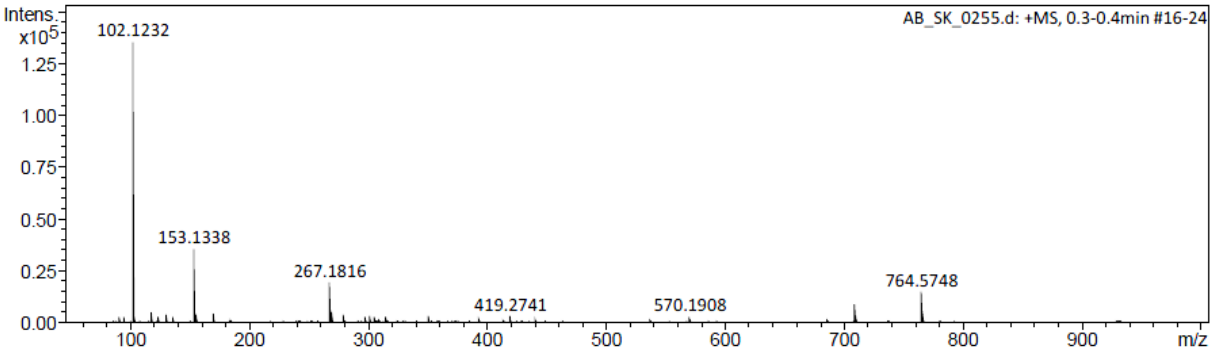

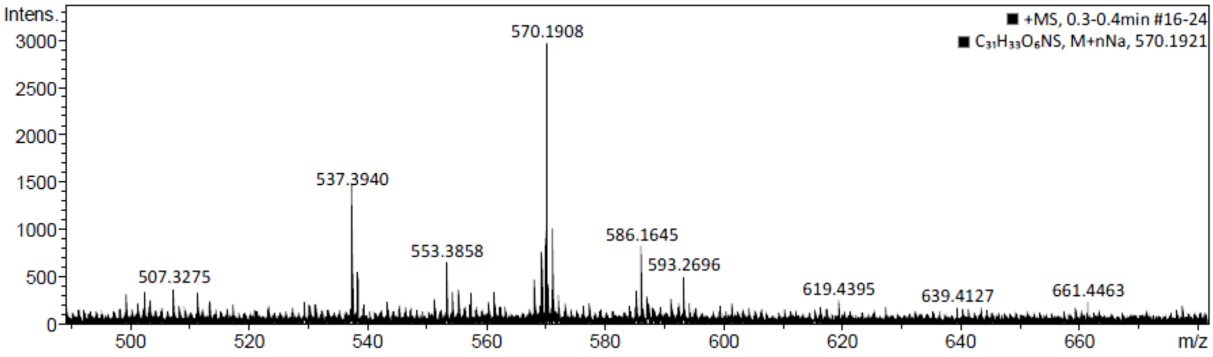

AB_SK_0255.d

Bruker Compass DataAnalysis 4.1 printed: 3/31/2022 1:11:28 PM by: IISER Kolkata Page 1 of 1

HRMS data of (+)-**22**

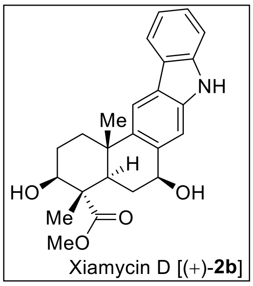

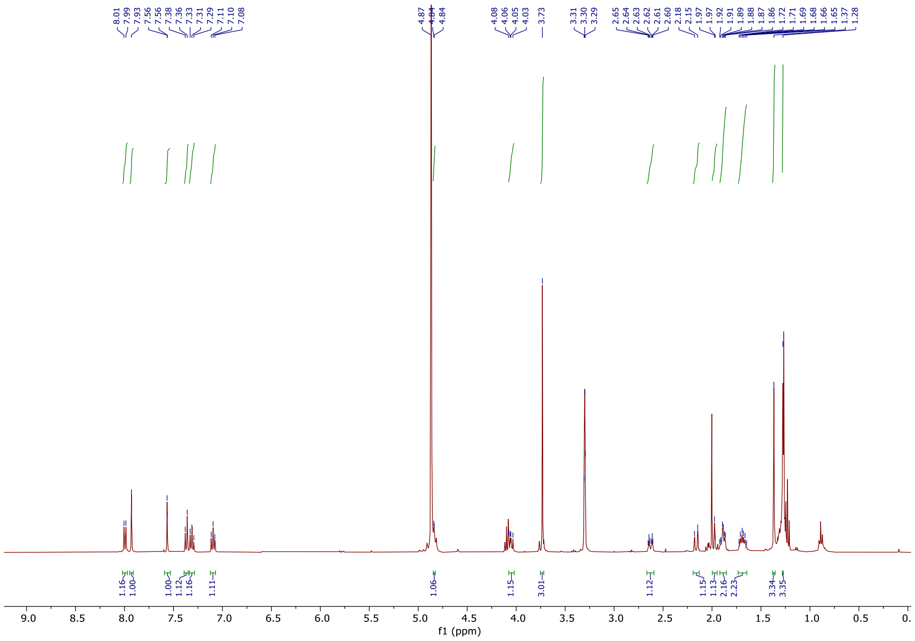

^{1}**H NMR** (400 MHz, CD_3OD) of Xiamycin D [(+)-**2b**]

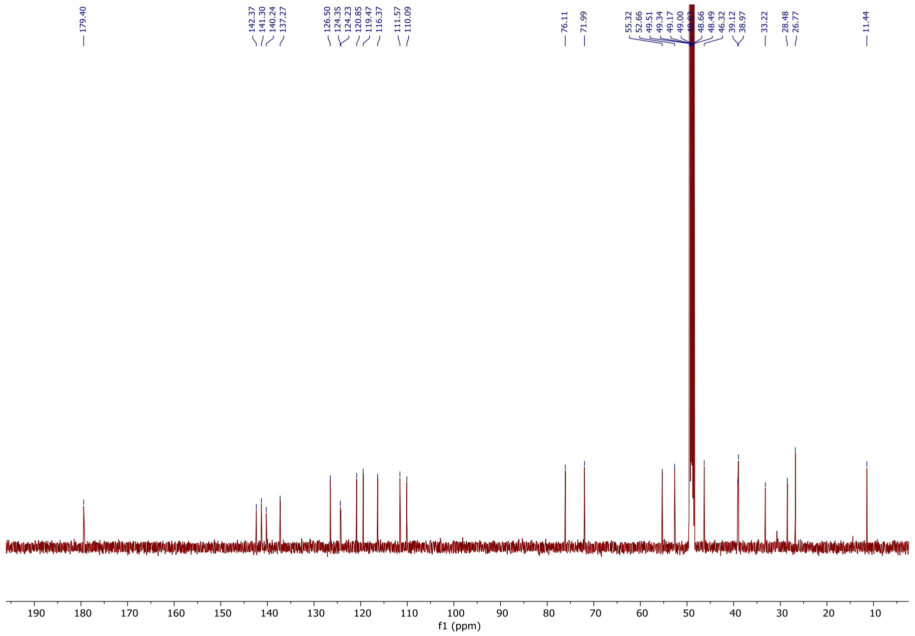

^{13}**C NMR** (125 MHz, CD_3OD) of Xiamycin D [(+)-**2b**]

Display Report

Analysis Info

Analysis Name	D:\Data\User data\2021\AUGUST\AB_RN_024.d	Acquisition Date	8/31/2021 11:42:36 AM
Method	Tune_pos_Standard.m	Operator	IISER Kolkata
Sample Name	AB_RN_024	Instrument	maXis impact 8282001.00127
Comment			

Acquisition Parameter

Source Type	ESI	Ion Polarity	Positive	Set Nebulizer	0.5 Bar
Focus	Active	Set Capillary	3400 V	Set Dry Heater	200 °C
Scan Begin	50 m/z	Set End Plate Offset	-500 V	Set Dry Gas	4.0 l/min
Scan End	1000 m/z	Set Charging Voltage	2000 V	Set Divert Valve	Source
		Set Corona	0 nA	Set APCI Heater	0 °C

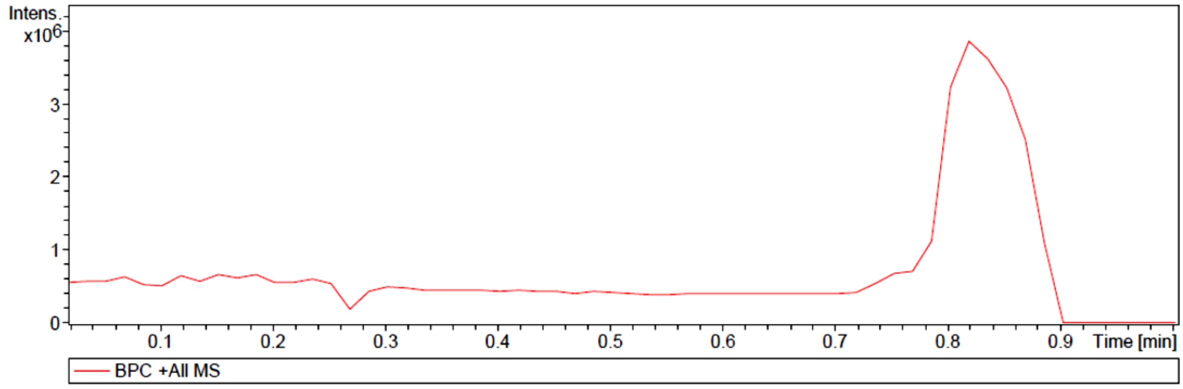

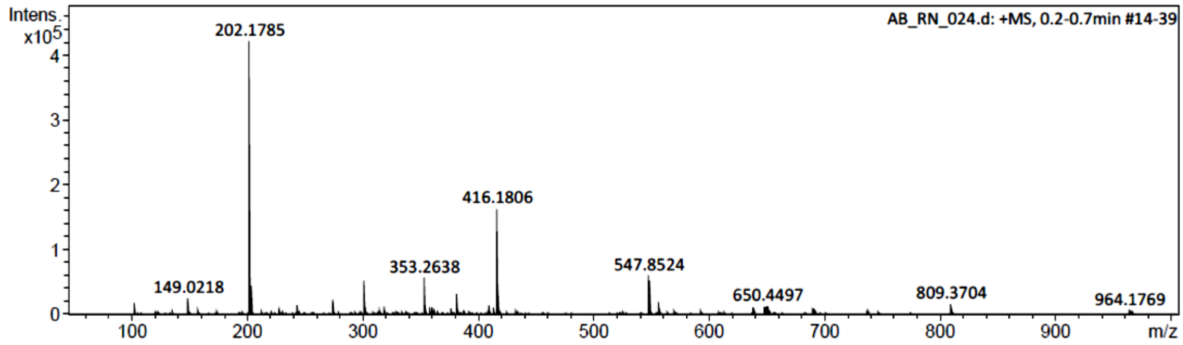

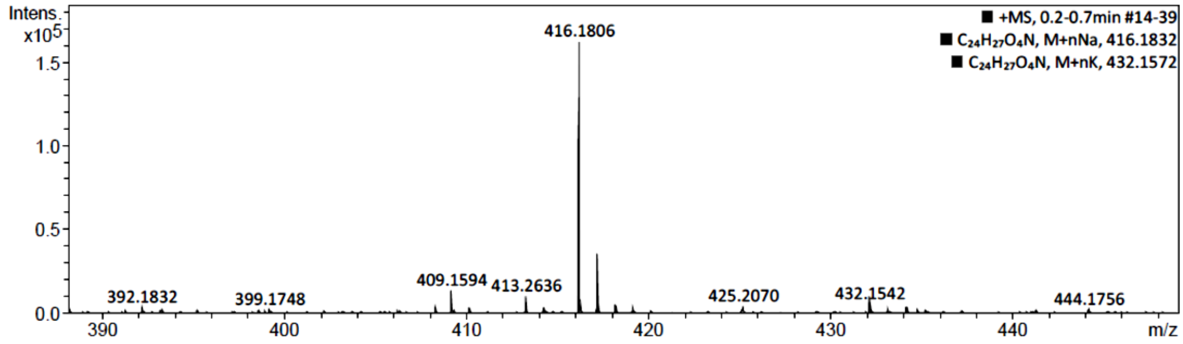

AB_RN_024.d
Bruker Compass DataAnalysis 4.1 printed: 8/31/2021 11:46:08 AM by: IISER Kolkata Page 1 of 1

HRMS data of Xiamycin D [(+)-**2b**]

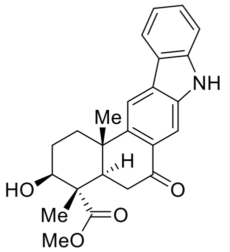

Xiamycin E [(+)-**2c**]

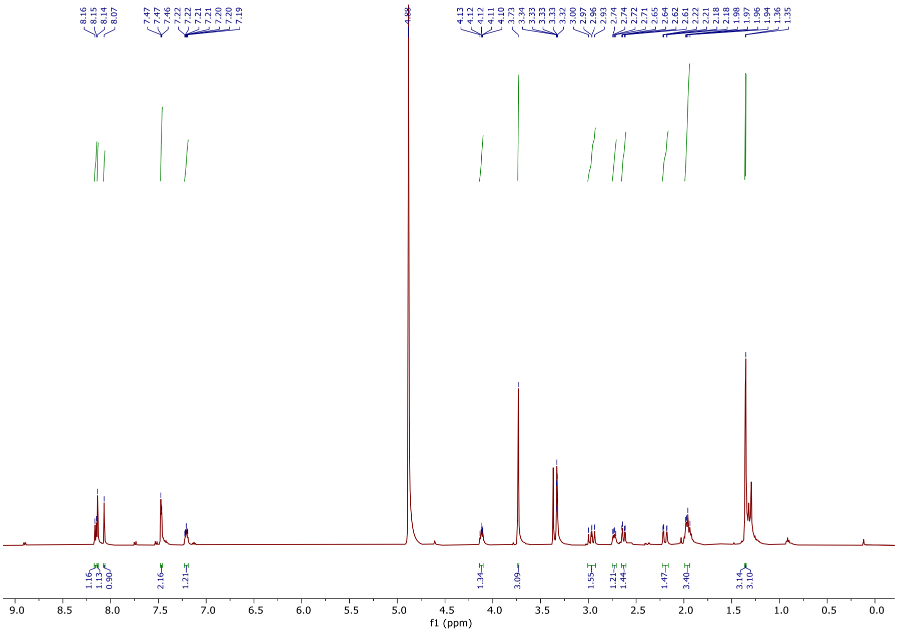

^{1}H NMR (500 MHz, CD_3OD) Xiamycin E [(+)-**2c**]

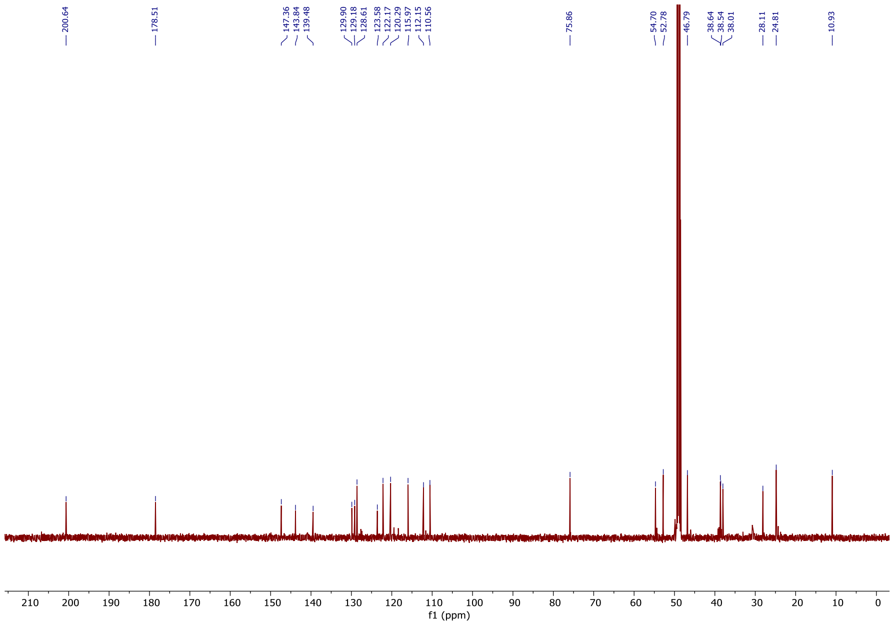

^{13}C NMR (125 MHz, CD_3OD) of Xiamycin E [(+)-**2c**]

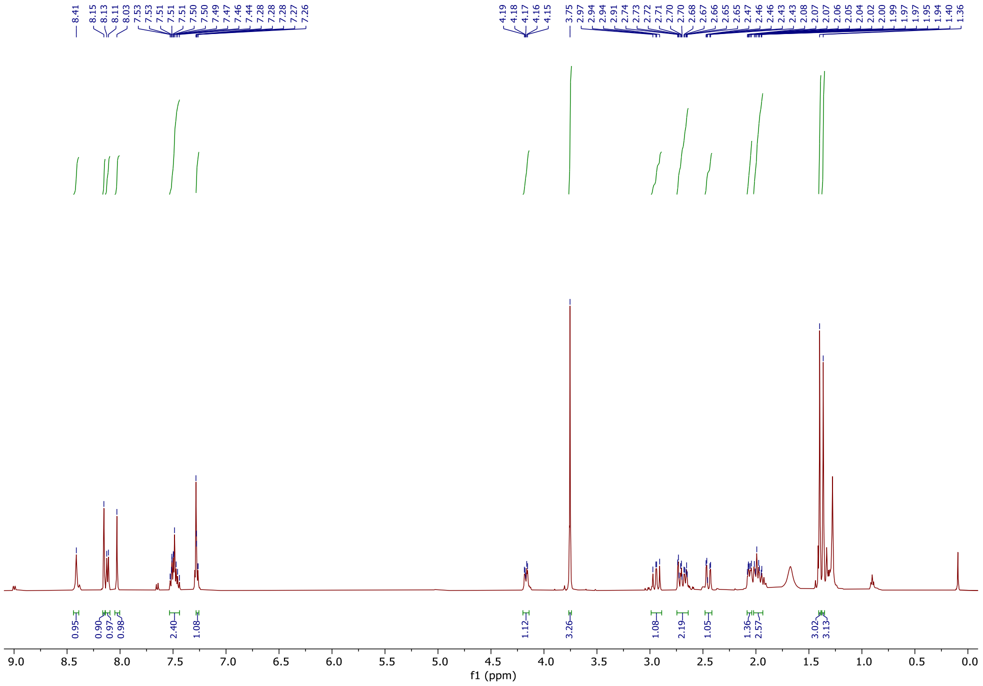

^{1}H NMR (500 MHz, $CDCl_3$) Xiamycin E [(+)-**2c**]

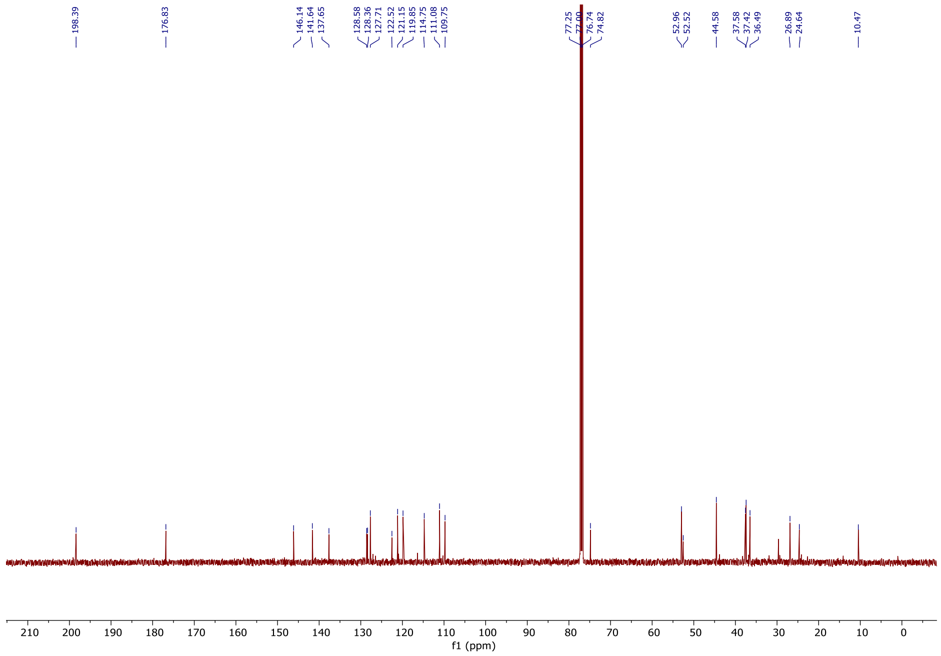

^{13}C NMR (125 MHz, $CDCl_3$) of Xiamycin E [(+)-**2c**]

Display Report

Analysis Info

Analysis Name	D:\Data\User data\2021\AUGUST\AB_RN_234.d	Acquisition Date	8/23/2021 10:31:08 AM
Method	Tune_pos_Standard.m	Operator	IISER Kolkata
Sample Name	AB_RN_234	Instrument	maXis impact 8282001.00127
Comment			

Acquisition Parameter

Source Type	ESI	Ion Polarity	Positive	Set Nebulizer	0.5 Bar
Focus	Active	Set Capillary	3400 V	Set Dry Heater	200 °C
Scan Begin	50 m/z	Set End Plate Offset	-500 V	Set Dry Gas	4.0 l/min
Scan End	1000 m/z	Set Charging Voltage	2000 V	Set Divert Valve	Source
		Set Corona	0 nA	Set APCI Heater	0 °C

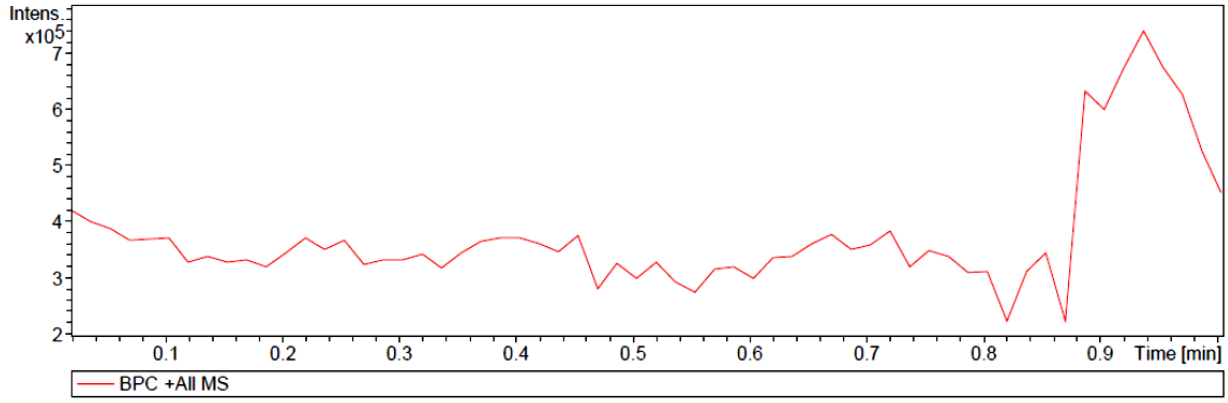

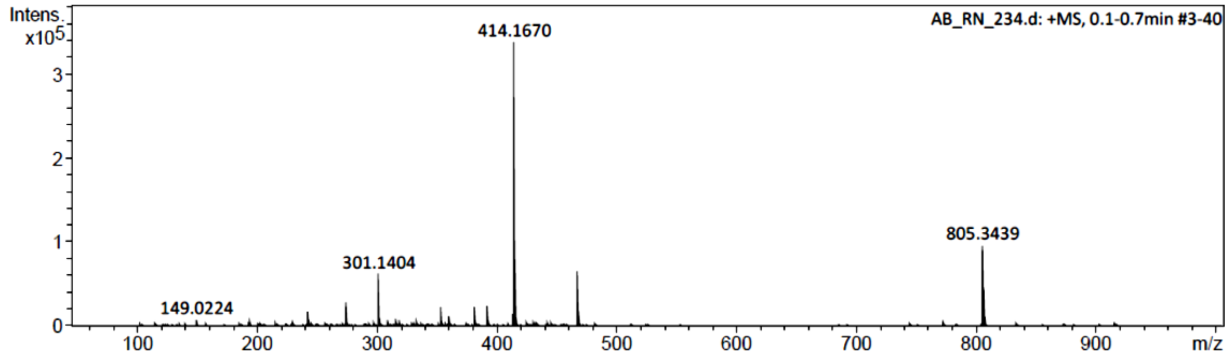

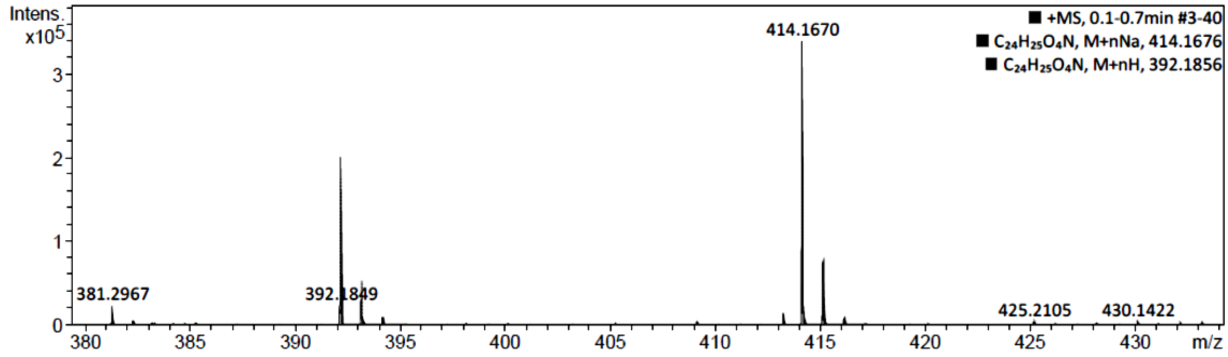

AB_RN_234.d

Bruker Compass DataAnalysis 4.1 printed: 8/23/2021 10:34:53 AM by: IISER Kolkata Page 1 of 1

HRMS data of Xiamycin E [(+)-**2c**]

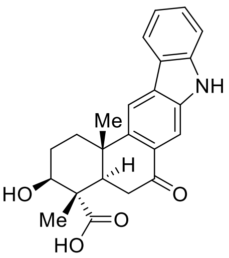

Xiamycin F [(+)-**2d**]

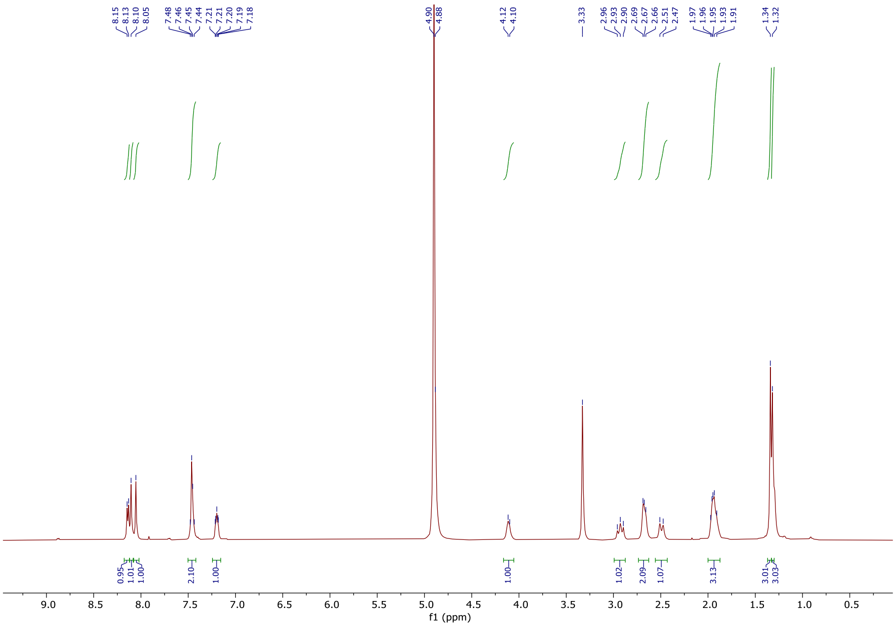

¹H NMR (500 MHz, CD_3OD) of Xiamycin F [(+)-**2d**]

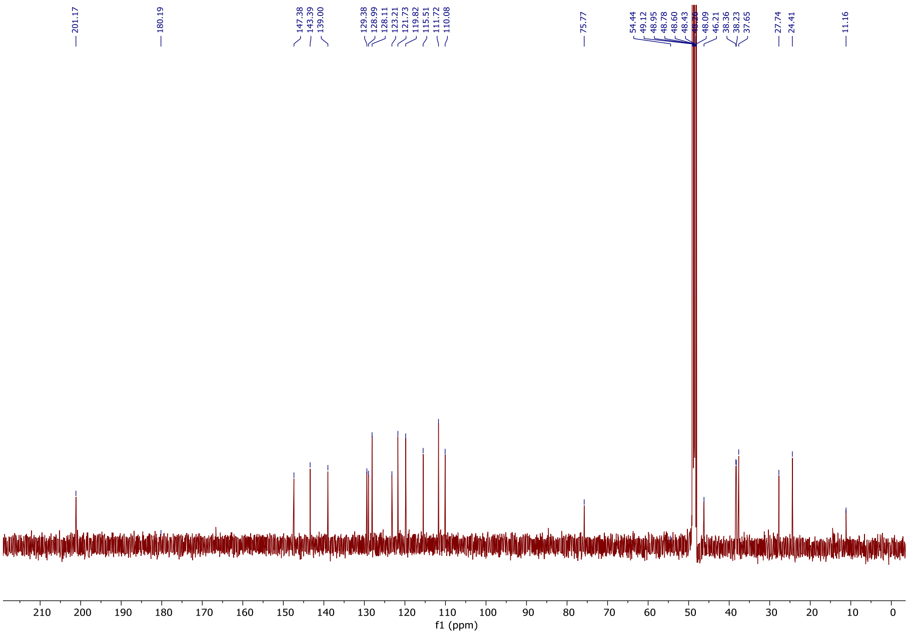

¹³C NMR (125 MHz, CD_3OD) of Xiamycin F [(+)-**2d**]

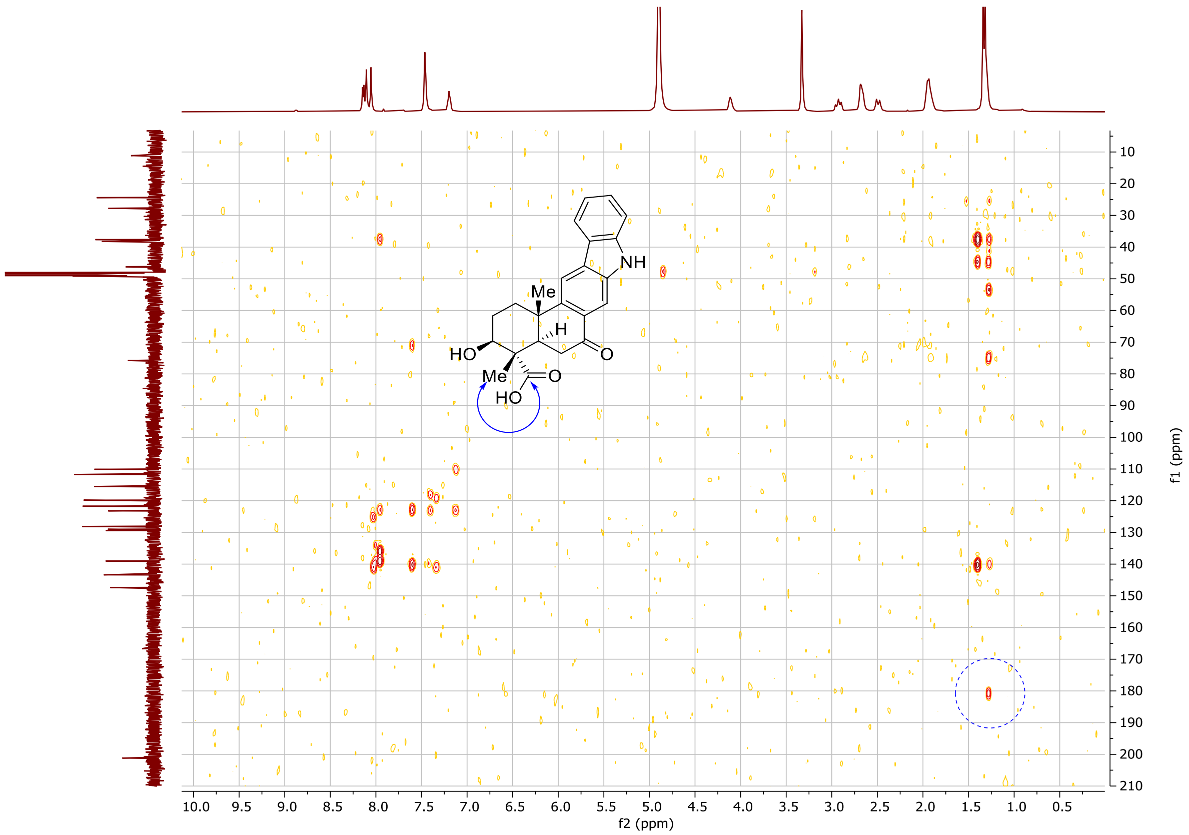

HMBC Data of Xiamycin F [(+)-**2d**]

Display Report

Analysis Info

Analysis Name	D:\Data\User data\2021\AUGUST\AB_RN_0238.d	Acquisition Date	8/26/2021 4:24:35 PM
Method	Tune_pos_Mid.m	Operator	IISER Kolkata
Sample Name	AB_RN_0238	Instrument	maXis impact 8282001.00127
Comment			

Acquisition Parameter

Source Type	ESI	Ion Polarity	Positive	Set Nebulizer	0.4 Bar
Focus	Active	Set Capillary	3400 V	Set Dry Heater	200 °C
Scan Begin	50 m/z	Set End Plate Offset	-500 V	Set Dry Gas	4.0 l/min
Scan End	3000 m/z	Set Charging Voltage	2000 V	Set Divert Valve	Source
		Set Corona	0 nA	Set APCI Heater	0 °C

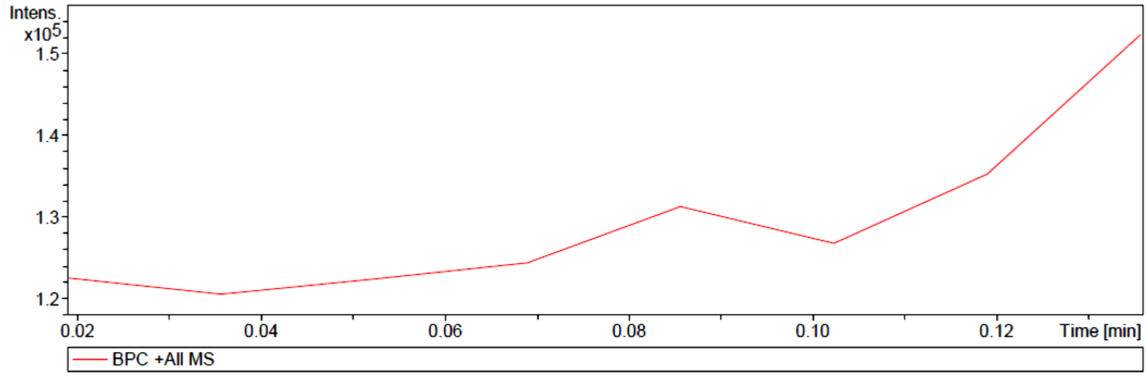

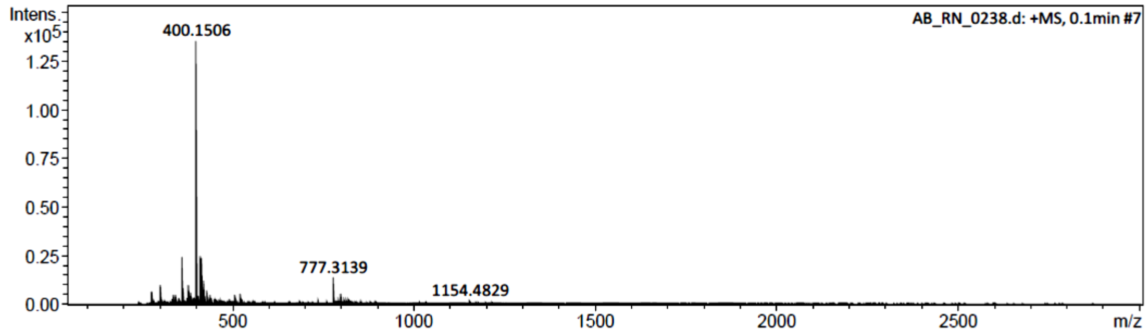

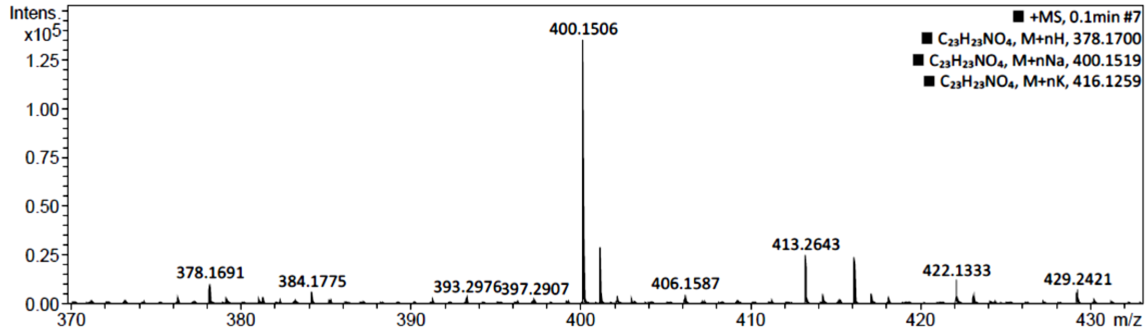

AB_RN_0238.d

Bruker Compass DataAnalysis 4.1 printed: 8/26/2021 4:27:24 PM by: IISER Kolkata Page 1 of 1

HRMS data of Xiamycin F [(+)-**2d**]

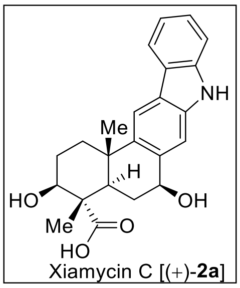

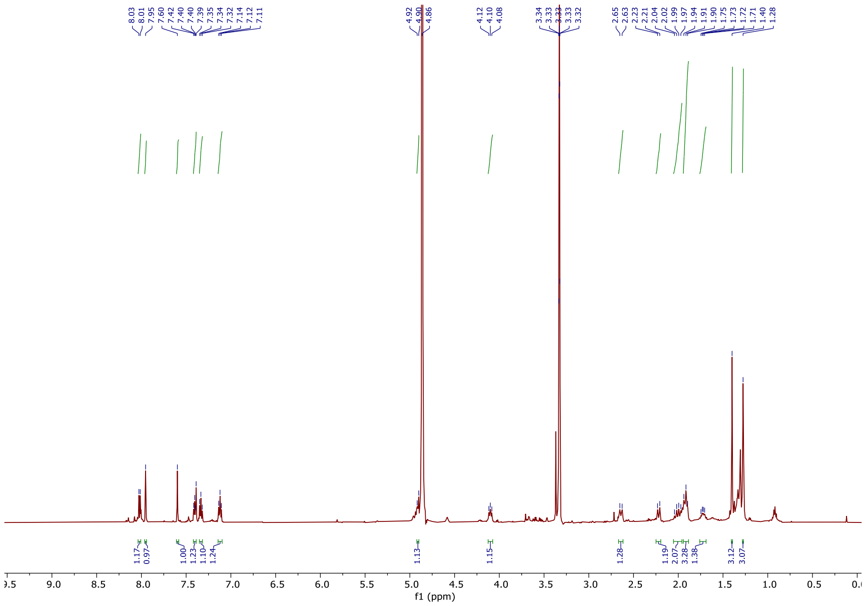

¹H NMR (500 MHz, CD3OD) of Xiamycin C [(+)-**2a**]

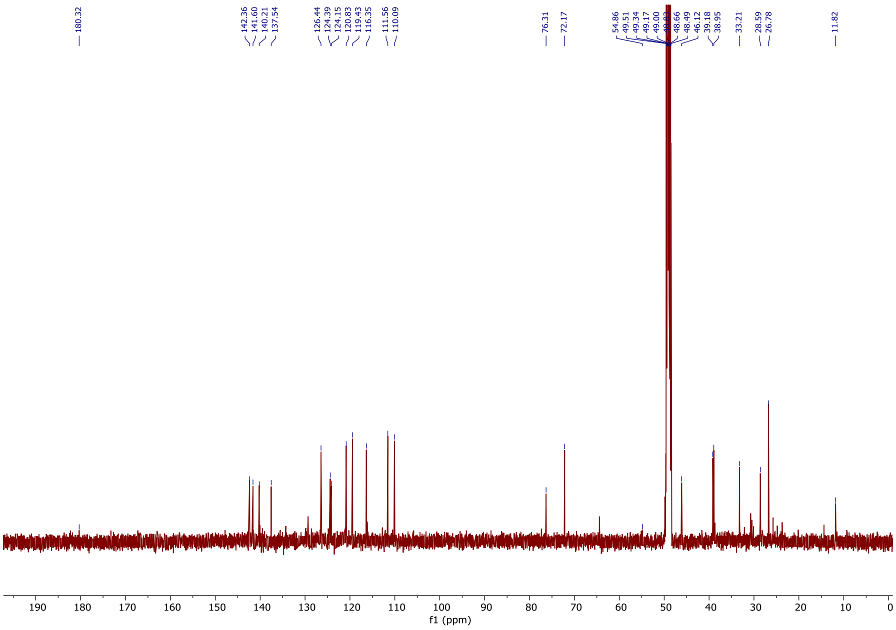

¹³C NMR (125 MHz, CD3OD) of Xiamycin C [(+)-**2a**]

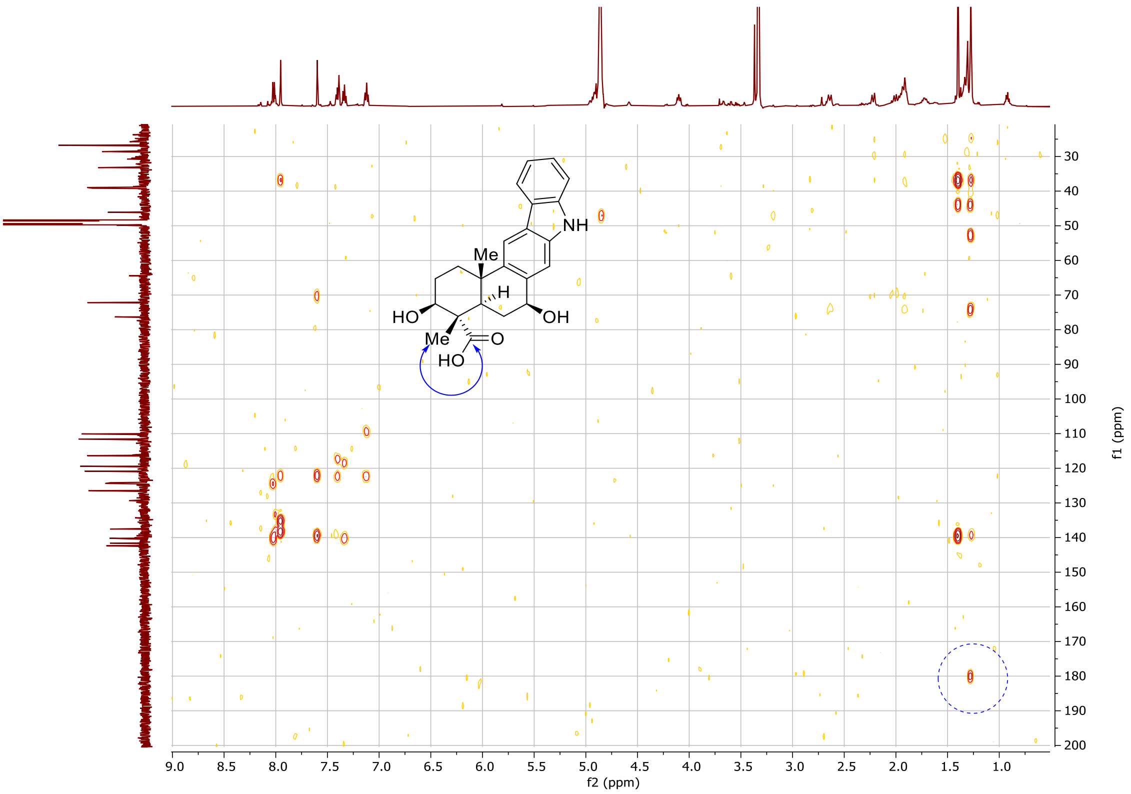

HMBC Data of Xiamycin C [(+)-**2a**]

Display Report

Analysis Info

Analysis Name	D:\Data\User data\2022\APRIL\ab_rn_03055.d	Acquisition Date	4/18/2022 12:37:48 PM
Method	Tune_pos_Standard.m	Operator	IISER Kolkata
Sample Name	ab_rn_03055	Instrument	maXis impact 8282001.00127
Comment			

Acquisition Parameter

Source Type	ESI	Ion Polarity	Positive	Set Nebulizer	0.5 Bar
Focus	Active	Set Capillary	3400 V	Set Dry Heater	200 °C
Scan Begin	50 m/z	Set End Plate Offset	-500 V	Set Dry Gas	4.0 l/min
Scan End	1000 m/z	Set Charging Voltage	2000 V	Set Divert Valve	Source
		Set Corona	0 nA	Set APCI Heater	0 °C

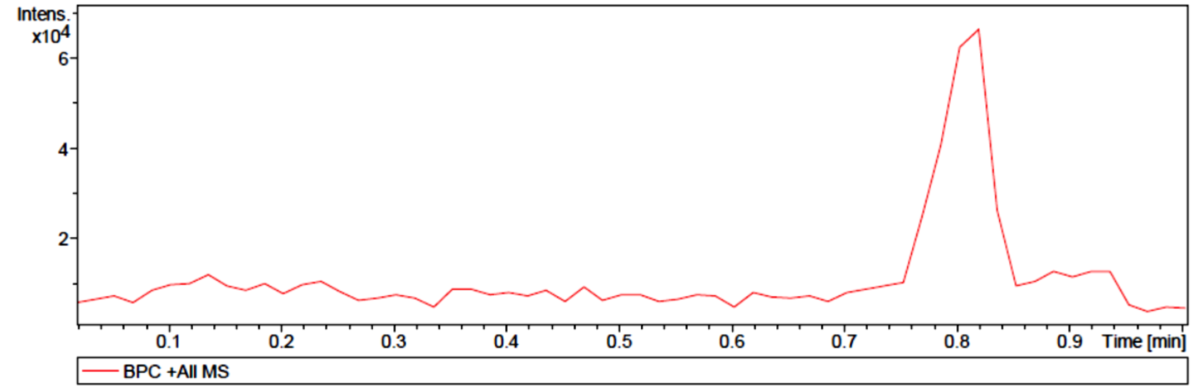

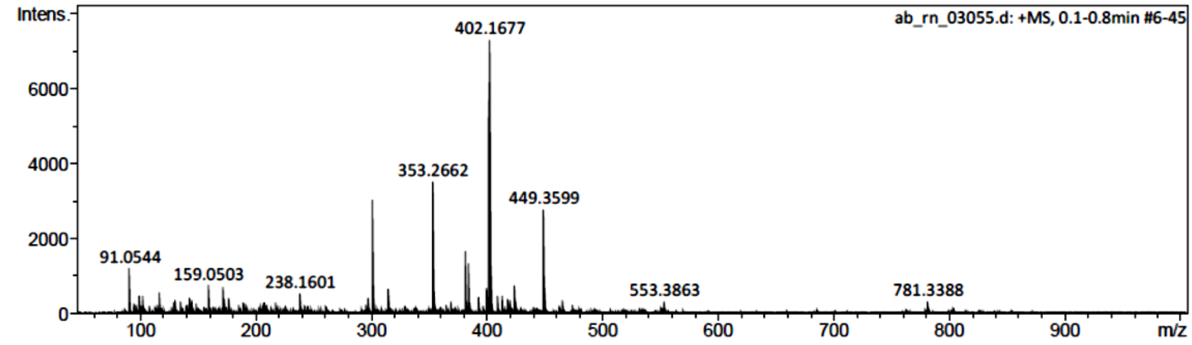

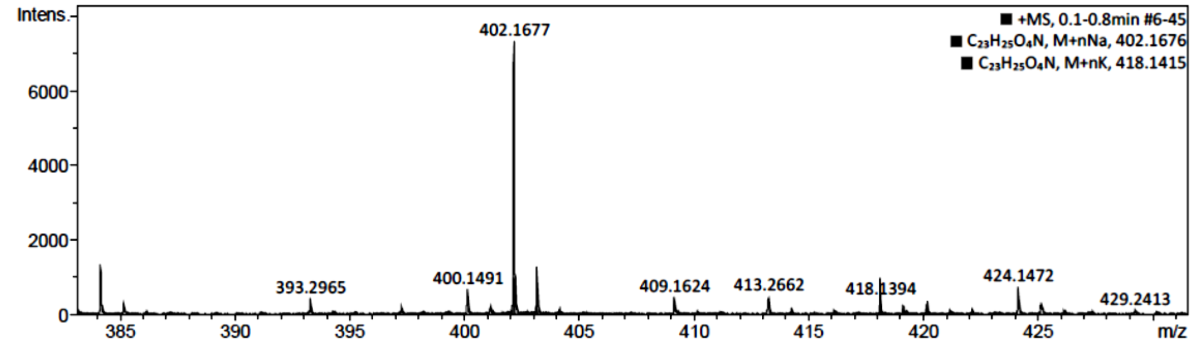

ab_rn_03055.d

Bruker Compass DataAnalysis 4.1 printed: 4/18/2022 12:40:33 PM by: IISER Kolkata Page 1 of 1

HRMS data of Xiamycin C [(+)-**2a**]

References:

1. S.-H. Kim, T-K.-Q. Ha, W. K. Oh, J. Shin and D.-C. Oh, *J. Nat. Prod*. 2016, **79**, 51.
2. D. H. Dethe and M. Shukla, *Chem. Commun.* 2021, **57**, 10644.
3. Q. Zhang, H. Li, L. Yu, Y. Sun, Y. Zhu, H. Zhu, L. Zhang, S. Li, Y. Shen, C. Tian, A. Li, H. Liu and C. Zhang, *Chem. Sci.* 2017, **8**, 5067.
4. M. Pfaffenbach, I. Bakanas, N. R. O Connor, J. L. Herrick and R. Sarpong, *Angew. Chem. Int. Ed.* 2019, **58**, 15304.
